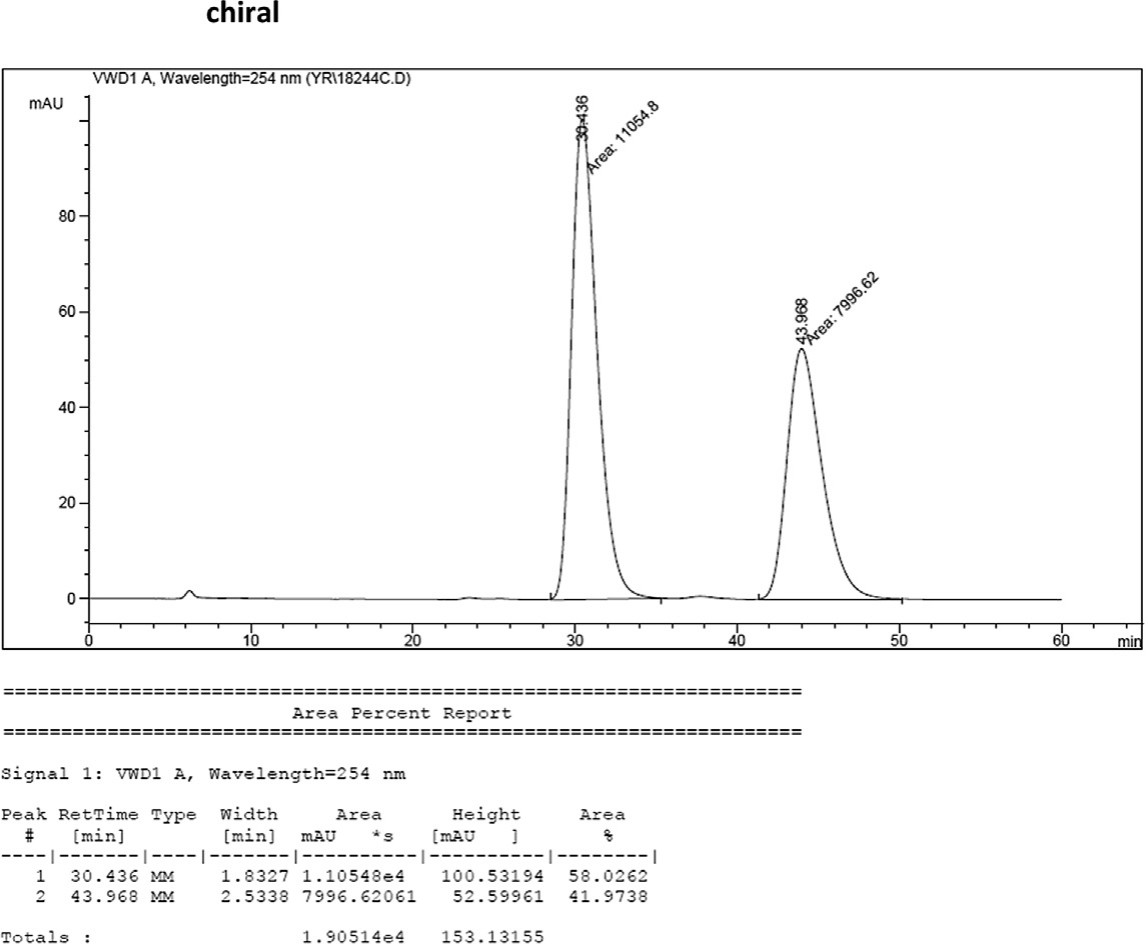# Data on for the synthesis of a new enantioenriched bisindolylmethane sulfamate derivatives

**DOI:** 10.1016/j.dib.2019.104549

**Published:** 2019-09-23

**Authors:** Sung-Gon Kim

**Affiliations:** Department of Chemistry, Kyonggi University, 154-42 Gwanggyosan-ro, Yeongtong-gu, Suwon 16227, Republic of Korea

**Keywords:** Bisindolylmethane, Sulfamidate, Friedel‒Crafts reaction

## Abstract

This data articles is related to the research paper entitled “Chiral Brønsted acid-catalyzed Friedel‒Crafts reaction of 3-indolylsulfamidates with Indoles: Synthesis of enantioenriched bisindolylmethane sulfamates” [1]. Here we introduce data acquired from the synthesis of bisindolylmethane sulfamates. The data include ^1^H and ^13^C NMR spectra, IR spectra, high-resolution Mass spectra, optical rotations, and melting points for the bisindolylmethane sulfamate derivatives described in [1]. The HPLC spectra of both racemic and chiral bisindolylmethane sulfamates are also provided in the article.

**Table:** Specifications Table

Subject area	*Chemistry*
More specific subject area	*Organic synthesis. Asymmetric catalysis*
Type of data	*Synthetic scheme, NMR and HPLC spectra*
How data was acquired	*NMR (Bruker/Ascend 400 spectrometer), IR (Rudolph/Autopol I FTIR spectrophotometer), High Mass (JEOL/JMS-700 spectrometer), HPLC (Agilent/1200 HPLC instrument)*
Data format	*Raw and analyzed*
Experimental factors	*The new bisindolylmethane sulfamate derivatives were synthesized by the asymmetric catalysis*
Experimental features	*The synthesized bisindolylmethane sulfamate derivatives were characterized by NMR, IR, and HPLC.*
Data source location	*Suwon, Republic of Korea.*
Data accessibility	*Data are available with the article.*
Related research article	Y. Kim, J. Lee, J. Jung, S.-G. Kim, Chiral Brønsted acid-catalyzed Friedel‒Crafts reaction of 3-indolylsulfamidates with Indoles: Synthesis of enantioenriched bisindolylmethane sulfamates, Tetrahedron Lett., 60, 2019, 1625–1630 [1].

**Table:** 

**Value of the data** • The data presents NMR, IR, and HPLC spectra of newly synthesized bisindolylmethane sulfamate derivatives which could be used by other researchers studying synthesis of sufamates. • The provided structural data could be useful for the analysis of spectra and determination of the structure of other bisindolylmethane sulfamate derivative. • The data could be useful for comparison with similar heterocyclic structures.

## 1 Data

**Scheme 1:**
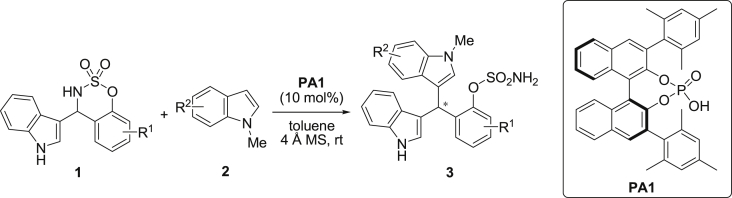
Synthesis of bisindolylmethane sulfamate product **3**.

**Fig. 1:**
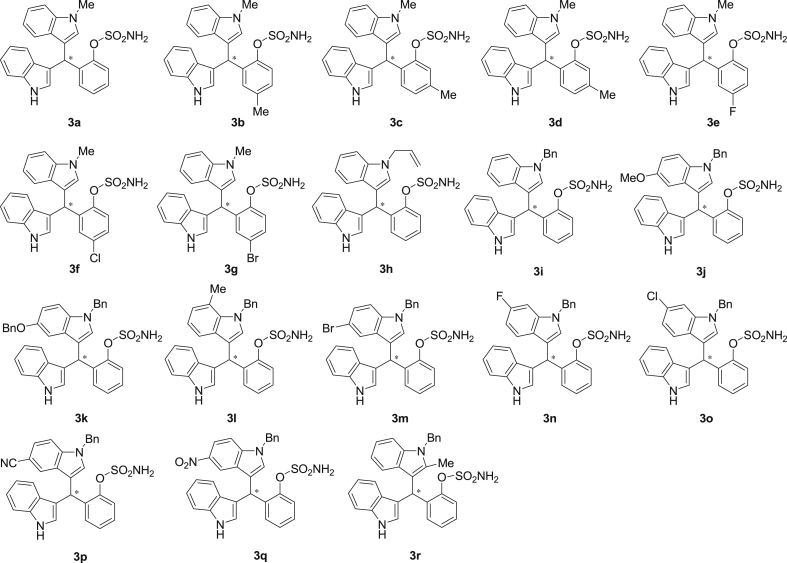
Bisindolylmethane sulfamate product **3**.

The dataset presented in this article focused on characterization of the new sulfamate derivatives described in [1]. Scheme 1 illustrates the synthesis of bisindolylmethane sulfamates **3** from the asymmetric catalytic Friedel‒Crafts reaction of 3-indolylsulfamidates **1** with indoles **2**. BINOL-derived phosphoric acid (**PA1**) was used as the catalyst in this asymmetric reaction. Newly synthesized bisindolylmethane sulfamate derivatives **3** (Fig. 1) were characterized using ^1^H and ^13^C NMR spectra, IR spectra, and HR mass spectrometry. The data also include m.p. and optical rotation, and the HPLC spectra of both racemic and chiral bisindolylmethane sulfamates for the determination of enantiomeric excess.

## 2 Experimental design, materials, and methods

### 2.1 General information

All reactions were performed in oven-dried glassware. All reagents were purchased from commercial suppliers and used without further purification. Organic solvents were purified and dried prior to use. Organic solutions were concentrated under reduced pressure on a rotary evaporator or an oil pump. Reactions were monitored through thin-layer chromatography (TLC) on EM Reagents 0.25 mm silica gel 60-F plates. Developed chromatograms were visualized by fluorescence quenching and with anisaldehyde stain. Flash column chromatography was accomplished using forced-flow chromatography on ICN 60 32–64 mesh silica gel 63.^1^H and ^13^C NMR spectra were recorded on the Bruker 400 spectrometer. TMS was used as the internal standard for ^1^H NMR (7.26 ppm), and the solvent signal was used as reference for ^13^C NMR (CDCl_3_, 77.16 ppm). Data for ^1^H NMR are reported as follows: chemical shift (*δ* ppm), multiplicity (s = singlet, d = doublet, t = triplet, q = quartet, m = multiplet), coupling constant (Hz) and integration. Data for ^13^C NMR are reported in terms of chemical shift. Infrared spectra (IR) spectra were recorded on an FT IR spectrometer and reported as wavelength numbers (cm^−1^). Optical rotations were taken on a digital polarimeter. High-resolution mass spectroscopy (HRMS) measurements were performed by electron impact (EI). Enantiomeric excesses were determined using an HPLC instrument with Chiralpak columns as noted.

### 2.2 General procedure for asymmetric synthesis of bisindolylmethane sulfamates

To a suspension of the 3-indolylsulfamidate **1** (0.10 mmol) and 4Å molecular sieves (20 mg) in toluene (0.2 M) was added catalyst **PA1** (0.010 mmol) at room temperature. After stirring for 10 min, indole **2** (0.10 mmol) was added in one portion. The reaction mixture was stirred at room temperature until TLC analysis revealed that the reaction no longer proceeded. Then, the resulting mixture was quenched with water and extracted with CH_2_Cl_2_. The combined organic layer was washed with brine, dried over anhydrous magnesium sulfate, filtered, and concentrated in vacuo. Purification via flash column chromatography (EtOAc/hexanes) provided the bisindolylmethane sulfamate compounds **3**. The enantiomeric excess was determined using HPLC analysis.

**Figure:**
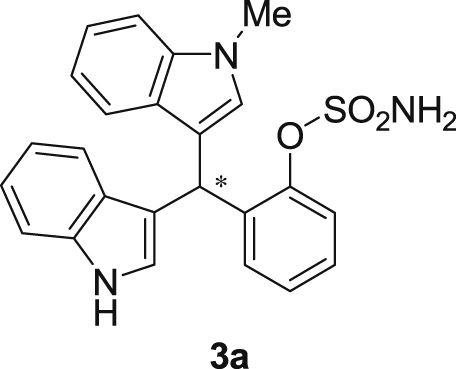

#### 2.2.1 2-((1*H*-Indol-3-yl)(1-methyl-1*H*-indol-3-yl)methyl)phenyl sulfamate (3a)

Red solid; m.p. 129–131 °C; ${\left[\text{α}\right]}_{\text{D}}^{26}$ = +3.7 (*c* = 0.46, EtOAc); 80:20 er; ^1^H NMR (400 MHz, CDCl_3_) *δ* 8.00 (s, 1H), 7.50 (d, *J* = 8.1 Hz, 1H), 7.45–7.34 (m, 3H), 7.32–7.14 (m, 6H), 7.02 (dd, *J* = 13.9, 7.0 Hz, 2H), 6.76 (s, 1H), 6.62 (s, 1H), 6.27 (s, 1H), 3.89 (s, 2H), 3.69 (s, 3H);^13^C NMR (100 MHz, CDCl_3_) *δ* 148.7, 137.3, 136.5, 136.3, 130.5, 128.7, 127.9, 126.91, 126.88, 126.5, 124.0, 122.4, 121.9, 121.8, 119.8, 119.7, 119.6, 119.2, 118.2, 116.3, 111.4, 109.4, 33.8, 32.8; IR (film) 3403, 3274, 2992, 1480, 1455, 1371, 1329, 1182, 1153, 1108, 919 cm^−1^; HRMS (EI) m/z calcd for [M]^+^ C_24_H_20_N_3_O_3_S: 430.1225 Found: 430.1229; Chiralpak AD-H column and AD-H guard column (15% *i*-PrOH:hexanes, 1.0 mL/min flow, λ = 254 nm); *minor*-isomer *t*_r_ = 38.2 min and *major*-isomer *t*_r_ = 61.5 min.

**Figure:**
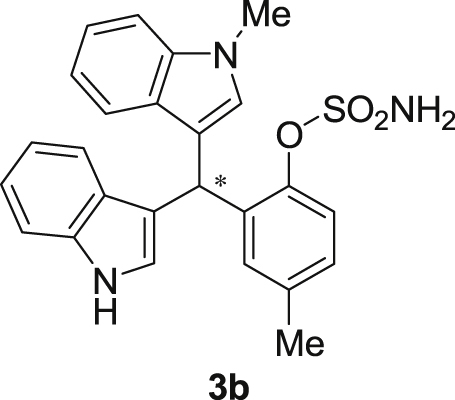

#### 2.2.2 2-((1*H*-Indol-3-yl)(1-methyl-1*H*-indol-3-yl)methyl)-4-methylphenyl sulfamate (3b)

Red solid; m.p. 124–127 °C; ${\left[\text{α}\right]}_{\text{D}}^{26}$ = −3.1 (*c* = 0.44, EtOAc); 64; 36 er; ^1^H NMR (400 MHz, CDCl_3_) *δ* 7.97 (s, 1H), 7.47–7.29 (m, 5H), 7.20 (dt, *J* = 14.7, 7.5 Hz, 2H), 7.11 (s, 1H), 7.09–6.98 (m, 3H), 6.74 (s, 1H), 6.62 (s, 1H), 6.23 (s, 1H), 3.88 (s, 2H), 3.68 (s, 3H), 2.22 (s, 3H); ^13^C NMR (100 MHz, CDCl_3_) *δ* 146.5, 137.3, 136.7, 136.5, 135.8, 130.8, 128.7, 128.5, 126.9, 126.5, 124.0, 122.4, 121.9, 121.6, 119.8, 119.7, 119.6, 119.2, 118.3, 116.4, 111.4, 109.4, 33.7, 32.8, 21.1; IR (film) 3404, 3284, 2923, 1547, 1485, 1457, 1421, 1372, 1330, 1198, 1169, 1089, 1012, 935 cm^−1^; HRMS (EI) m/z calcd for [M]^+^ C_25_H_22_N_3_O_3_S: 444.1382 Found: 444.1378; Chiralpak IA column and IA guard column (5% EtOH:hexanes, 1.0 mL/min flow, λ = 254 nm); *major*-isomer *t*_r_ = 63.5 min and *minor*-isomer *t*_r_ = 36.5 min.

**Figure:**
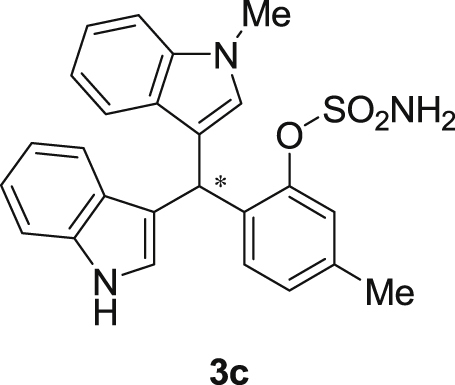

#### 2.2.3 2-((1*H*-Indol-3-yl)(1-methyl-1*H*-indol-3-yl)methyl)-5-methylphenyl sulfamate (3c)

Red solid; m.p. 114–117 °C;$\;{\left[\text{α}\right]}_{\text{D}}^{26}$ = +3.4 (*c* = 0.45, EtOAc); 65:35 er; ^1^H NMR (400 MHz, CDCl_3_) *δ* 7.96 (s, 1H), 7.41 (dd, *J* = 13.5, 8.0 Hz, 2H), 7.32 (t, *J* = 7.1 Hz, 3H), 7.25–7.15 (m, 3H), 7.02 (dd, *J* = 12.5, 7.3 Hz, 2H), 6.96 (d, *J* = 7.7 Hz, 1H), 6.72 (s, 1H), 6.62 (s, 1H), 6.21 (s, 1H), 3.91 (s, 2H), 3.67 (s, 3H), 2.33 (s, 3H); ^13^C NMR (100 MHz, CDCl_3_) *δ* 148.4, 138.1, 137.3, 136.5, 133.1, 130.2, 128.7, 127.7, 126.9, 126.5, 124.0, 122.4, 122.2, 121.9, 119.8, 119.74, 119.69, 119.2, 118.3, 116.5, 111.4, 109.4, 33.5, 32.8, 21.0; IR (film) 3406, 3274, 2920, 2850, 1712, 1616, 1499, 1457, 1421, 1372, 1329, 1182, 1149, 1083, 1011, 945 cm^−1^; HRMS (EI) m/z calcd for [M]^+^ C_25_H_22_N_3_O_3_S: 444.1382 Found: 444.1373; Chiralpak IA column and IA guard column (20% *i*-PrOH:hexanes, 1.0 mL/min flow, λ = 254 nm); *minor*-isomer *t*_r_ = 17.8 min and *major*-isomer *t*_r_ = 27.4 min.

**Figure:**
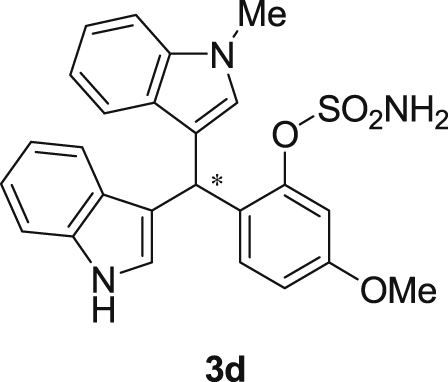

#### 2.2.4 2-((1*H*-Indol-3-yl)(1-methyl-1*H*-indol-3-yl)methyl)-5-methoxyphenyl sulfamate (3d)

Red solid; m.p. 129–132 °C; ${\left[\text{α}\right]}_{\text{D}}^{25}$ = −3.4 (*c* = 0.41, EtOAc); 56:44 er; ^1^H NMR (400 MHz, CDCl_3_) *δ* 7.97 (s, 1H), 7.40 (dd, *J* = 13.2, 7.9 Hz, 2H), 7.35–7.28 (m, 2H), 7.25–7.14 (m, 3H), 7.06 (d, *J* = 2.5 Hz, 1H), 7.02 (td, *J* = 7.3, 5.0 Hz, 2H), 6.73 (s, 1H), 6.70 (dd, *J* = 8.6, 2.5 Hz, 1H), 6.61 (s, 1H), 6.17 (s, 1H), 3.91 (s, 2H), 3.76 (s, 3H), 3.67 (s, 3H); ^13^C NMR (100 MHz, CDCl_3_) *δ* 158.9, 148.9, 137.3, 136.5, 130.9, 128.6, 128.2, 126.9, 126.4, 123.9, 122.4, 121.9, 119.8, 119.75, 119.72, 119.2, 118.5, 116.6, 112.9, 111.4, 109.4, 107.4, 55.6, 33.3, 32.8; IR (film) 3044, 3274, 3064, 2927, 1614, 1579, 1498, 1457, 1371, 1329, 1281, 1245, 1182, 1083, 1031, 944 cm^−1^; HRMS (EI) m/z calcd for [M]^+^ C_25_H_22_N_3_O_4_S: 460.1331 Found: 460.1346; Chiralpak IC column and IC guard column (10% *i*-PrOH:hexanes, 1.0 mL/min flow, λ = 254 nm); *minor*-isomer *t*_r_ = 42.9 min and *major*-isomer *t*_r_ = 54.1 min.

**Figure:**
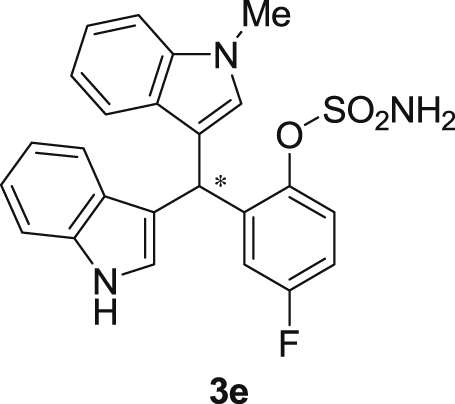

#### 2.2.5 2-((1*H*-Indol-3-yl)(1-methyl-1*H*-indol-3-yl)methyl)-4-fluorophenyl sulfamate (3e)

Red solid; m.p. 125–128 °C; ${\left[\text{α}\right]}_{\text{D}}^{23}$ = +1.1 (*c* = 0.43, EtOAc); 90:10 er; ^1^H NMR (400 MHz, acetone-d_6_) *δ* 9.91 (s, 1H), 7.42–7.30 (m, 3H), 7.28–7.20 (m, 2H), 7.20 (s, 2H), 7.03–6.86 (m, 3H), 6.86–6.73 (m, 3H), 6.68 (d, *J* = 1.7 Hz, 1H), 6.58 (s, 1H), 6.29 (s, 1H), 3.59 (s, 3H); ^13^C NMR (100 MHz, acetone-d_6_) *δ* 160.5 (d, *J*^*1*^ = 242.8 Hz), 144.8 (d, *J*^*4*^ = 2.7 Hz), 141.0 (d, *J*^*3*^ = 7.1 Hz), 137.6, 137.2, 128.5, 127.3, 126.9, 124.2, 123.6 (d, *J*^*3*^ = 8.9 Hz), 121.4 (d, *J*^*3*^ = 7.3 Hz), 119.9, 119.6, 118.6, 118.5, 117.1, 116.4, 116.23, 116.21, 113.6 (d, *J*^*2*^ = 23.8 Hz), 111.3, 109.3, 33.8, 31.9; IR (film) 3048, 3277, 2921, 2851, 1615, 1540, 1479, 1459, 1421, 1370, 1338, 1263, 1183, 1159, 1064, 1010, 938 cm^−1^; HRMS (EI) m/z calcd for [M]^+^ C_24_H_19_FN_3_O_3_S: 464.0836 Found: 464.0839; Chiralpak IC column and IC guard column (10% *i*-PrOH:hexanes, 1.0 mL/min flow, λ = 254 nm); *minor*-isomer *t*_r_ = 29.6 min and *major*-isomer *t*_r_ = 34.4 min.

**Figure:**
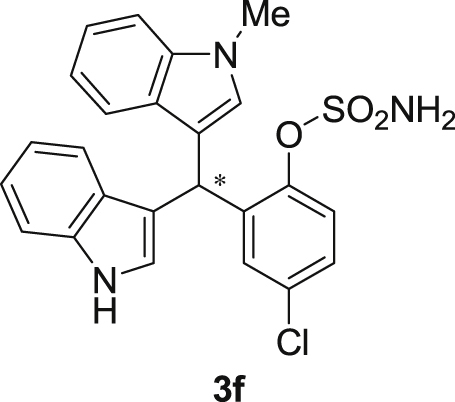

#### 2.2.6 2-((1*H*-Indol-3-yl)(1-methyl-1*H*-indol-3-yl)methyl)-4-chlorophenyl sulfamate (3f)

Red solid; m.p. 130–134 °C; ${\left[\text{α}\right]}_{\text{D}}^{26}$ = +1.9 (*c* = 0.42, EtOAc); 94; 6 er; ^1^H NMR (400 MHz, CDCl_3_) *δ* 7.96 (s, 1H), 7.44–7.35 (m, 3H), 7.31 (dd, *J* = 11.2, 8.3 Hz, 2H), 7.24–7.15 (m, 4H), 7.02 (dd, *J* = 12.4, 7.3 Hz, 2H), 6.69 (s, 1H), 6.58 (s, 1H), 6.21 (s, 1H), 4.00 (s, 2H), 3.66 (s, 3H); ^13^C NMR (100 MHz, CDCl_3_) *δ* 147.0, 138.5, 137.3, 136.5, 132.5, 130.1, 128.7, 128.0, 126.7, 126.3, 124.0, 123.2, 122.6, 122.1, 120.0, 119.5, 119.42, 119.39, 117.5, 115.6, 111.5, 109.6, 33.6, 32.8; IR (film) 3404, 3277, 2923, 1615, 1544, 1471, 1457, 1372, 1330, 1182, 1156, 1099, 1011, 924 cm^−1^; HRMS (EI) m/z calcd for [M]^+^ C_24_H_19_ClN_3_O_3_S: 448.1131 Found: 448.1121; Chiralpak IB column and IB guard column (20% *i*-PrOH:hexanes, 1.0 mL/min flow, λ = 254 nm); *major*-isomer *t*_r_ = 23.8 min and *minor*-isomer *t*_r_ = 29.6 min.

**Figure:**
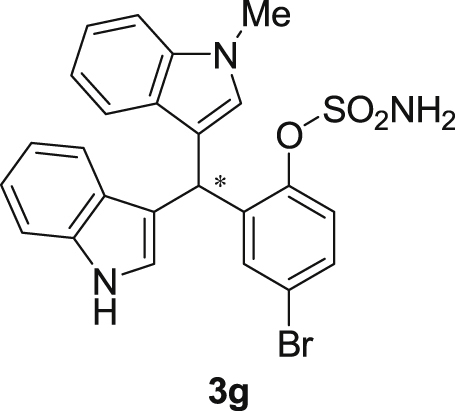

#### 2.2.7 2-((1*H*-Indol-3-yl)(1-methyl-1*H*-indol-3-yl)methyl)-4-bromophenyl sulfamate (3g)

Red solid; m.p. 115–118 °C; ${\left[\text{α}\right]}_{\text{D}}^{27}$ = +5.9 (*c* = 0.50, EtOAc); 93:7 er; ^1^H NMR (400 MHz, CDCl_3_) *δ* 8.02 (s, 1H), 7.46–7.30 (m, 7H), 7.22 (dd, *J* = 17.8, 7.7 Hz, 2H), 7.05 (dd, *J* = 13.9, 6.9 Hz, 2H), 6.76 (s, 1H), 6.61 (s, 1H), 6.23 (s, 1H), 3.95 (s, 2H), 3.70 (s, 3H); ^13^C NMR (100 MHz, CDCl_3_) *δ* 147.6, 138.8, 137.3, 136.5, 133.0, 131.0, 128.7, 126.7, 126.3, 124.0, 123.6, 122.6, 122.1, 120.4, 120.0, 119.4 (two peaks overlapping), 119.4, 117.6, 115.6, 111.5, 109.5, 33.6, 32.8; IR (film) 3045, 3276, 2921, 2852, 1713, 1614, 1545, 1468, 1373, 1330, 1268, 1181, 1155, 1094, 1012, 925 cm^−1^; HRMS (EI) m/z calcd for [M]^+^ C_24_H_19_BrN_3_O_3_S: 508.0330 Found: 508.0315; Chiralpak IB column and IB guard column (20% *i*-PrOH:hexanes, 1.0 mL/min flow, λ = 254 nm); *major*-isomer *t*_r_ = 24.1 min and *minor*-isomer *t*_r_ = 30.9 min.

**Figure:**
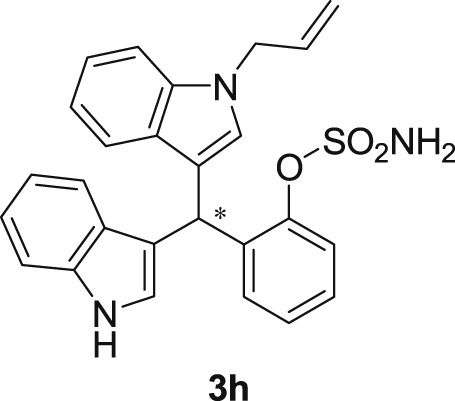

#### 2.2.8 2-((1-Allyl-1*H*-indol-3-yl)(1*H*-indol-3-yl)methyl)phenyl sulfamate (3h)

Red solid; m.p. 178–180 °C; ${\left[\text{α}\right]}_{\text{D}}^{25}$ = +1.9 (*c* = 0.44, EtOAc); 69:31 er; ^1^H NMR (400 MHz, CDCl_3_) *δ* 7.97 (s, 1H), 7.48 (d, *J* = 8.1 Hz, 1H), 7.43–7.36 (m, 2H), 7.34 (d, *J* = 8.2 Hz, 1H), 7.31–7.23 (m, 3H), 7.21–7.10 (m, 3H), 7.01 (dd, *J* = 13.6, 6.8 Hz, 2H), 6.73 (s, 1H), 6.66 (s, 1H), 6.26 (s, 1H), 5.91 (ddd, *J* = 22.3, 10.4, 5.3 Hz, 1H), 5.14 (dd, *J* = 10.2, 1.1 Hz, 1H), 5.03 (dd, *J* = 17.1, 1.1 Hz, 1H), 4.62 (d, *J* = 5.3 Hz, 2H), 3.87 (s, 2H); ^13^C NMR (100 MHz, CDCl_3_) *δ* 148.6, 136.7, 136.5, 136.2, 133.4, 130.5, 127.9, 127.8, 127.1, 126.9, 126.5, 124.0, 122.4, 122.0, 121.8, 119.8 (two peaks overlapping), 119.6, 119.4, 118.1, 117.2, 116.7, 111.4, 109.9, 48.8, 33.8; IR (film) 3404, 3278, 2922, 1728, 1612, 1544, 1480, 1419, 1366, 1388, 1201, 1178, 1148, 1085, 938 cm^−1^; HRMS (EI) m/z calcd for [M]^+^ C_26_H_27_N_3_O_3_S: 457.1460 Found: 457.1445; Chiralpak AD-H column and AD-H guard column (20% EtOH:hexanes, 1.0 mL/min flow, λ = 254 nm); *minor*-isomer *t*_r_ = 15.1 min and *major*-isomer *t*_r_ = 22.5 min.

**Figure:**
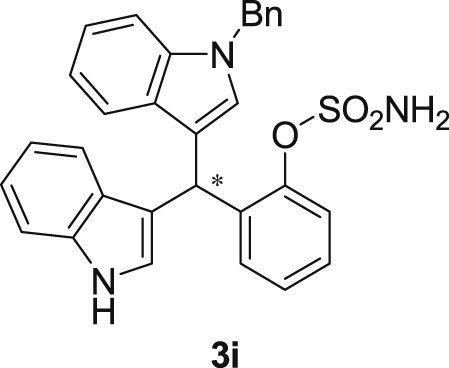

#### 2.2.9 2-((1-Benzyl-1*H*-indol-3-yl)(1*H*-indol-3-yl)methyl)phenyl sulfamate (3i)

Red solid; m.p. 173–175 °C; ${\left[\text{α}\right]}_{\text{D}}^{23}$ = +9.3 (*c* = 0.44, EtOAc); 63:37 er; ^1^H NMR (400 MHz, CDCl_3_) *δ* 7.90 (s, 1H), 7.45 (d, *J* = 8.0 Hz, 1H), 7.42–7.35 (m, 2H), 7.30–7.18 (m, 7H), 7.13 (dd, *J* = 13.4, 5.9 Hz, 3H), 7.06–6.93 (m, 4H), 6.70 (s, 1H), 6.65 (s, 1H), 6.27 (s, 1H), 5.16 (s, 2H), 3.91 (s, 2H); ^13^C NMR (100 MHz, CDCl_3_) *δ* 148.6, 137.6, 136.9, 136.5, 136.2, 130.5, 128.8, 128.3, 127.9, 127.6, 127.2, 127.0, 126.6, 126.5, 124.0, 122.4, 122.2, 121.8, 119.9, 119.8, 119.7, 119.5, 118.1, 117.0, 111.4, 110.1, 50.0, 33.8; IR (film) 3401, 3275, 2924, 1544, 1479, 1453, 1370, 1334, 1200, 1182, 1152, 1083, 1011, 909 cm^−1^; HRMS (EI) m/z calcd for [M]^+^ C_30_H_25_N_3_O_3_S: 507.1617 Found: 507.1606; Chiralpak IB column and IB guard column (30% *i*-PrOH:hexanes, 1.0 mL/min flow, λ = 254 nm); *major*-isomer *t*_r_ = 13.1 min and *minor*-isomer *t*_r_ = 17.0 min.

**Figure:**
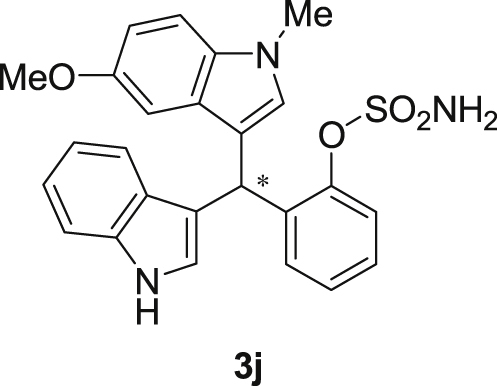

#### 2.2.10 2-((1*H*-Indol-3-yl)(5-methoxy-1-methyl-1*H*-indol-3-yl)methyl)phenyl sulfamate (3j)

Red solid; m.p. 187–191 °C; ${\left[\text{α}\right]}_{\text{D}}^{25}$ = +20.8 (*c* = 0.43, EtOAc); 88:12 er; ^1^H NMR (400 MHz, CDCl_3_) *δ* 8.01 (s, 1H), 7.49 (dd, *J* = 8.1, 1.1 Hz, 1H), 7.38 (dd, *J* = 14.5, 8.1 Hz, 2H), 7.28 (dt, *J* = 13.8, 3.8 Hz, 2H), 7.22–7.13 (m, 3H), 7.06–6.99 (m, 1H), 6.87 (dd, *J* = 8.8, 2.4 Hz, 1H), 6.85–6.82 (m, 1H), 6.75 (dt, *J* = 2.4 Hz, 1H), 6.61 (s, 1H), 6.21 (s, 1H), 3.91 (s, 2H), 3.70 (s, 3H), 3.66 (s, 3H); ^13^C NMR (100 MHz, CDCl_3_) *δ* 153.9, 148.6, 136.5, 136.3, 132.6, 130.5, 129.2, 127.9, 127.1, 126.9, 126.5, 124.0, 122.4, 121.8, 119.8, 119.7, 118.2, 115.9, 112.1, 111.4, 110.3, 101.3, 55.9, 33.8, 33.0; IR (film) 3413, 3266 2925, 1489, 1475, 1422, 1372, 1271, 1205, 1181, 1144, 1116, 1086, 1038, 909 cm^−1^; HRMS (EI) m/z calcd for [M]^+^ C_25_H_23_N_3_O_4_S: 461.1409 Found: 461.1406; Chiralpak AD-H column and AD-H guard column (20% EtOH:hexanes, 1.0 mL/min flow, λ = 254 nm); *major*-isomer *t*_r_ = 35.6 min and *minor*-isomer *t*_r_ = 55.6 min.

**Figure:**
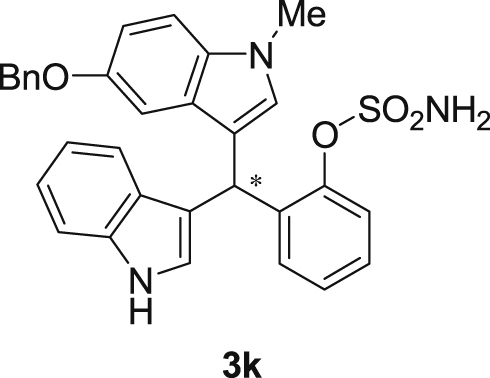

#### 2.2.11 2-((5-(Benzyloxy)-1-methyl-1*H*-indol-3-yl)(1*H*-indol-3-yl)methyl)phenyl sulfamate (3k)

Red solid; m.p. 122–124 °C; ${\left[\text{α}\right]}_{\text{D}}^{25}$ = +17.8 (*c* = 0.43, EtOAc); 85:15 er; ^1^H NMR (400 MHz, CDCl_3_) *δ* 7.96 (s, 1H), 7.48 (d, *J* = 7.9 Hz, 1H), 7.42–7.25 (m, 9H), 7.22–7.11 (m, 3H), 7.03 (t, *J* = 7.5 Hz, 1H), 6.96 (dd, *J* = 8.8, 2.2 Hz, 1H), 6.91 (d, *J* = 2.1 Hz, 1H), 6.70 (s, 1H), 6.60 (s, 1H), 6.18 (s, 1H), 4.96 (s, 2H), 3.83 (s, 2H), 3.64 (s, 3H); ^13^C NMR (100 MHz, CDCl_3_) *δ* 151.7, 147.6, 136.5, 135.4, 135.2, 131.6, 129.4, 128.3, 127.4, 126.8, 126.7, 126.6, 126.0, 125.9, 125.4, 123.0, 121.3, 120.7, 118.7, 118.6, 117.0, 114.6, 111.9, 110.3, 109.2, 102.0, 69.7, 32.8, 31.9; IR (film) 3393, 3275, 2919, 1487, 1455, 1424, 1374, 1339, 1259, 1182, 1153, 1084, 1011, 914 cm^−1^; HRMS (EI) m/z calcd for [M]^+^ C_31_H_27_N_3_O_4_S: 537.1722 Found: 537.1696; Chiralpak AD-H column and AD-H guard column (20% EtOH:hexanes, 1.0 mL/min flow, λ = 254 nm); *major*-isomer *t*_r_ = 47.0 min and *minor*-isomer *t*_r_ = 79.7 min.

**Figure:**
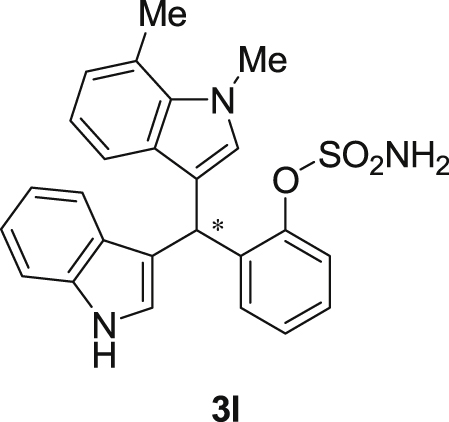

#### 2.2.12 2-((1*H*-Indol-3-yl)(1,7-dimethyl-1*H*-indol-3-yl)methyl)phenyl sulfamate (3l)

Red solid; m.p. 201–203 °C; ${\left[\text{α}\right]}_{\text{D}}^{25}$ = +1.6 (*c* = 0.50, EtOAc); 86:14 er; ^1^H NMR (400 MHz, CDCl_3_) *δ* 7.98 (s, 1H), 7.48 (dd, *J* = 8.1, 1.1 Hz, 1H), 7.38 (dd, *J* = 18.6, 8.1 Hz, 2H), 7.29–7.24 (m, 2H), 7.17 (dtd, *J* = 16.1, 7.3, 1.2 Hz, 3H), 7.06–6.99 (m, 1H), 6.92–6.83 (m, 2H), 6.75 (d, *J* = 1.6 Hz, 1H), 6.49 (s, 1H), 6.21 (s, 1H), 3.94 (s, 3H), 3.83 (s, 2H), 2.75 (s, 3H); ^13^C NMR (100 MHz, CDCl_3_) *δ* 148.6, 136.4, 136.2, 136.0, 130.5, 130.4, 127.9, 126.9, 126.5, 124.7, 124.0, 122.4, 121.8, 121.5, 119.8 (two peaks overlapping), 119.63, 119.57, 118.2, 117.7, 115.9, 111.3, 36.7, 33.6, 19.7; IR (film) 3398, 3281, 1558, 1541, 1507, 1475, 1456, 1265, 1208, 1180, 1149, 1087, 1058, 939 cm^−1^; HRMS (EI) m/z calcd for [M]^+^ C_25_H_23_N_3_O_3_S: 445.1460 Found: 445.1489; Chiralpak AD-H column and AD-H guard column (10% EtOH:hexanes, 1.0 mL/min flow, λ = 254 nm); *minor*-isomer *t*_r_ = 49.5 min and *major*-isomer *t*_r_ = 63.3 min.

**Figure:**
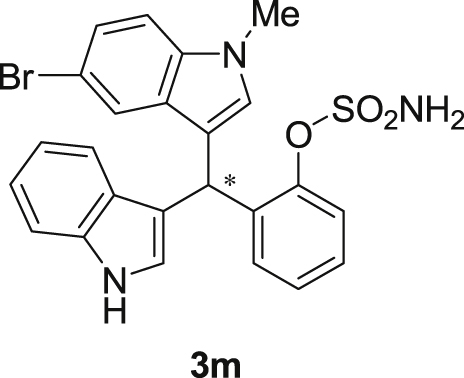

#### 2.2.13 2-((5-Bromo-1-methyl-1*H*-indol-3-yl)(1*H*-indol-3-yl)methyl)phenyl sulfamate (3m)

Red solid; m.p. 197–199 °C; ${\left[\text{α}\right]}_{\text{D}}^{23}$ = −2.6 (*c* = 0.44, EtOAc); 89:11 er; ^1^H NMR (400 MHz, acetone-d_6_) *δ* 9.91 (s, 1H), 7.49 (d, *J* = 1.6 Hz, 1H), 7.36 (dd, *J* = 8.1, 1.2 Hz, 1H), 7.28 (dd, *J* = 20.7, 8.0 Hz, 2H), 7.21–7.03 (m, 5H), 6.97–6.91 (m, 1H), 6.80–6.71 (m, 1H), 6.61 (d, *J* = 1.6 Hz, 1H), 6.56 (s, 1H), 6.26 (s, 1H), 3.62 (s, 3H), 2.71 (s, 2H); ^13^C NMR (100 MHz, acetone-d_6_) *δ* 148.8, 137.5, 137.2, 136.3, 130.0 (two peaks overlapping), 129.1, 127.3, 126.9, 126.2, 124.1, 123.8, 122.3, 121.9, 121.3, 119.7, 118.6, 117.4, 116.7, 111.6, 111.3, 111.2, 33.3, 32.1; IR (film) 3424, 3367, 3261, 2921, 1731, 1545, 1475, 1454, 1419, 1369, 1293, 1179, 1122, 1085, 1043, 1010, 930 cm^−1^; HRMS (EI) m/z calcd for [M]^+^ C_24_H_20_BrN_3_O_3_S: 509.0409 Found: 509.0392; Chiralpak AD-H column and AD-H guard column (10% *i*-PrOH:hexanes, 1.0 mL/min flow, λ = 254 nm); *minor*-isomer *t*_r_ = 22.6 min and *major*-isomer *t*_r_ = 31.9 min.

**Figure:**
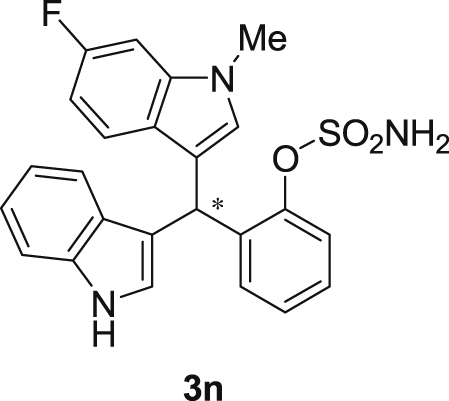

#### 2.2.14 2-((6-Fluoro-1-methyl-1*H*-indol-3-yl)(1*H*-indol-3-yl)methyl)phenyl sulfamate (3n)

Red solid; m.p. 197–198 °C; ${\left[\text{α}\right]}_{\text{D}}^{21}$ = +0.8 (*c* = 0.50, EtOAc); 72:28 er; ^1^H NMR (400 MHz, acetone-d_6_) *δ* 7.52–7.36 (m, 4H), 7.33–7.22 (m, 4H), 7.18–7.04 (m, 3H), 6.89 (ddd, *J* = 8.0, 7.1, 1.0 Hz, 1H), 6.77 (dd, *J* = 2.4, 0.8 Hz, 1H), 6.71 (ddd, *J* = 9.8, 8.7, 2.3 Hz, 1H), 6.66 (d, *J* = 0.9 Hz, 1H), 6.42 (s, 1H), 3.71 (s, 3H); ^13^C NMR (100 MHz, acetone-d_6_) *δ* 159.7 (d, *J*^*1*^ = 235.1 Hz), 148.8, 137.6 (d, *J*^*4*^ = 12.2 Hz), 137.5 (d, *J*^*2*^ = 58.0 Hz), 130.1, 129.1, 129.0, 127.2, 127.0, 126.2, 124.2, 124.1, 121.8, 121.3, 121.0 (d, *J*^*4*^ = 10.1 Hz), 119.8, 118.56, 117.6, 117.4, 111.3, 106.7 (d, *J*^*3*^ = 24.5 Hz), 95.5 (d, *J*^*3*^ = 26.2 Hz), 33.4, 32.0; IR (film) 3412, 3279, 2924, 1618, 1557, 1477, 1458, 1368, 1335, 1240, 1180, 1150, 1099, 1087, 1049, 944 cm^−1^; HRMS (EI) m/z calcd for [M]^+^ C_24_H_20_FN_3_O_3_S: 449.1209 Found: 449.1216; Chiralpak IC column and IC guard column (25% *i*-PrOH:hexanes, 1.0 mL/min flow, λ = 254 nm); *minor*-isomer *t*_r_ = 8.0 min and *major*-isomer *t*_r_ = 16.2 min.

**Figure:**
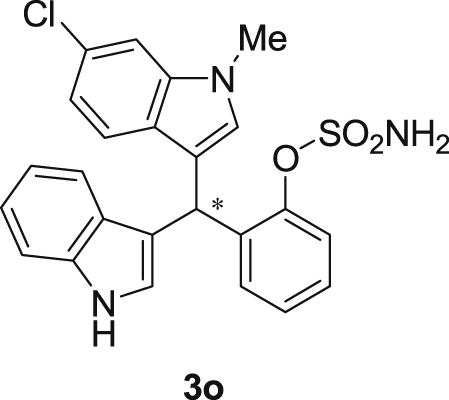

#### 2.2.15 2-((6-Chloro-1-methyl-1*H*-indol-3-yl)(1*H*-indol-3-yl)methyl)phenyl sulfamate (3o)

White solid; m.p. 207–209 °C; ${\left[\text{α}\right]}_{\text{D}}^{25}$ = +2.3 (*c* = 0.44, EtOAc); 74:26 er; ^1^H NMR (400 MHz, acetone-d_6_) *δ* 9.91 (s, 1H), 7.39–7.33 (m, 1H), 7.33–7.23 (m, 4H), 7.22–7.08 (m, 3H), 7.02 (t, *J* = 7.0 Hz, 1H), 6.94 (t, *J* = 7.2 Hz, 1H), 6.80–6.71 (m, 2H), 6.63 (d, *J* = 1.8 Hz, 1H), 6.57 (s, 1H), 6.28 (s, 1H), 3.61 (s, 3H), 2.73 (s, 1H).; ^13^C NMR (100 MHz, acetone-d_6_) *δ* 148.7, 138.0, 137.7, 137.2, 130.0, 129.5, 127.3, 127.0, 126.8, 126.20, 126.17, 124.1, 121.8, 121.3, 121.2, 119.7, 118.7, 118.6, 117.5, 117.4, 111.3, 109.3, 33.4, 32.0; IR (film) 3419, 3276, 2926, 1540, 1479, 1456, 1372, 1329, 1203, 1180, 1149, 1125, 1086, 1047, 1009, 941 cm^−1^; HRMS (EI) m/z calcd for [M]^+^ C_24_H_20_ClN_3_O_3_S: 465.0914 Found: 465.0898; Chiralpak IC column and IC guard column (25% *i*-PrOH:hexanes, 1.0 mL/min flow, λ = 254 nm); *minor*-isomer *t*_r_ = 7.7 min and *major*-isomer *t*_r_ = 12.8 min.

**Figure:**
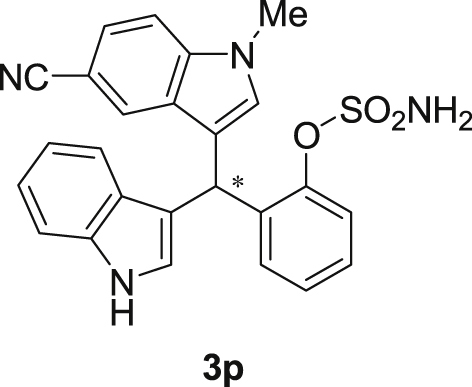

#### 2.2.16 2-((5-Cyano-1-methyl-1*H*-indol-3-yl)(1*H*-indol-3-yl)methyl)phenyl sulfamate (3p)

Yellow solid; m.p. 203–204 °C; ${\left[\text{α}\right]}_{\text{D}}^{29}$ = −3.5 (*c* = 0.42, EtOAc); 64:36 er; ^1^H NMR (400 MHz, acetone-d_6_) *δ* 10.1 (s, 1H), 7.90 (d, *J* = 0.9 Hz, 1H), 7.58–7.48 (m, 2H), 7.48–7.35 (m, 4H), 7.34–7.22 (m, 2H), 7.18 (dd, *J* = 10.8, 4.2 Hz, 1H), 7.12–7.04 (m, 1H), 6.91 (dd, *J* = 11.7, 4.6 Hz, 2H), 6.78 (s, 1H), 6.48 (s, 1H), 3.81 (s, 3H); ^13^C NMR (100 MHz, acetone-d_6_) *δ* 148.7, 139.0, 137.4, 137.1, 131.1, 129.9, 127.5, 127.2, 126.8, 126.4, 125.3, 124.0, 123.9, 122.0, 121.4, 120.4, 119.6, 118.7, 118.3, 117.1, 111.3, 110.7, 101.4, 33.3, 32.2; IR (film) 3336, 3242, 2919, 2221, 1558, 1487, 1455, 1382, 1365, 1205, 1182, 1084, 937 cm^−1^; HRMS (EI) m/z calcd for [M]^+^ C_25_H_20_N_4_O_3_S: 456.1256 Found: 456.1239; Chiralpak IC column and IC guard column (10% EtOH:hexanes, 1.0 mL/min flow, λ = 254 nm); *minor*-isomer *t*_r_ = 40.3 min and *major*-isomer *t*_r_ = 55.8 min.

**Figure:**
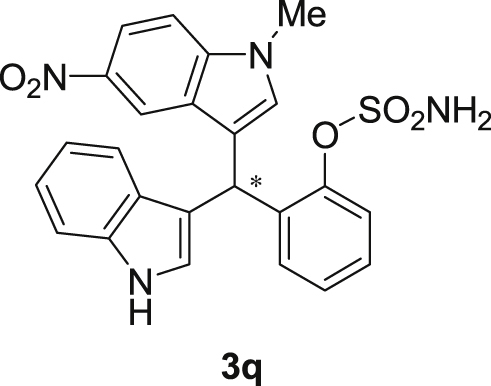

#### 2.2.17 2-((1*H*-Indol-3-yl)(1-methyl-5-nitro-1*H*-indol-3-yl)methyl)phenyl sulfamate (3q)

Yellow solid; m.p. 198–201 °C; ${\left[\text{α}\right]}_{\text{D}}^{29}$ = −13.4 (*c* = 0.41, EtOAc); 67:33 er; ^1^H NMR (400 MHz, acetone-d_6_) *δ* 10.07 (s, 1H), 8.47 (d, *J* = 2.2 Hz, 1H), 8.05 (dd, *J* = 9.1, 2.2 Hz, 1H), 7.58–7.49 (m, 2H), 7.42 (dd, *J* = 19.4, 8.1 Hz, 2H), 7.36 (s, 1H), 7.33–7.24 (m, 2H), 7.22–7.15 (m, 1H), 7.12–7.05 (m, 1H), 6.89 (dd, *J* = 11.5, 4.5 Hz, 2H), 6.80 (d, *J* = 1.6 Hz, 1H), 6.53 (s, 1H), 3.86 (s, 3H), 2.86 (s, 1H); ^13^C NMR (100 MHz, acetone-d_6_) *δ* 148.7, 141.0, 140.3, 137.3, 137.2, 132.0, 129.9, 127.6, 126.9, 126.6, 126.4, 124.3, 122.0, 121.5, 120.1, 119.7, 118.7, 117.1, 117.0, 116.6, 111.4, 109.8, 33.3, 32.5; IR (film) 3369, 3274, 3106, 2922, 1615, 1556, 1502, 1481, 1452, 1384, 1325, 1310, 1291, 1180, 1160, 1084, 1035, 938 cm^−1^; HRMS (EI) m/z calcd for [M]^+^ C_24_H_20_N_4_O_5_S: 476.1154 Found: 476.1129; Chiralpak AD-H column and AD-H guard column (20% *i*-PrOH:hexanes, 1.0 mL/min flow, λ = 254 nm); *minor*-isomer *t*_r_ = 44.5 min and *major*-isomer *t*_r_ = 79.9 min.

**Figure:**
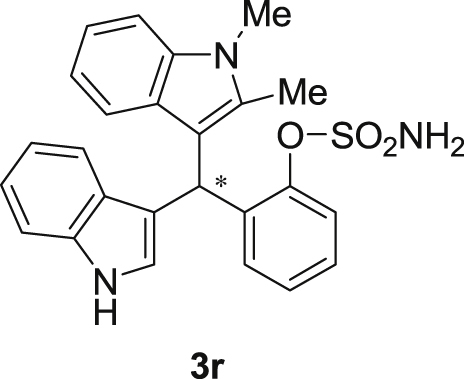

#### 2.2.17 2-((1*H*-Indol-3-yl)(1,2-dimethyl-1*H*-indol-3-yl)methyl)phenyl sulfamate (3r)

Red solid; m.p. 165–167 °C; ${\left[\text{α}\right]}_{\text{D}}^{25}$ = −5.8 (*c* = 0.42, EtOAc); 58:42 er; ^1^H NMR (400 MHz, CDCl_3_) *δ* 7.95 (s, 1H), 7.51 (dd, *J* = 8.1, 1.1 Hz, 1H), 7.36 (d, *J* = 8.2 Hz, 1H), 7.34 (dd, *J* = 7.7, 1.6 Hz, 1H), 7.29–7.23 (m, 4H), 7.21–7.07 (m, 3H), 7.05–6.97 (m, 1H), 6.87 (t, *J* = 7.1 Hz, 1H), 6.72 (d, *J* = 1.3 Hz, 1H), 6.16 (s, 1H), 3.70 (s, 3H), 3.36 (s, 2H), 2.39 (s, 3H); ^13^C NMR (100 MHz, CDCl_3_) *δ* 149.1, 136.7, 136.1, 135.8, 134.8, 131.1, 127.9, 126.81, 126.77, 126.5, 124.6, 122.3, 120.8, 120.7, 119.8, 119.6, 119.3, 119.1, 117.2, 111.5, 111.2, 109.2, 34.1, 29.8, 10.6; IR (film) 3406, 3280, 2821, 2851, 1678, 1552, 1469, 1455, 1366, 1335, 1251, 1182, 1153, 1086, 1017 cm^−1^; HRMS (EI) m/z calcd for [M]^+^ C_25_H_23_N_3_O_3_S: 445.1460 Found: 445.1487; Chiralpak IC column and IC guard column (10% *i*-PrOH:hexanes, 1.0 mL/min flow, λ = 254 nm); *major*-isomer *t*_r_ = 58.0 min and *minor*-isomer *t*_r_ = 44.0 min.

^1^H NMR (400 MHz) in CDCl_3_

**Figure:**
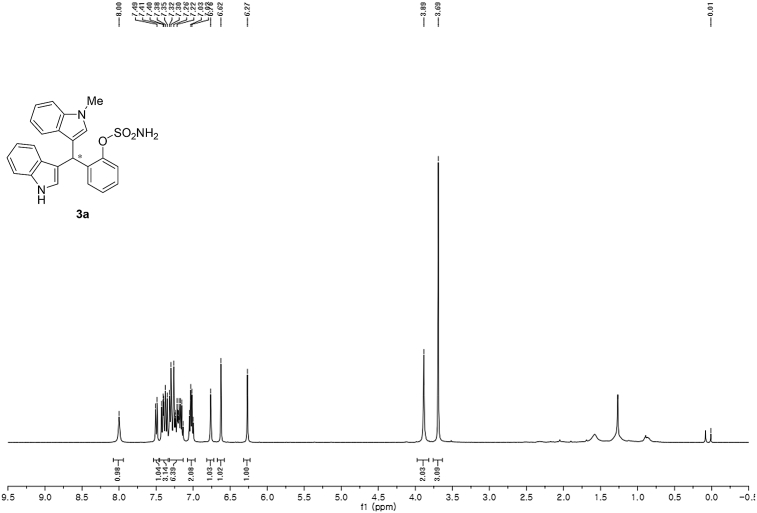

^13^C NMR (100 MHz) in CDCl_3_

**Figure:**
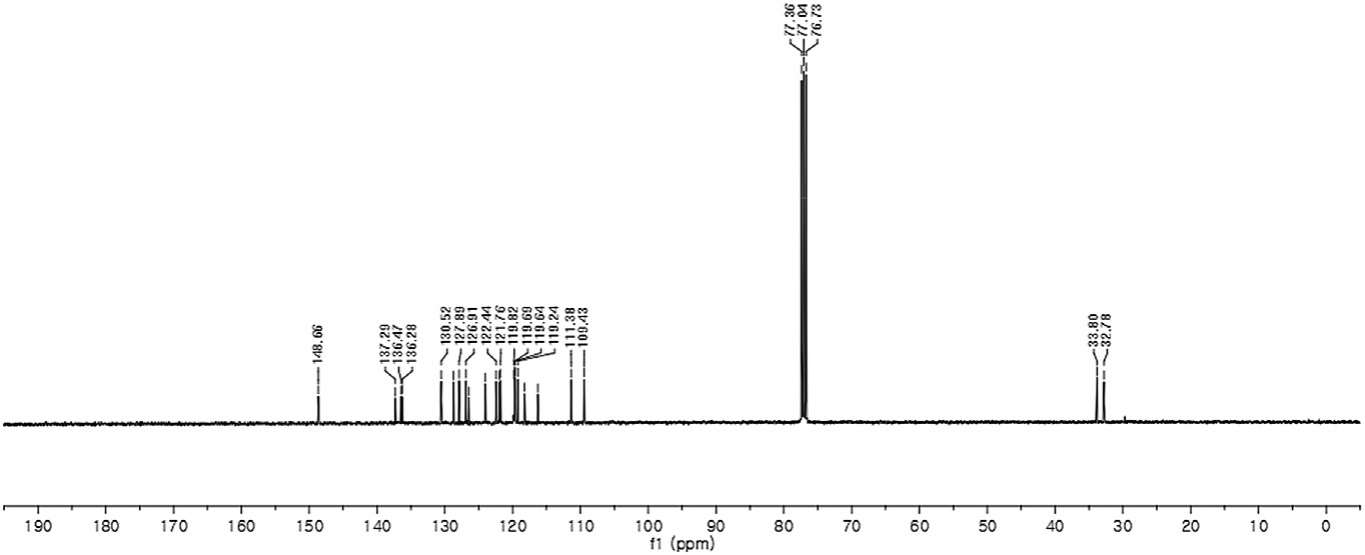

^1^H NMR (400 MHz) in CDCl_3_

**Figure:**
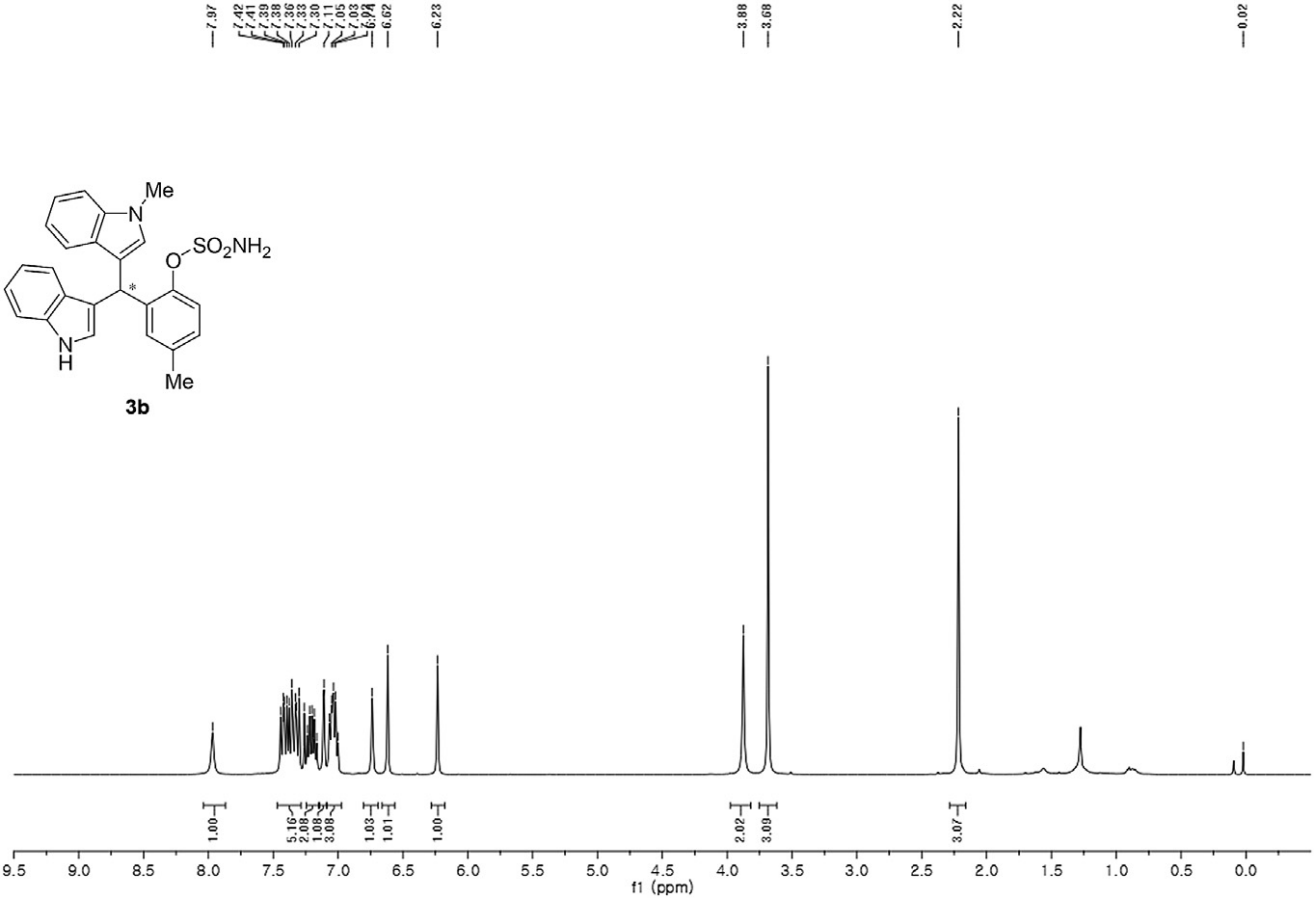

^13^C NMR (100 MHz) in CDCl_3_

**Figure:**
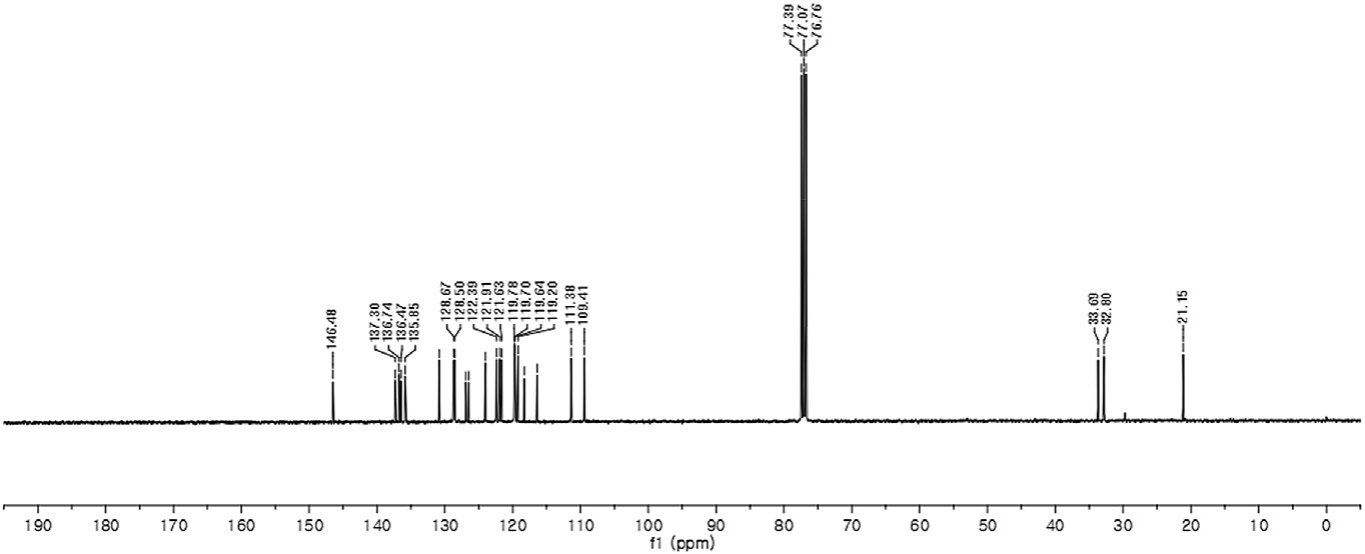

^1^H NMR (400 MHz) in CDCl_3_

**Figure:**
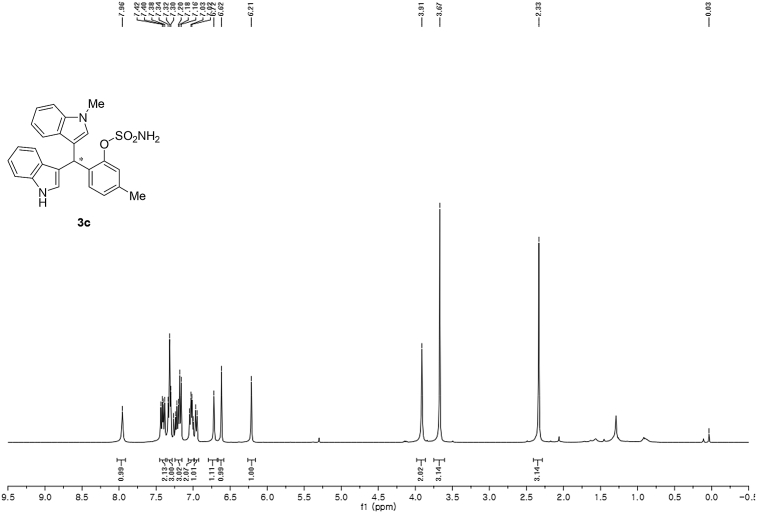

^13^C NMR (100 MHz) in CDCl_3_

**Figure:**
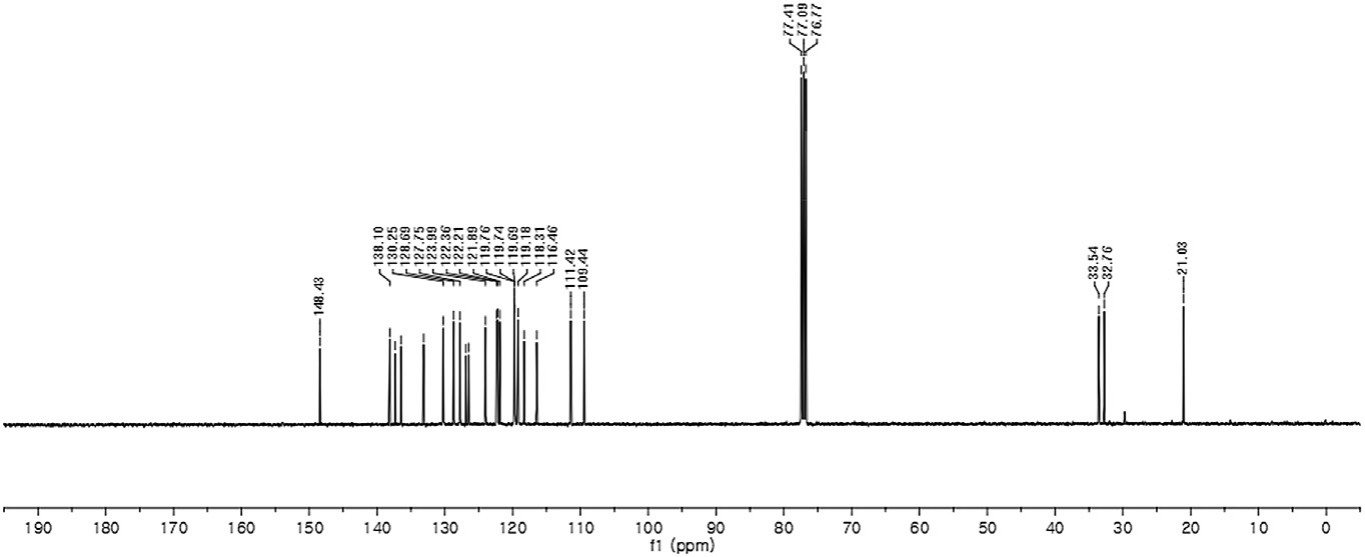

^1^H NMR (400 MHz) in CDCl_3_

**Figure:**
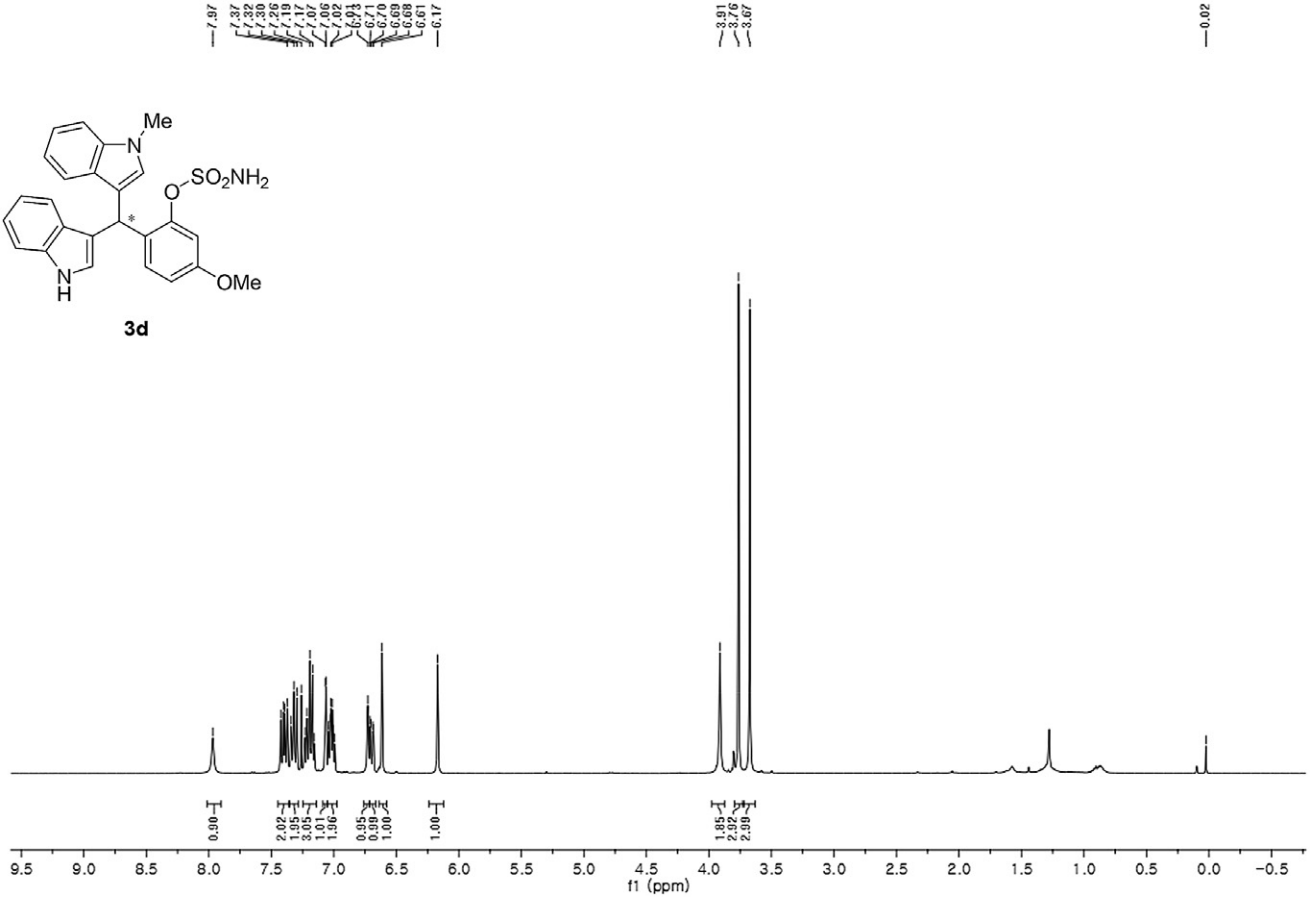

^13^C NMR (100 MHz) in CDCl_3_

**Figure:**
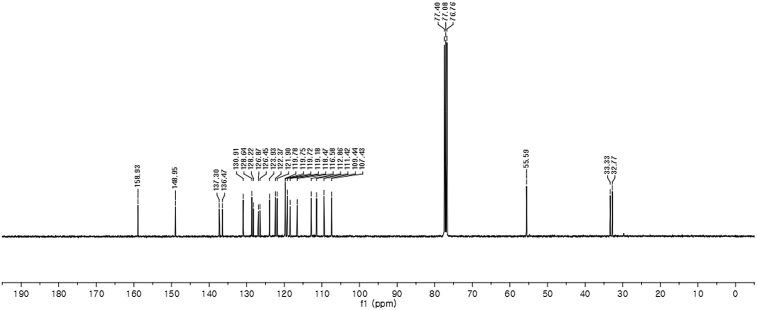

^1^H NMR (400 MHz) in CDCl_3_

**Figure:**
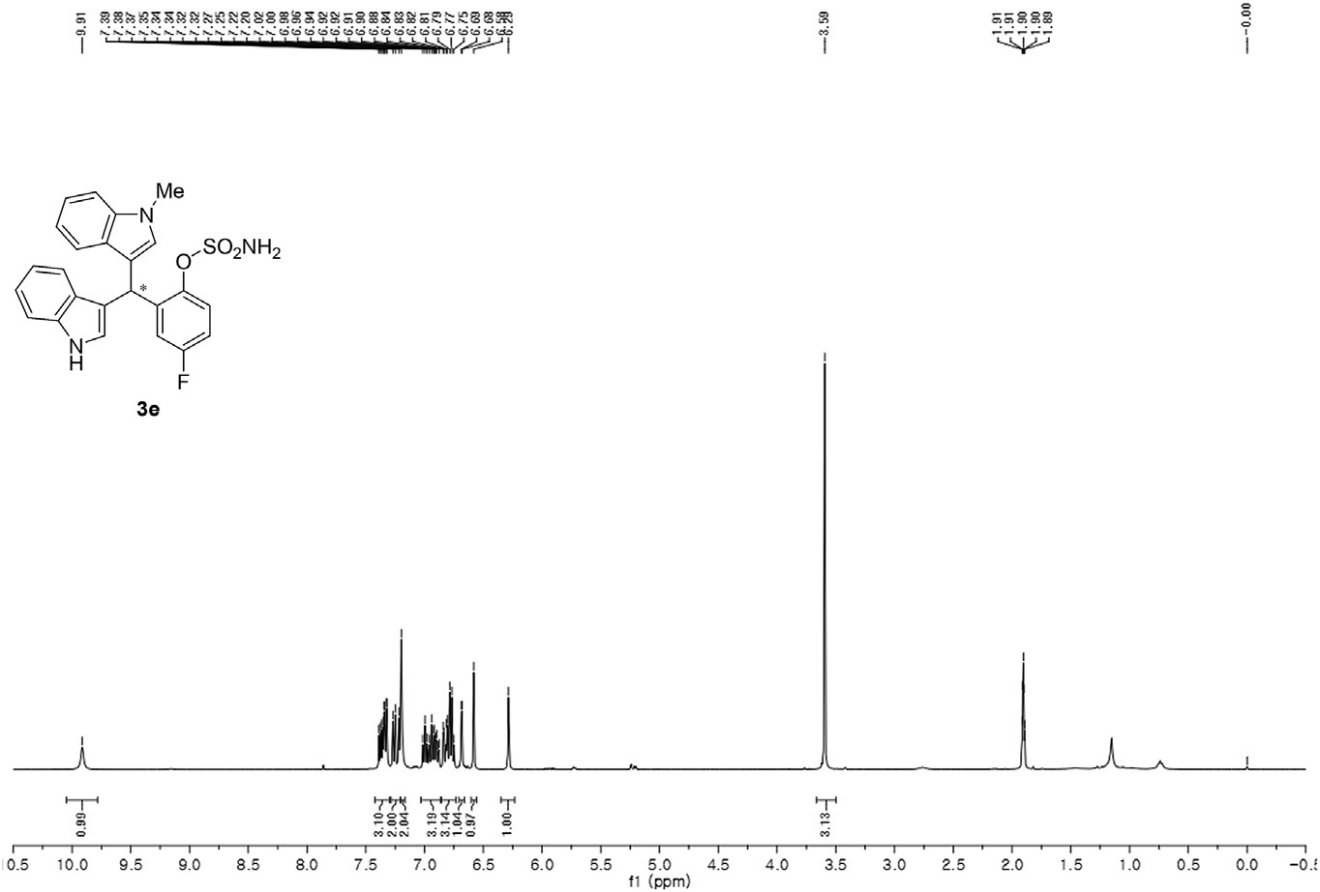

^13^C NMR (100 MHz) in CDCl_3_

**Figure:**
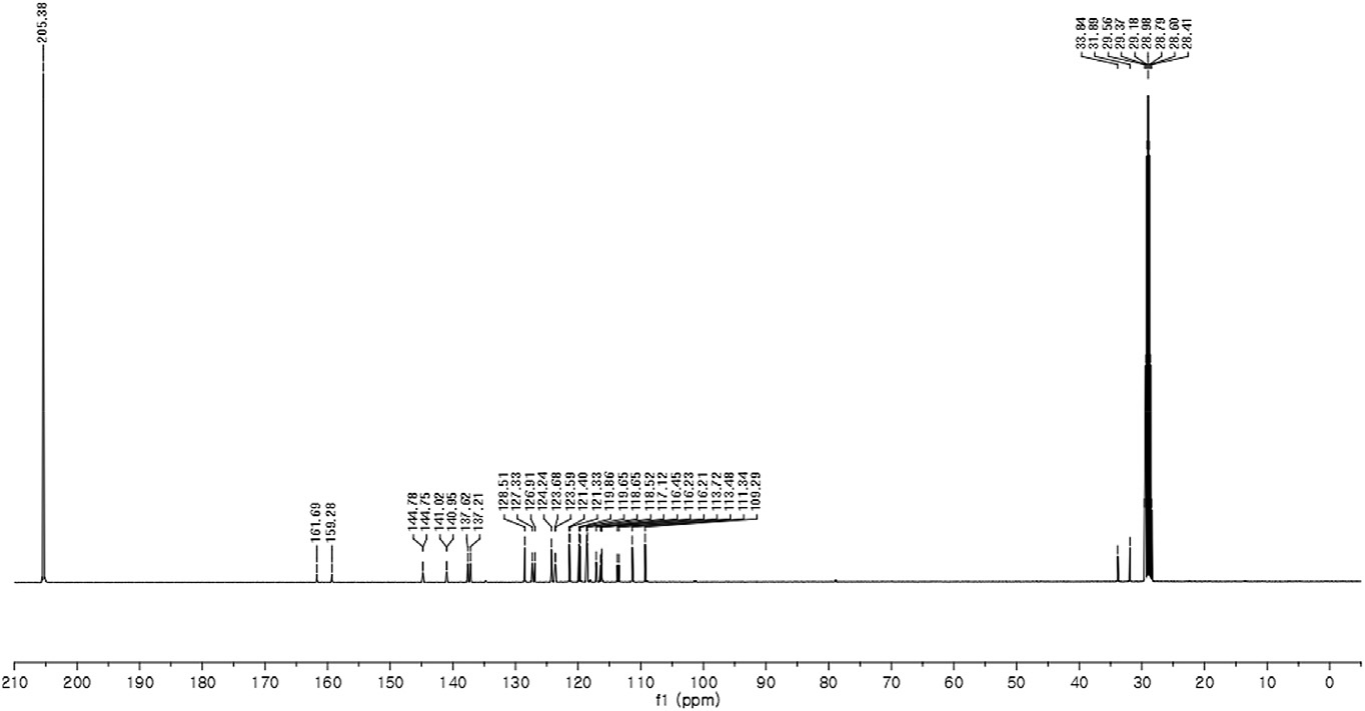

^1^H NMR (400 MHz) in CDCl_3_

**Figure:**
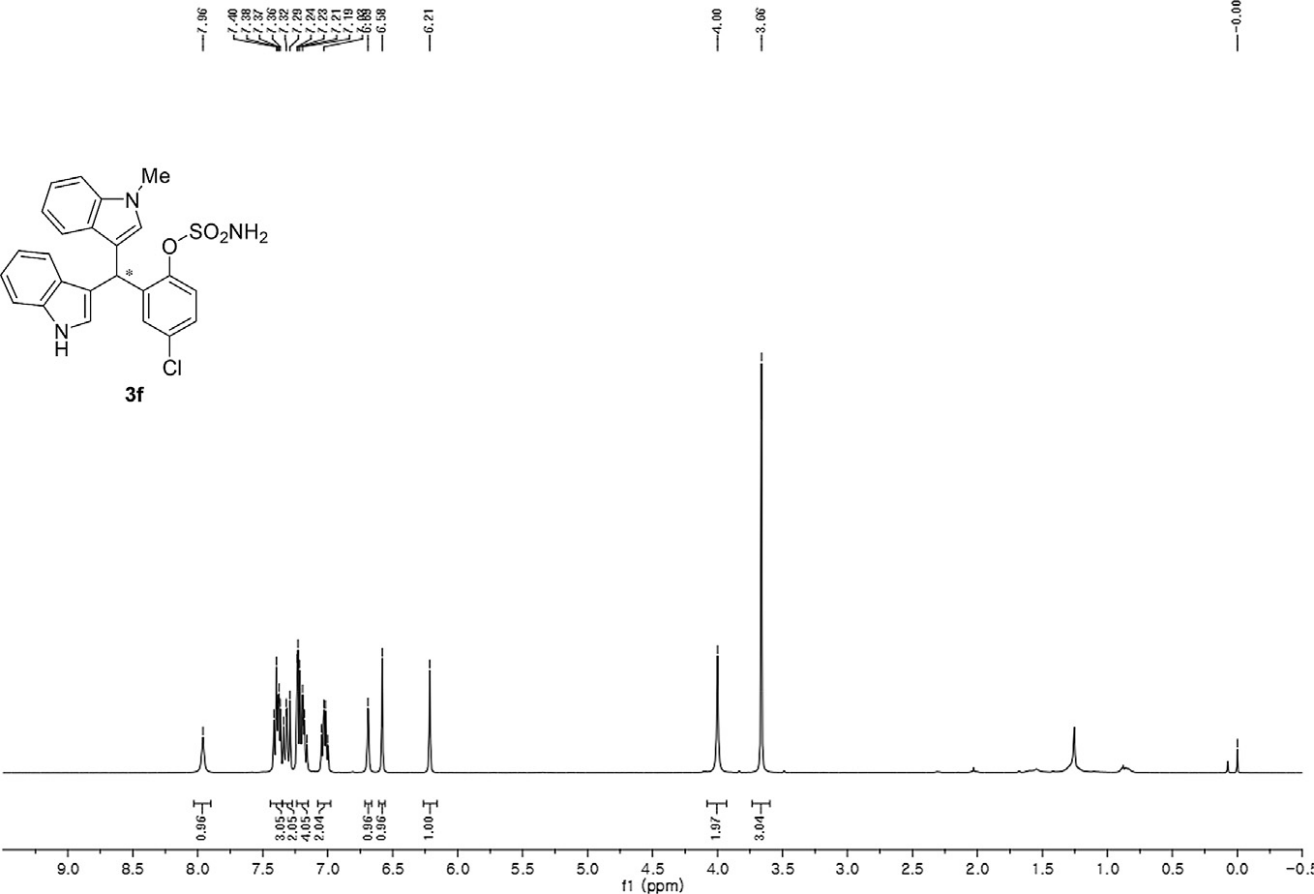

^13^C NMR (100 MHz) in CDCl_3_

**Figure:**
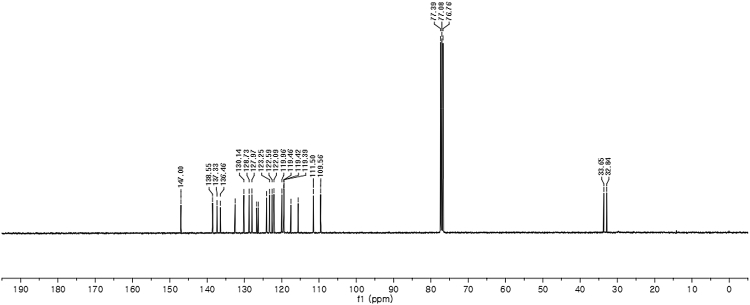

^1^H NMR (400 MHz) in CDCl_3_

**Figure:**
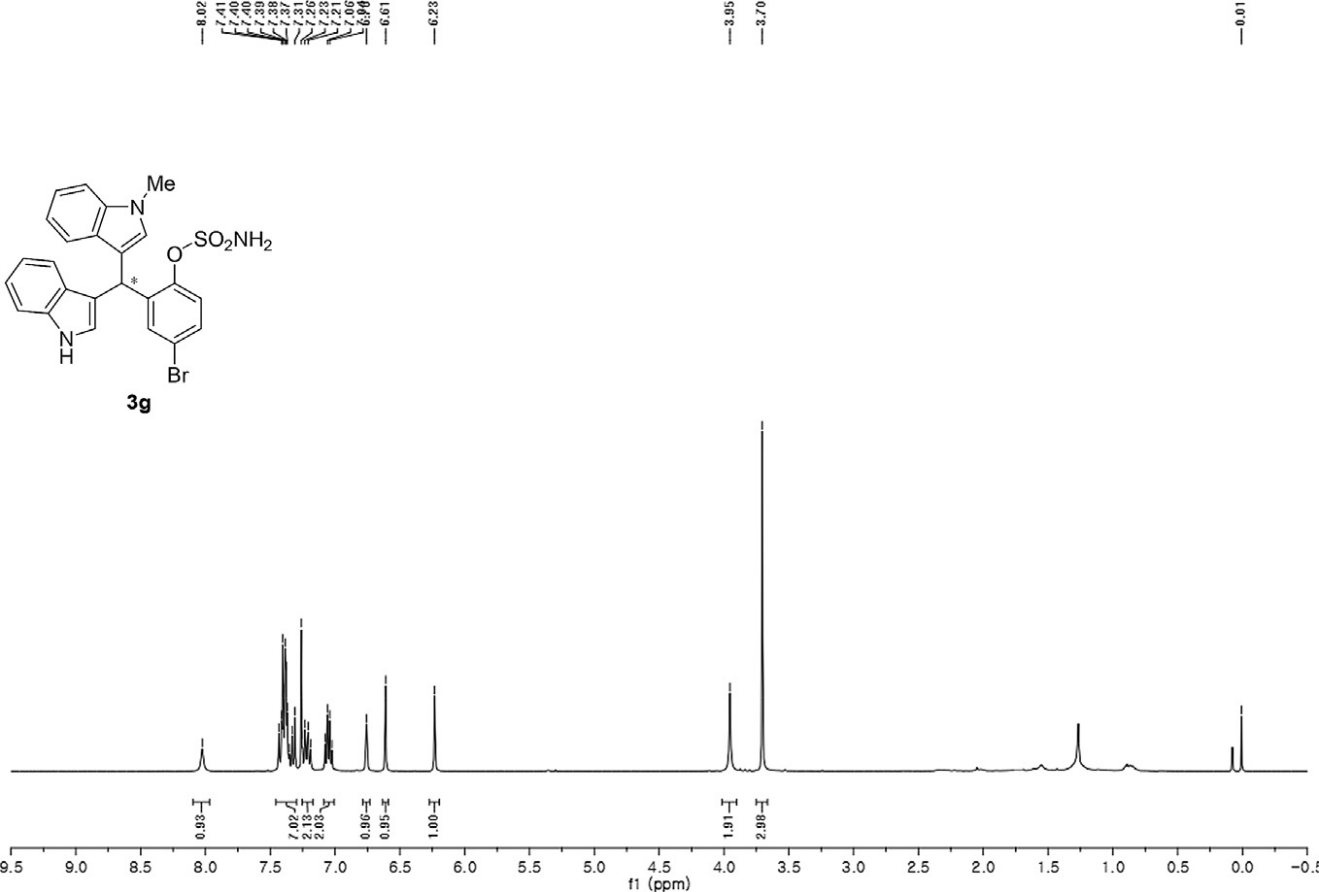

^13^C NMR (100 MHz) in CDCl_3_

**Figure:**
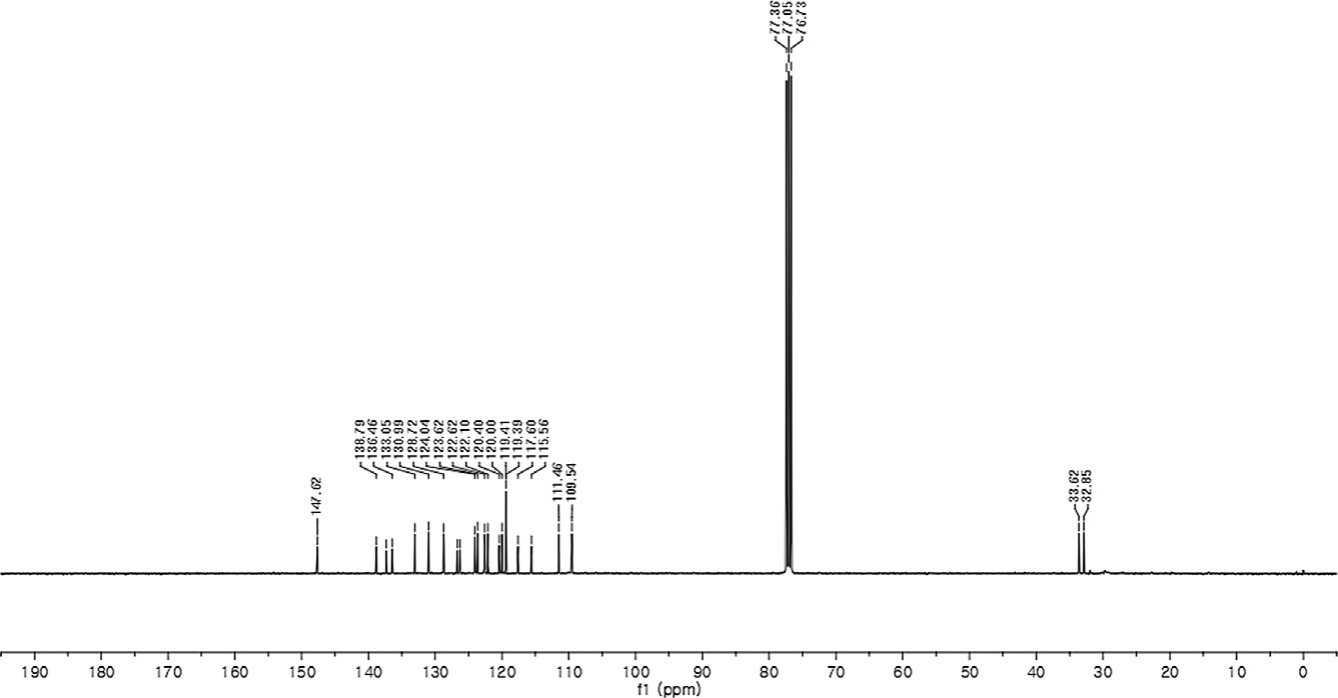

^1^H NMR (400 MHz) in CDCl_3_

**Figure:**
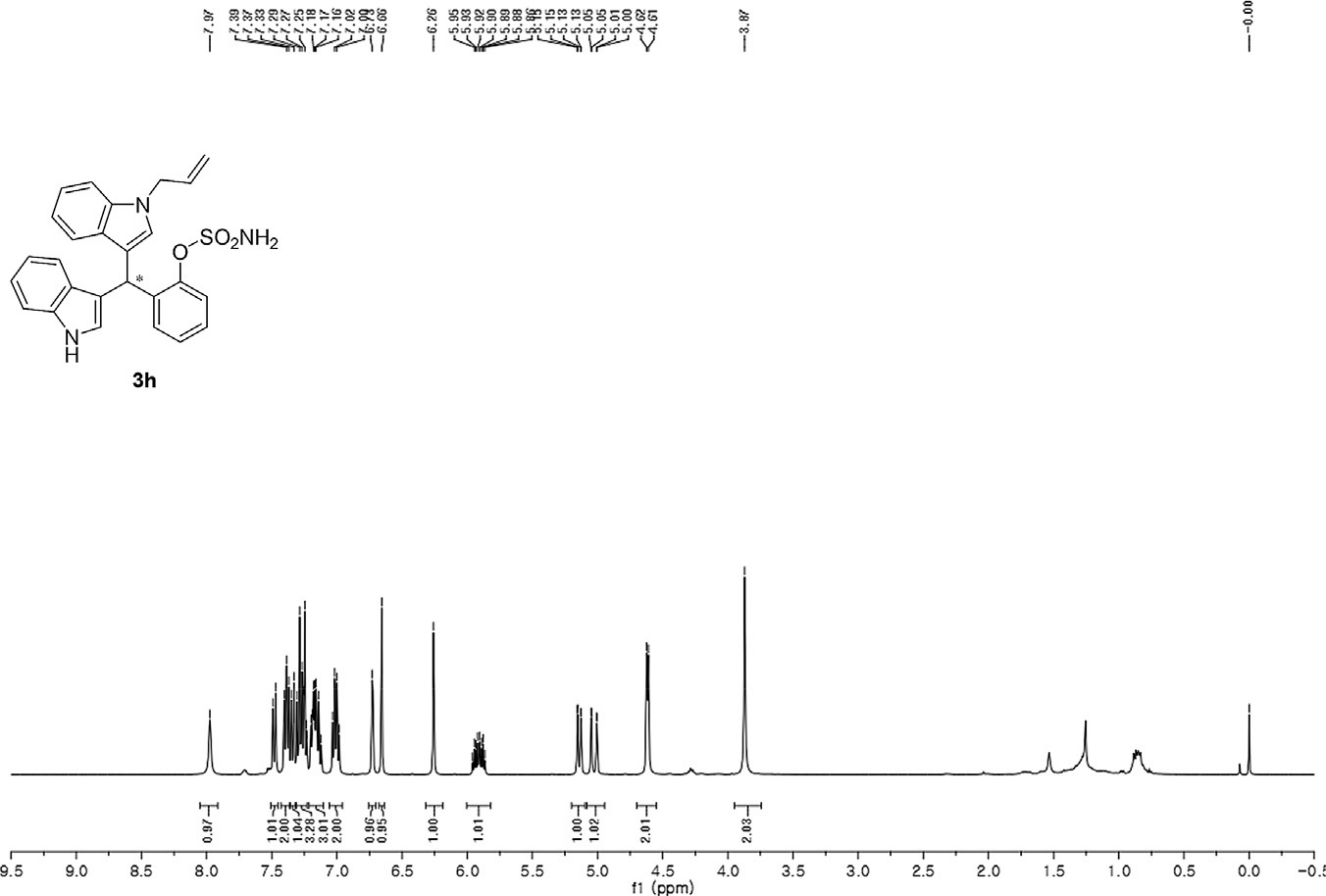

^13^C NMR (100 MHz) in CDCl_3_

**Figure:**
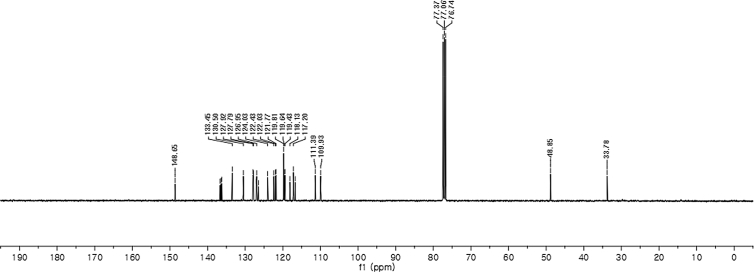

^1^H NMR (400 MHz) in CDCl_3_

**Figure:**
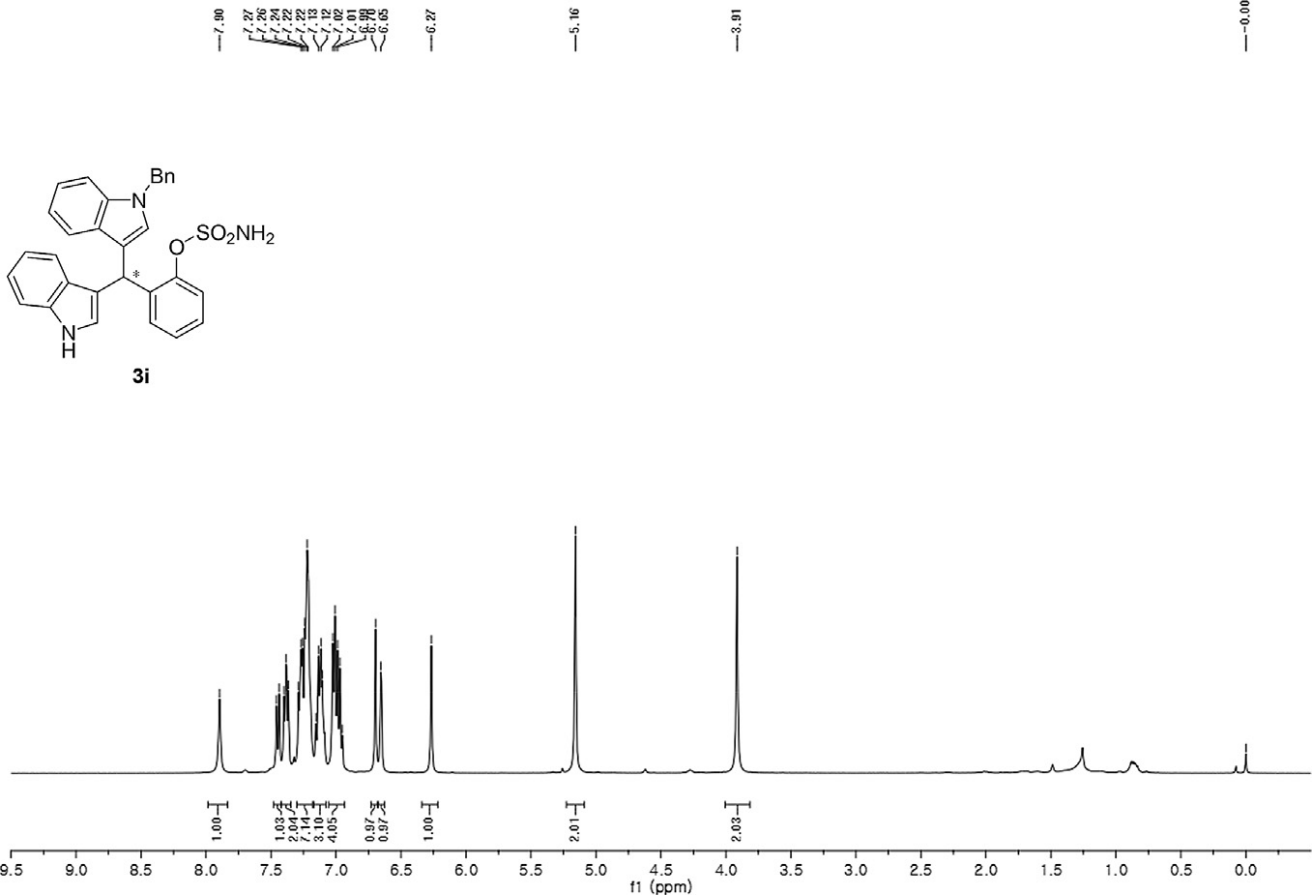

^13^C NMR (100 MHz) in CDCl_3_

**Figure:**
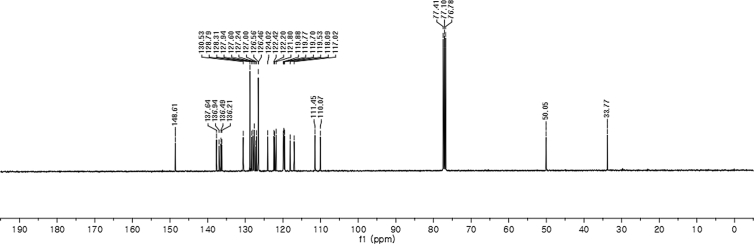

^1^H NMR (400 MHz) in CDCl_3_

**Figure:**
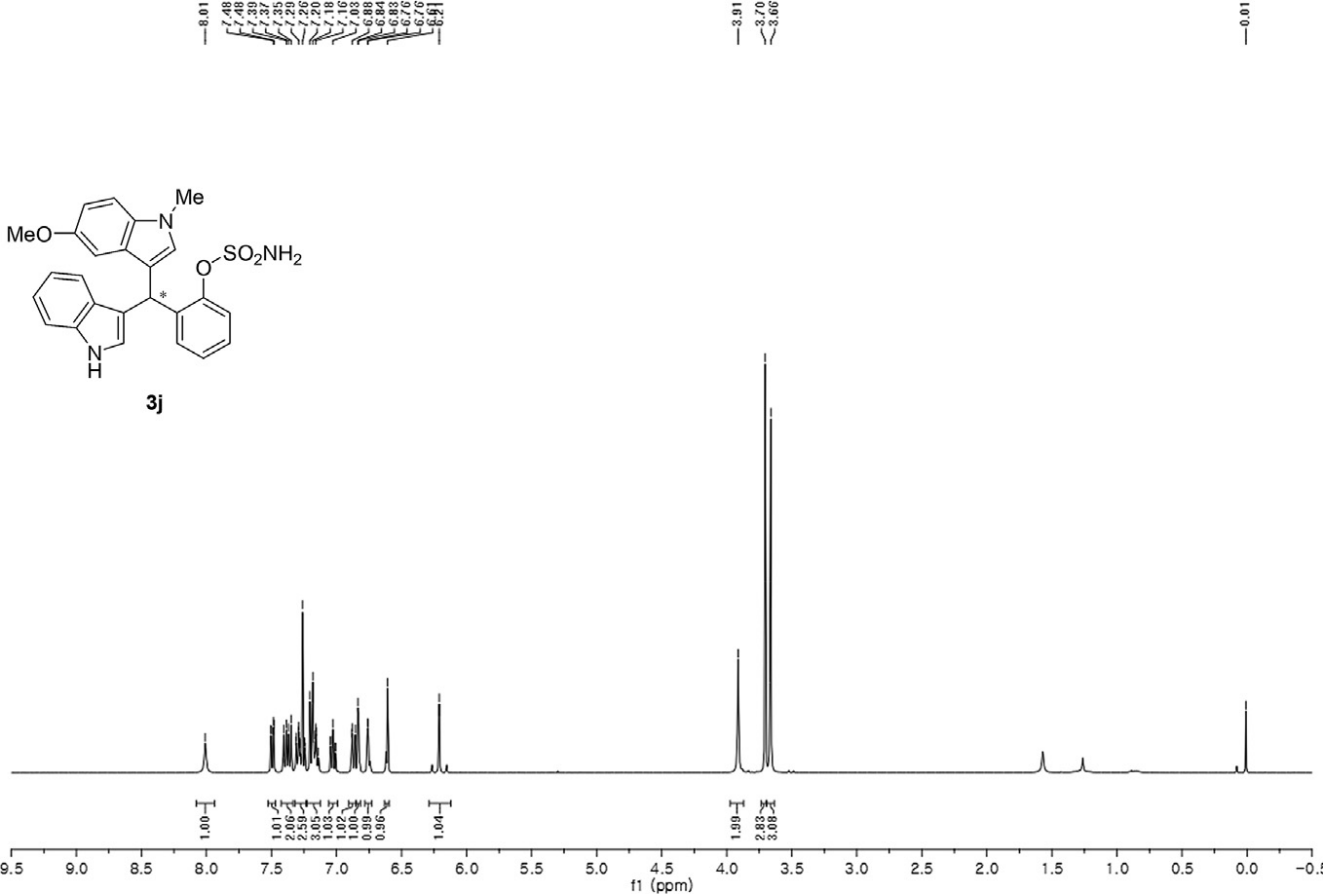

^13^C NMR (100 MHz) in CDCl_3_

**Figure:**
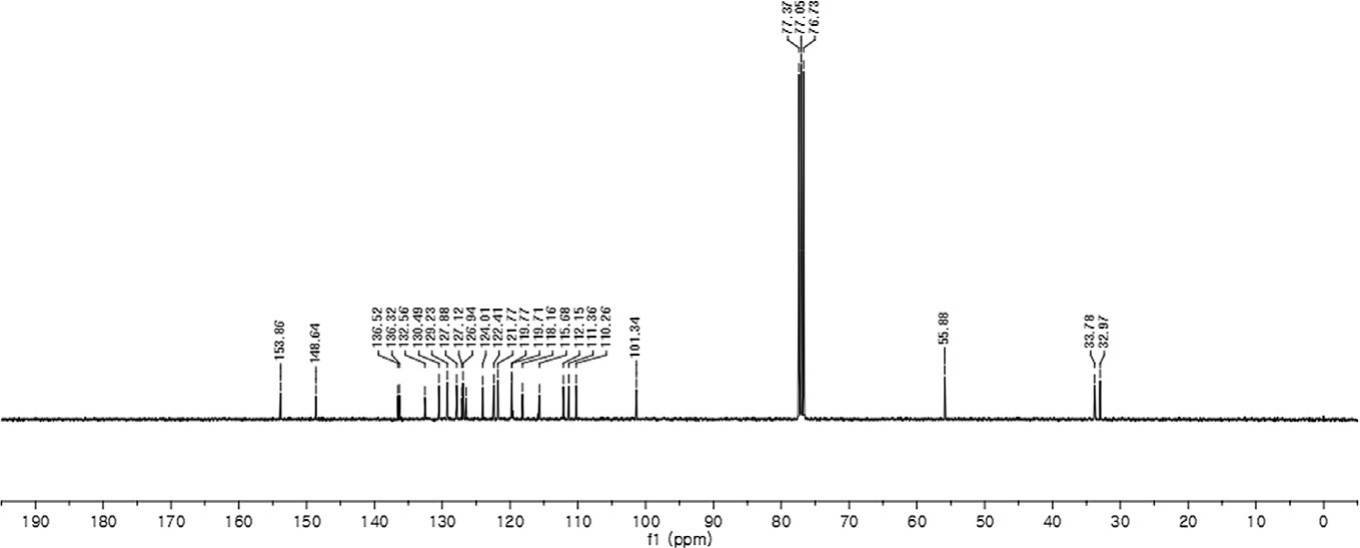

^1^H NMR (400 MHz) in CDCl_3_

**Figure:**
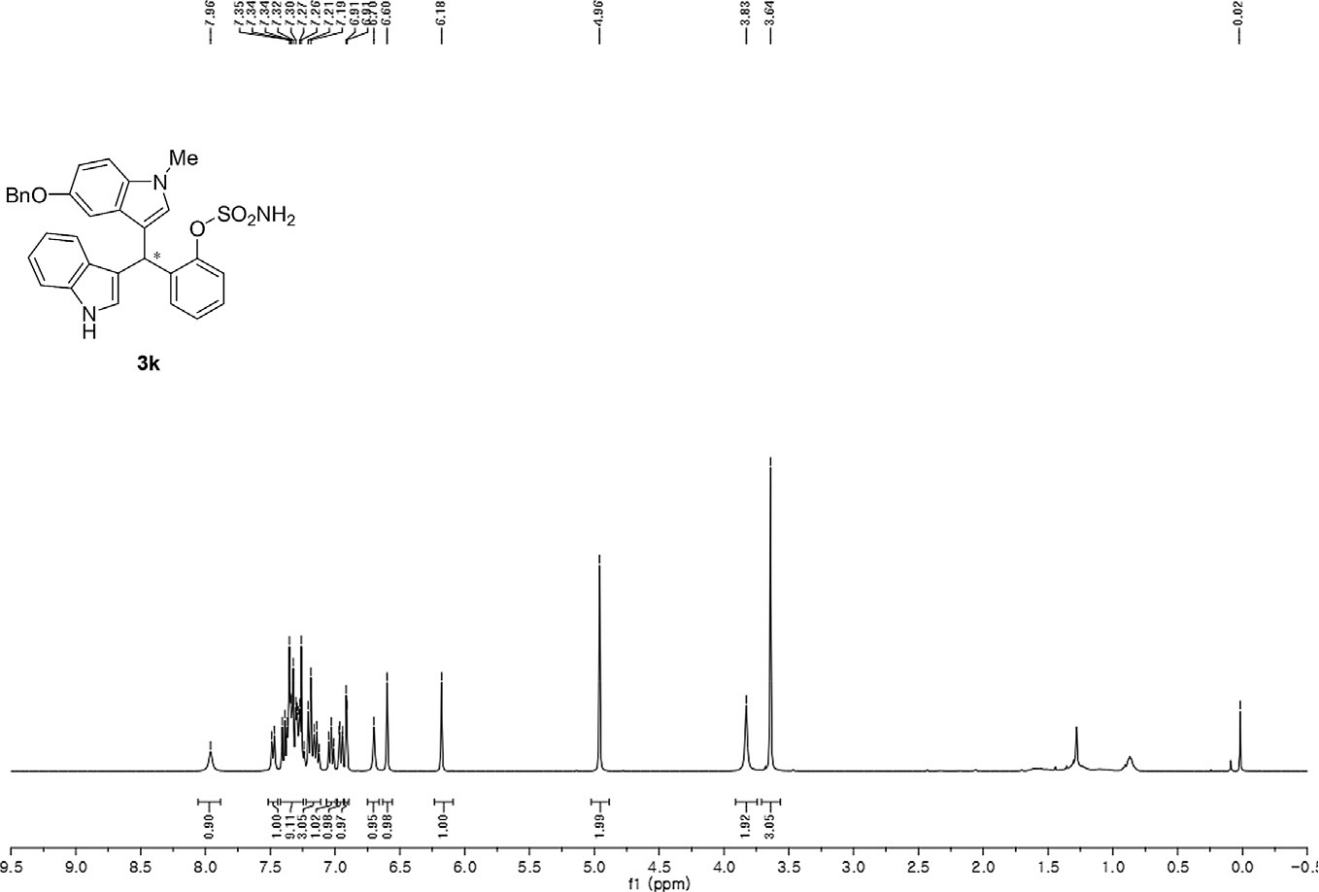

^13^C NMR (100 MHz) in CDCl_3_

**Figure:**
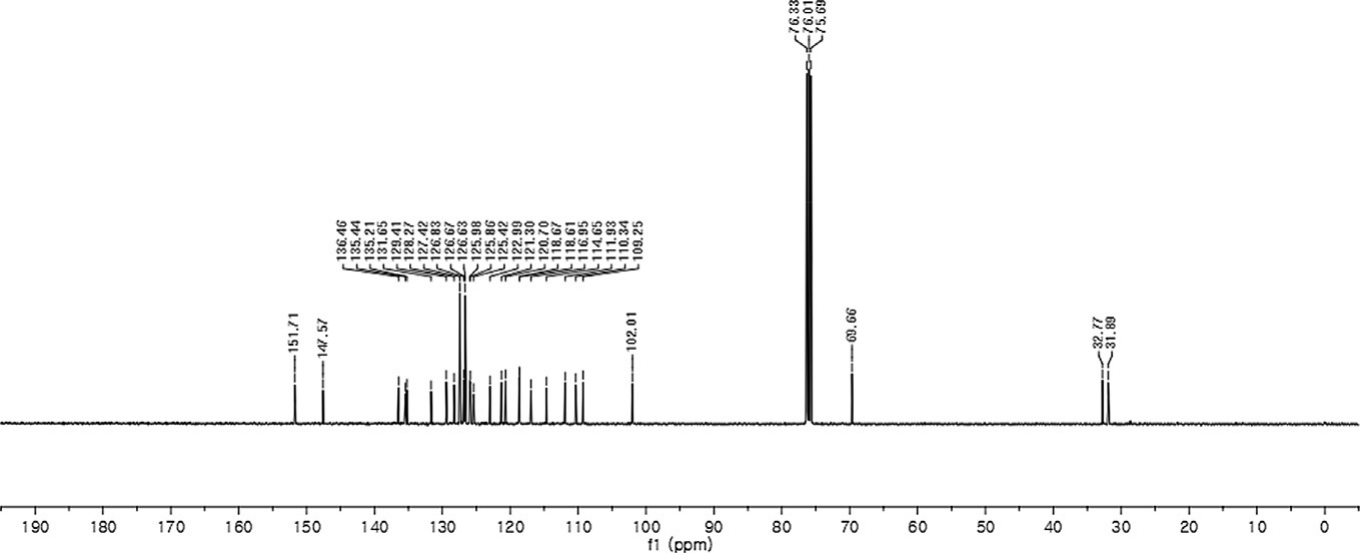

^1^H NMR (400 MHz) in CDCl_3_

**Figure:**
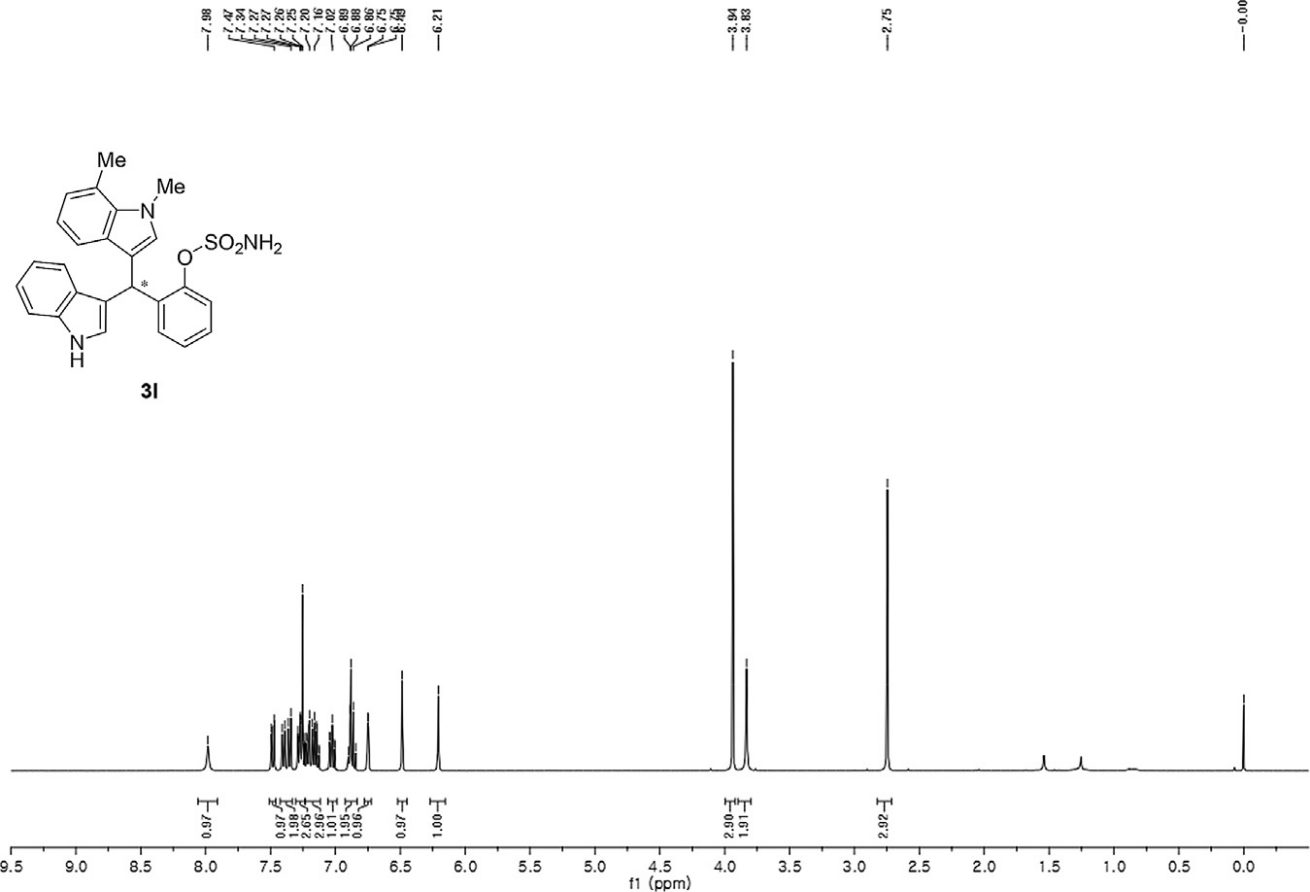

^13^C NMR (100 MHz) in CDCl_3_

**Figure:**
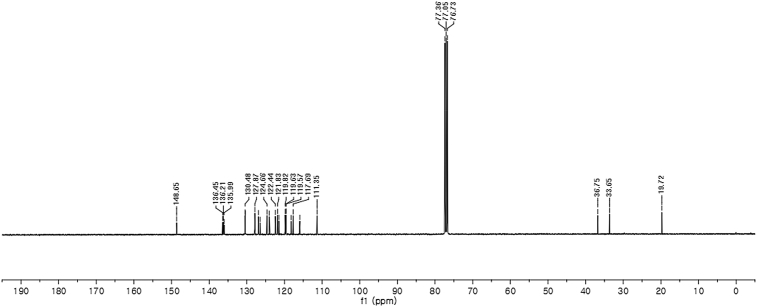

^1^H NMR (400 MHz) in CDCl_3_

**Figure:**
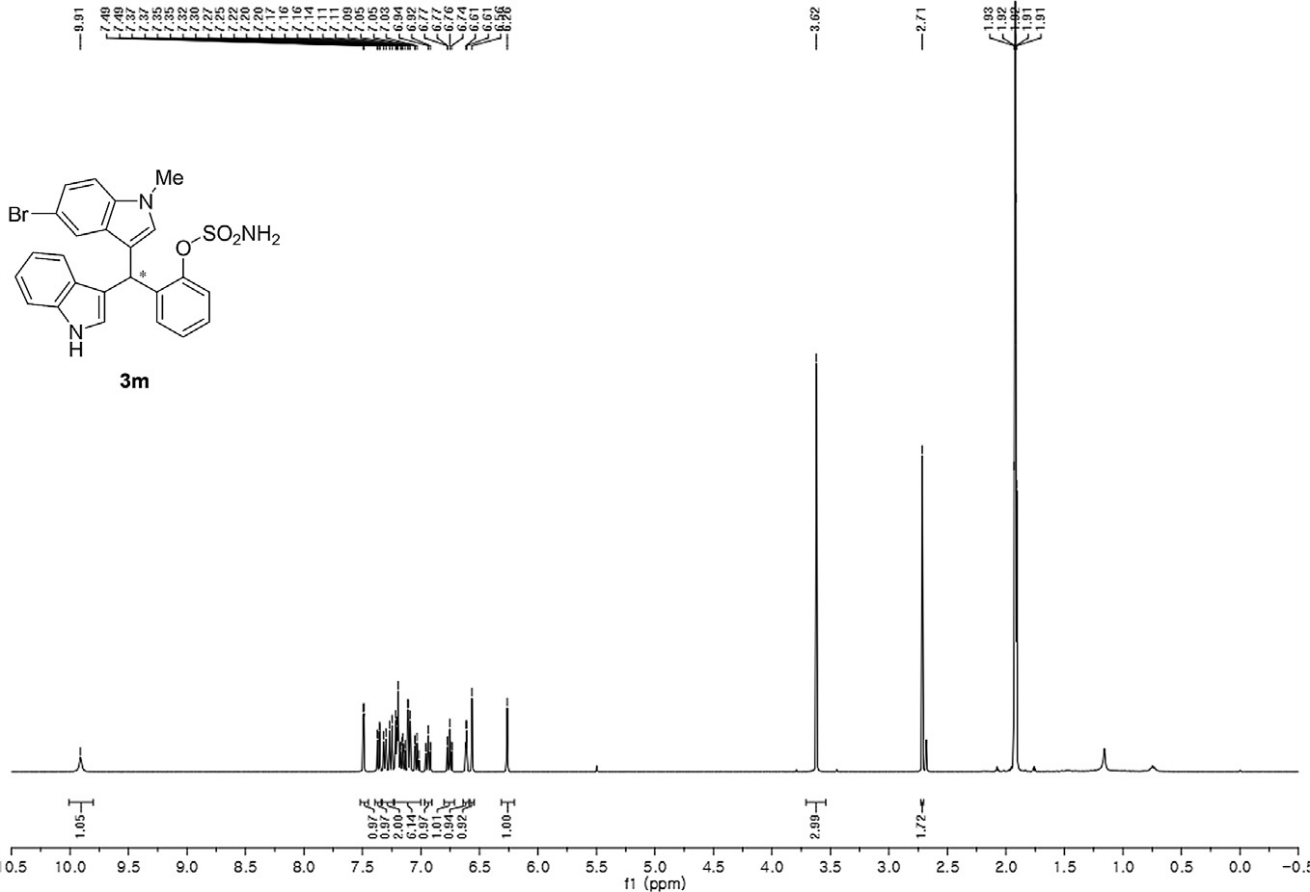

^13^C NMR (100 MHz) in CDCl_3_

**Figure:**
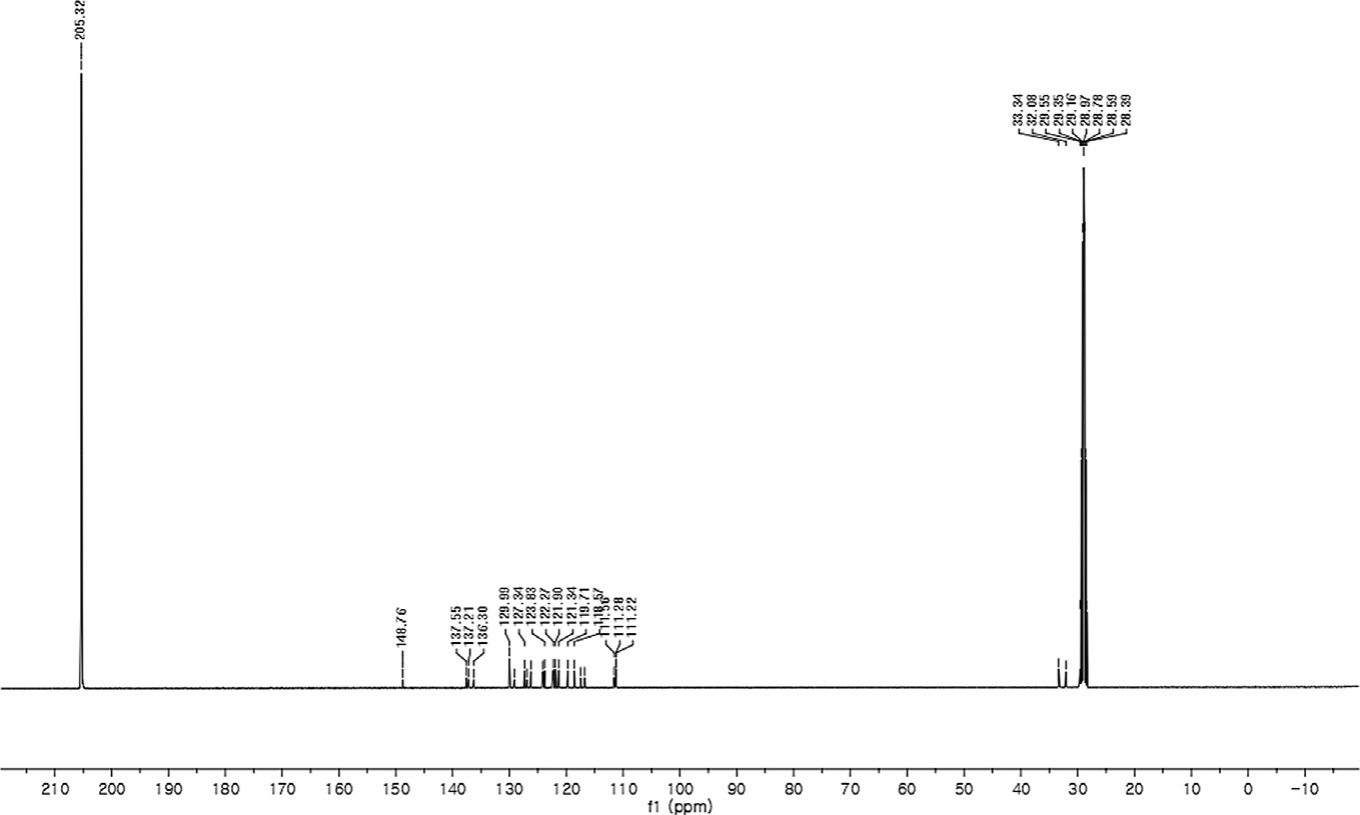

^1^H NMR (400 MHz) in CDCl_3_

**Figure:**
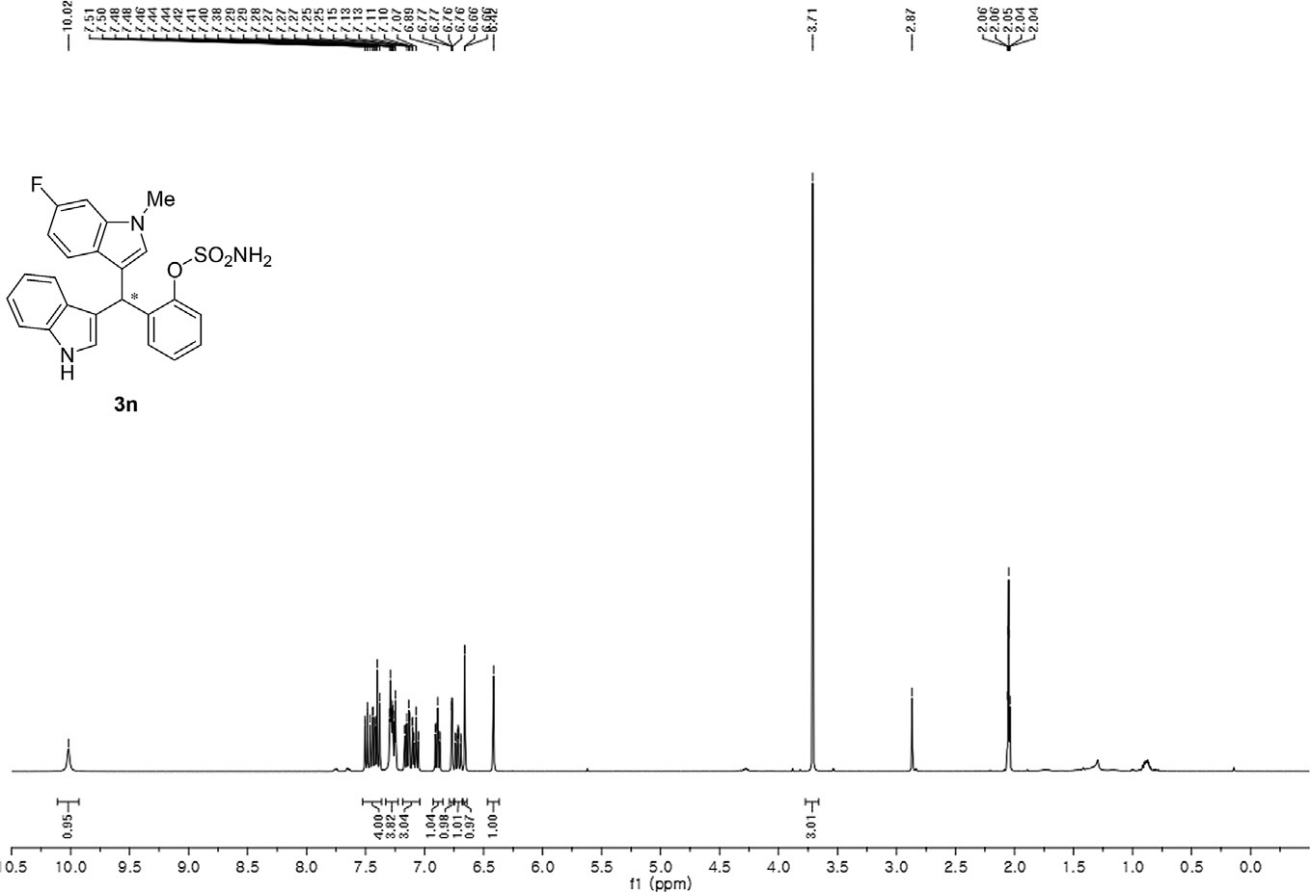

^13^C NMR (100 MHz) in CDCl_3_

**Figure:**
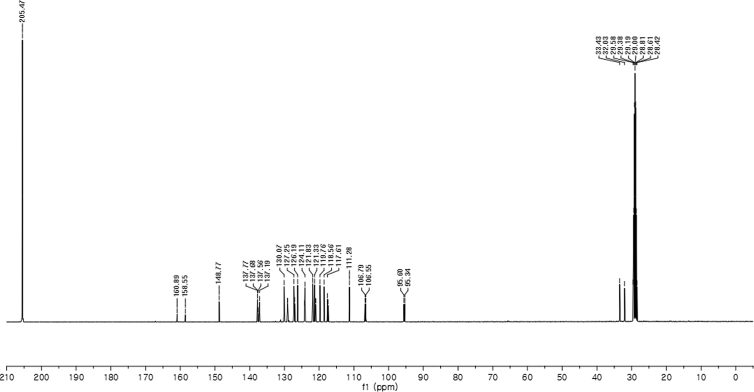

^1^H NMR (400 MHz) in CDCl_3_

**Figure:**
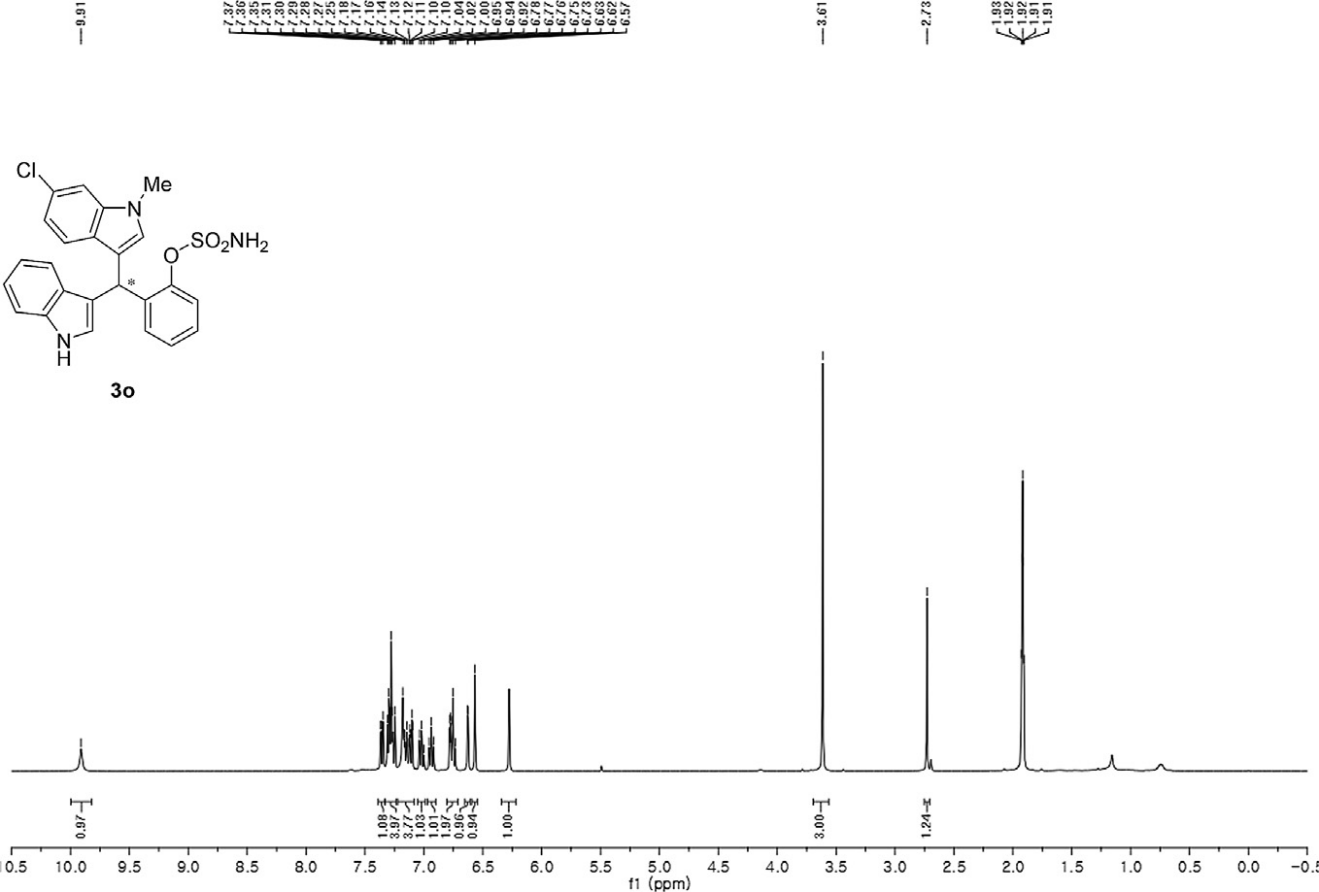

^13^C NMR (100 MHz) in CDCl_3_

**Figure:**
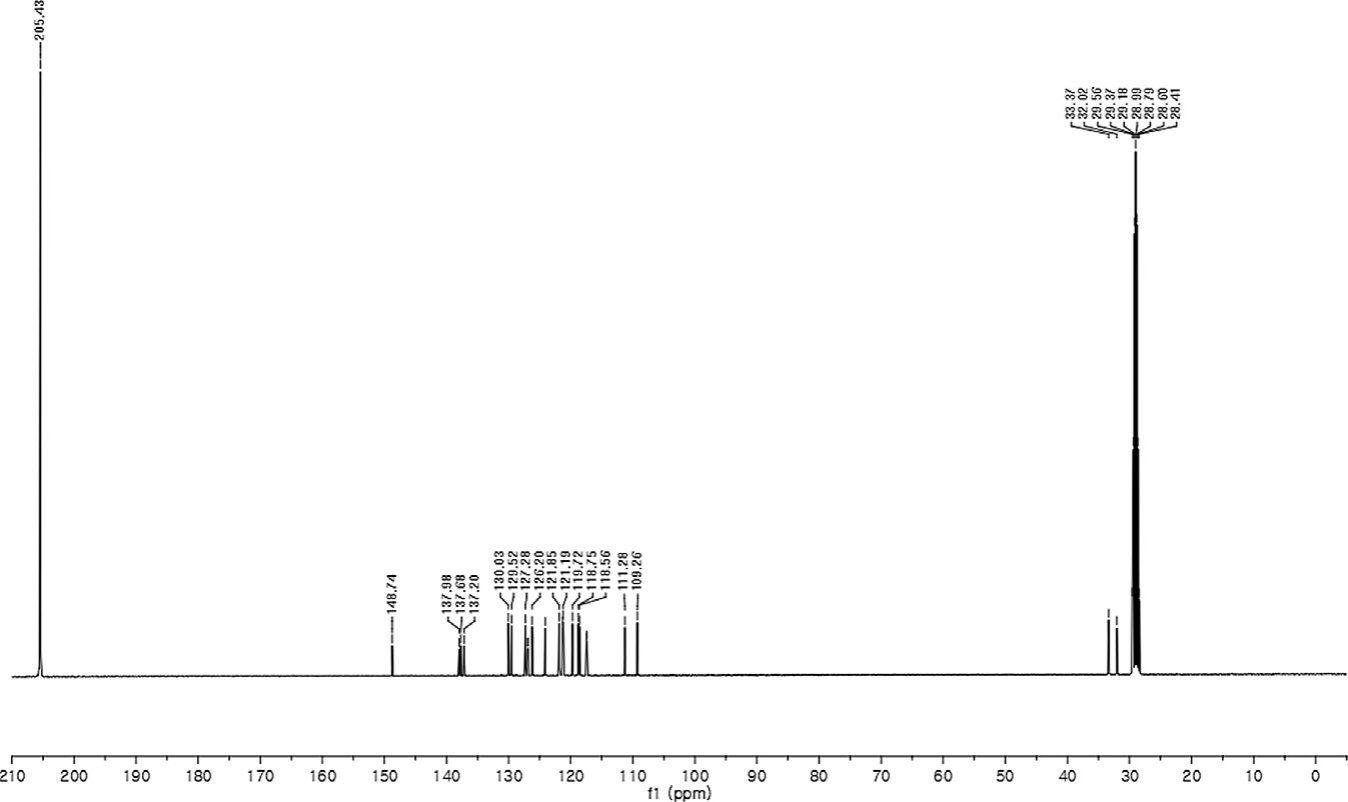

^1^H NMR (400 MHz) in CDCl_3_

**Figure:**
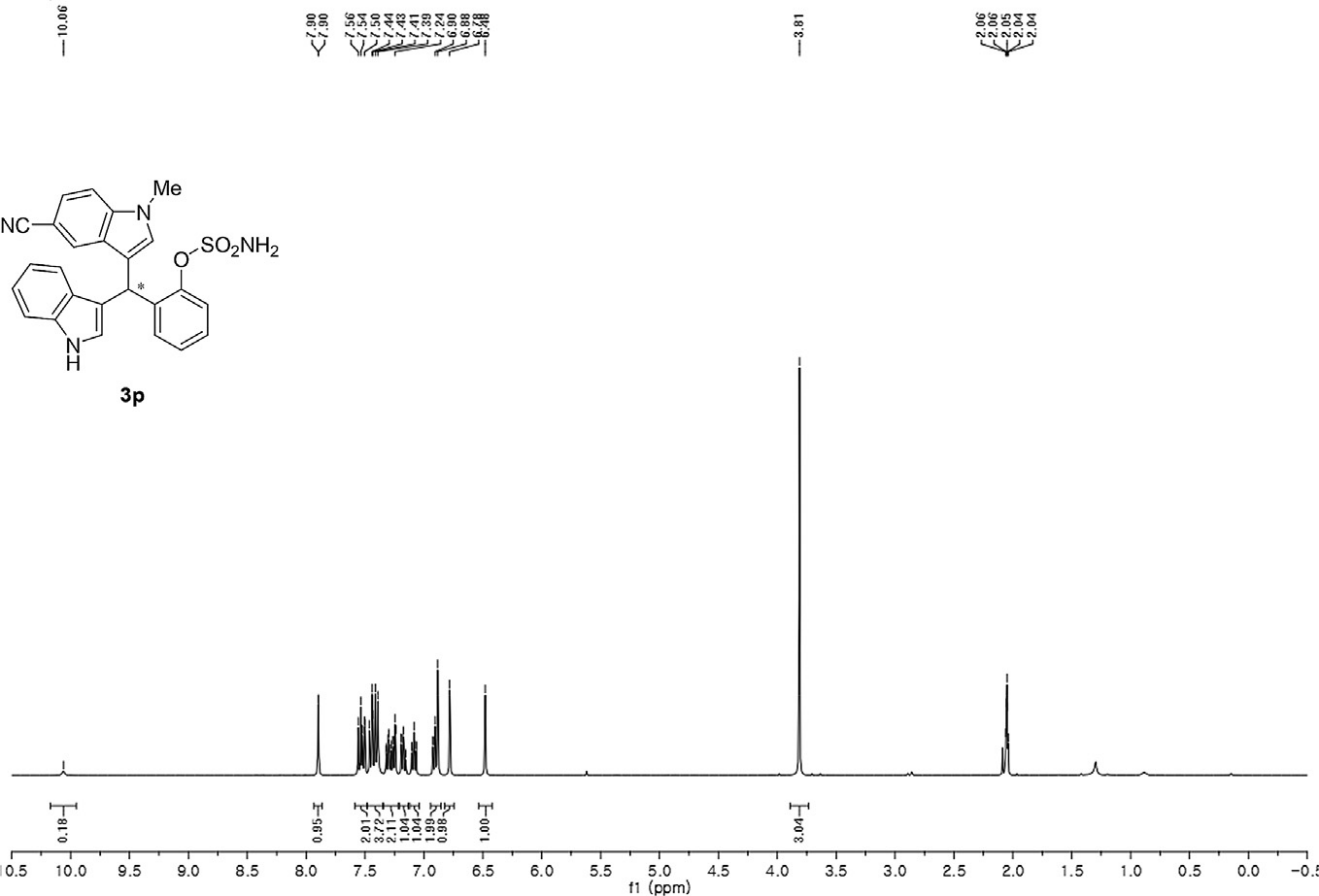

^13^C NMR (100 MHz) in CDCl_3_

**Figure:**
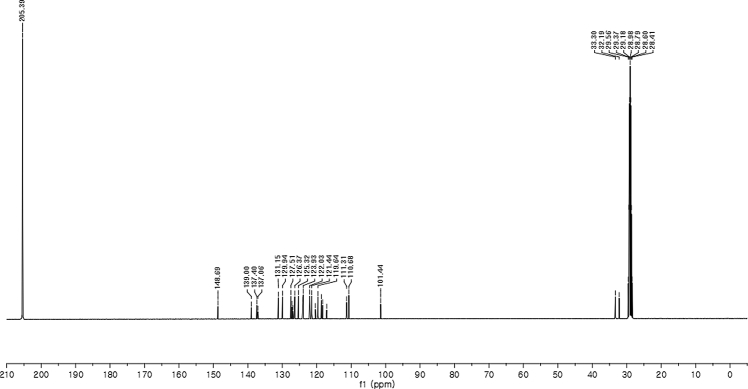

^1^H NMR (400 MHz) in CDCl_3_

**Figure:**
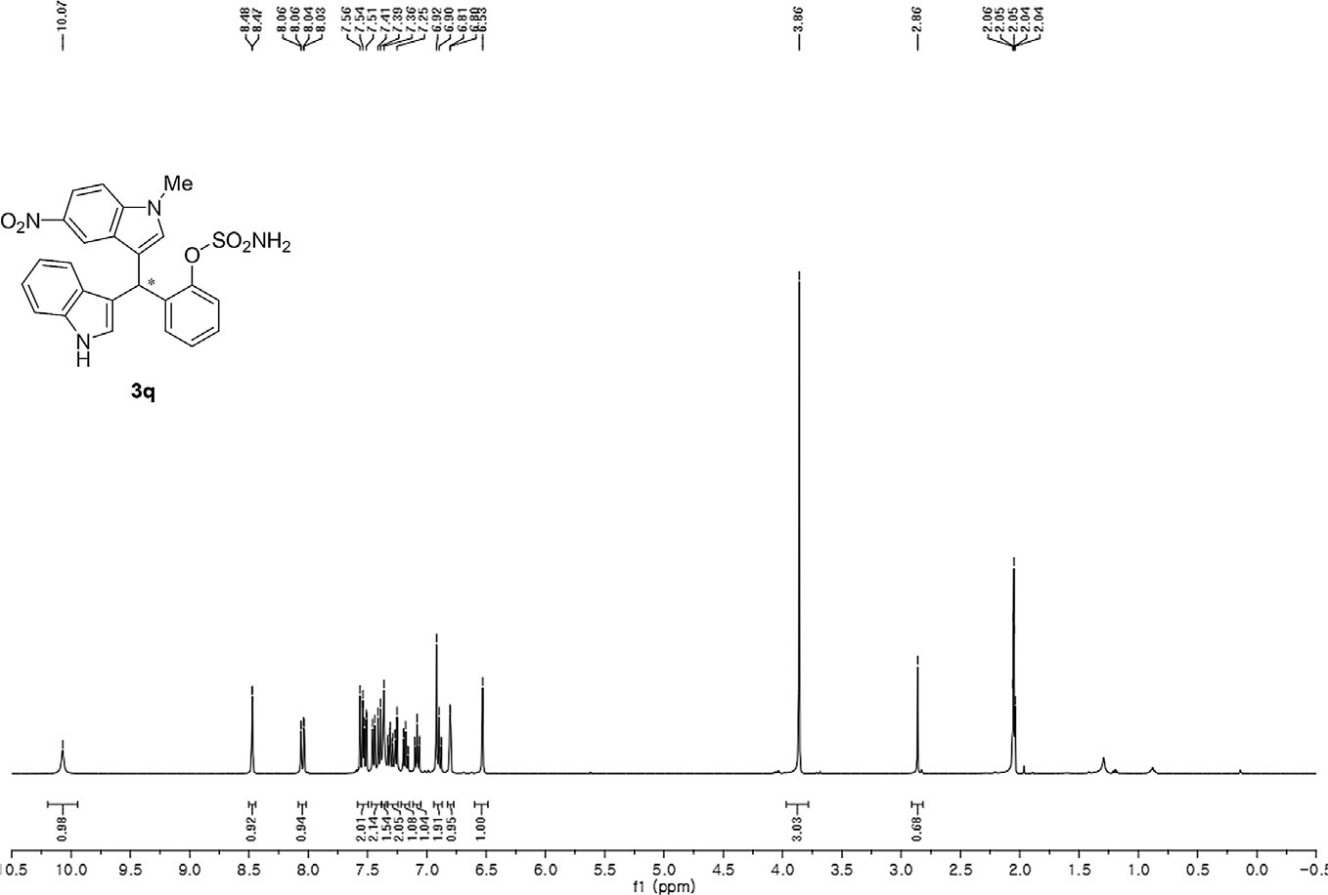

^13^C NMR (100 MHz) in CDCl_3_

**Figure:**
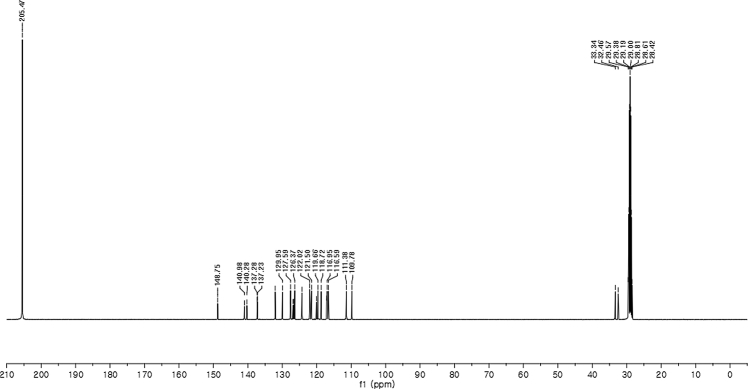

^1^H NMR (400 MHz) in CDCl_3_

**Figure:**
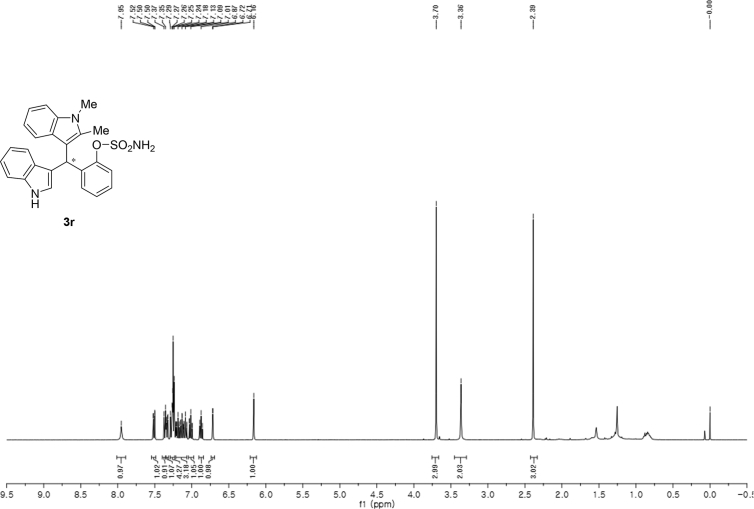

^13^C NMR (100 MHz) in CDCl_3_

**Figure:**
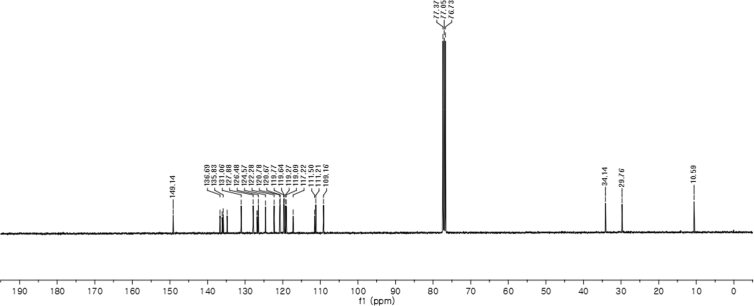

### 2.3 HPLC analysis

**Figure:**
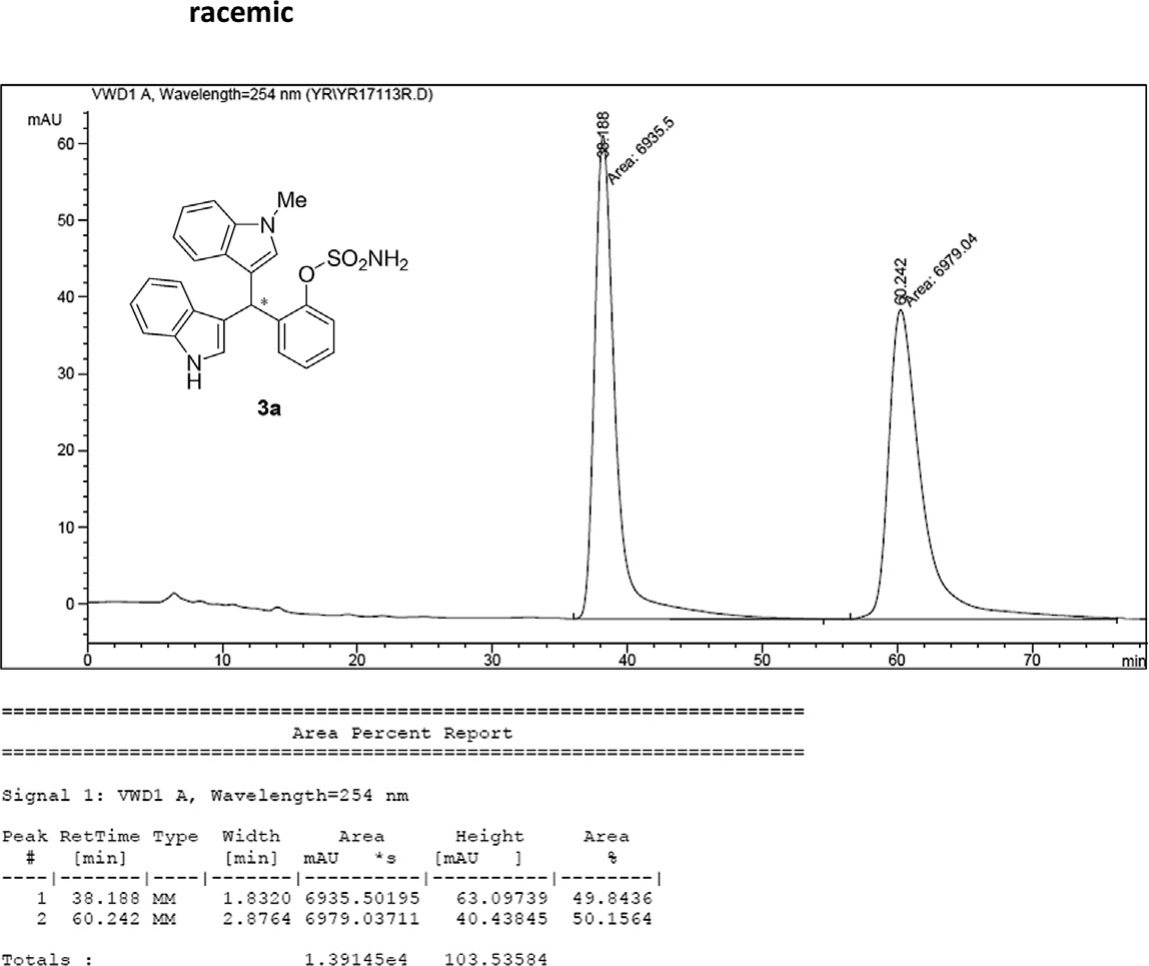

**Figure:**
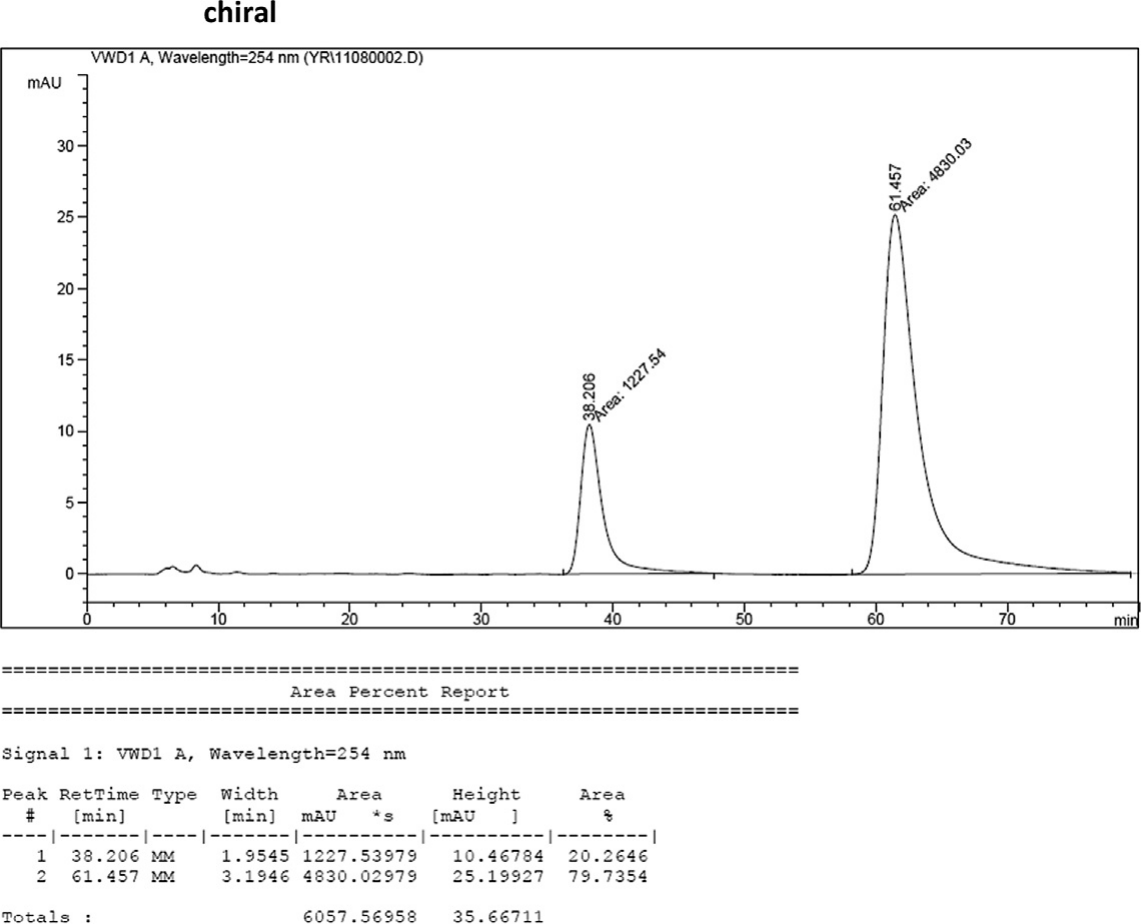

**Figure:**
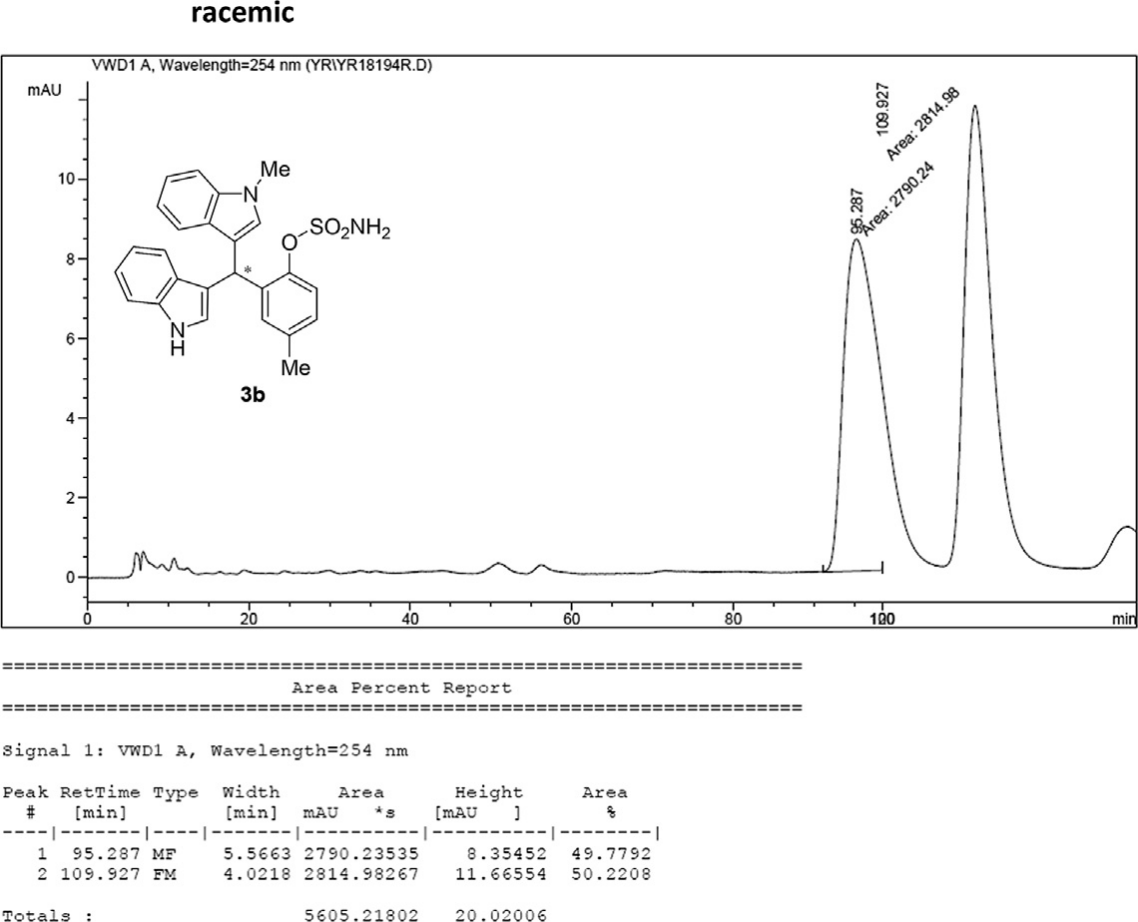

**Figure:**
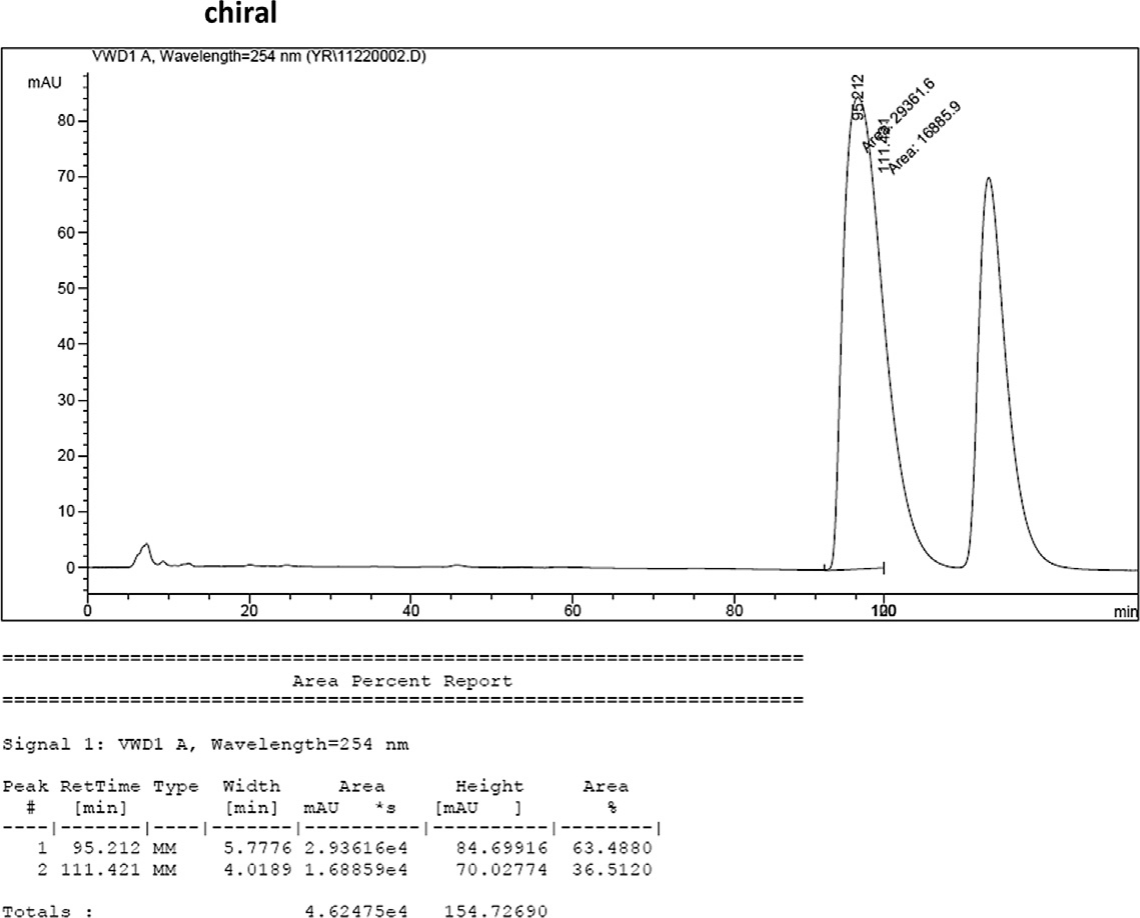

**Figure:**
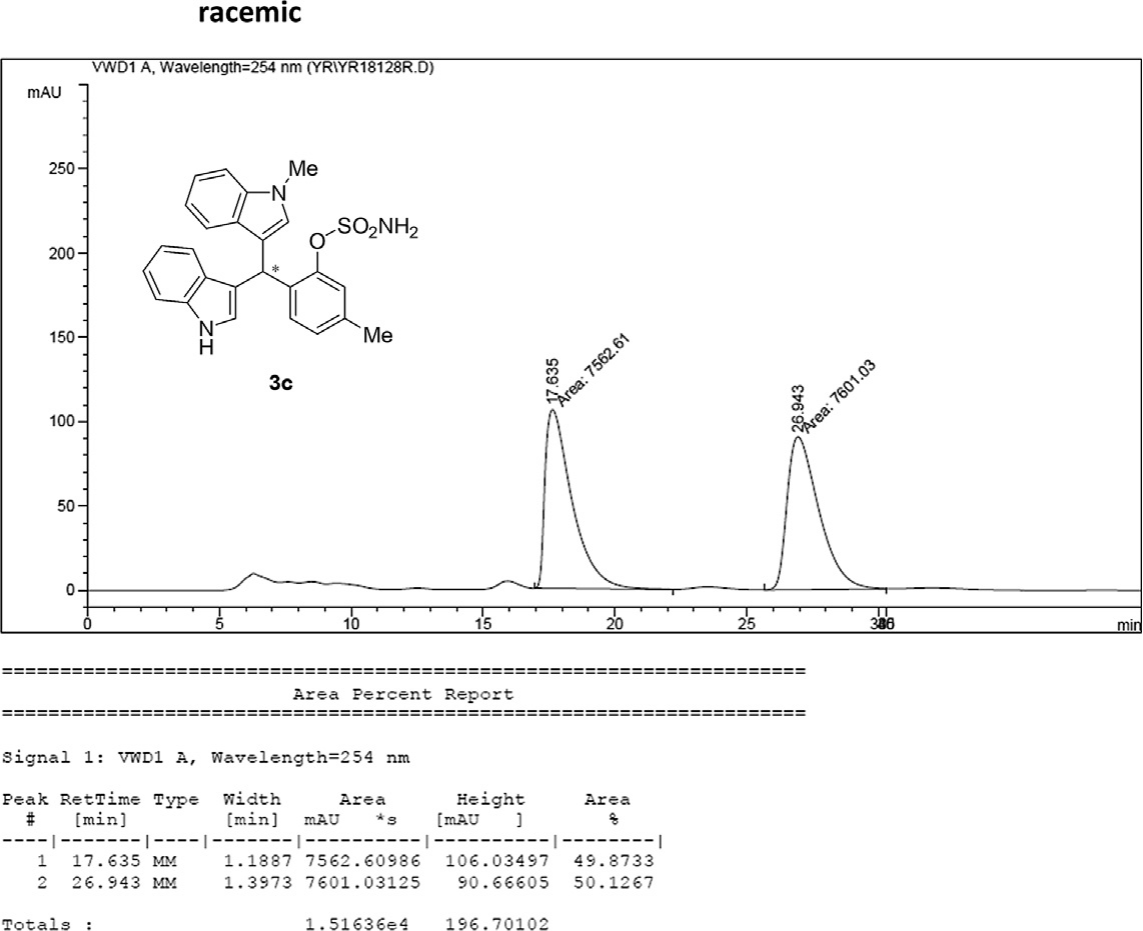

**Figure:**
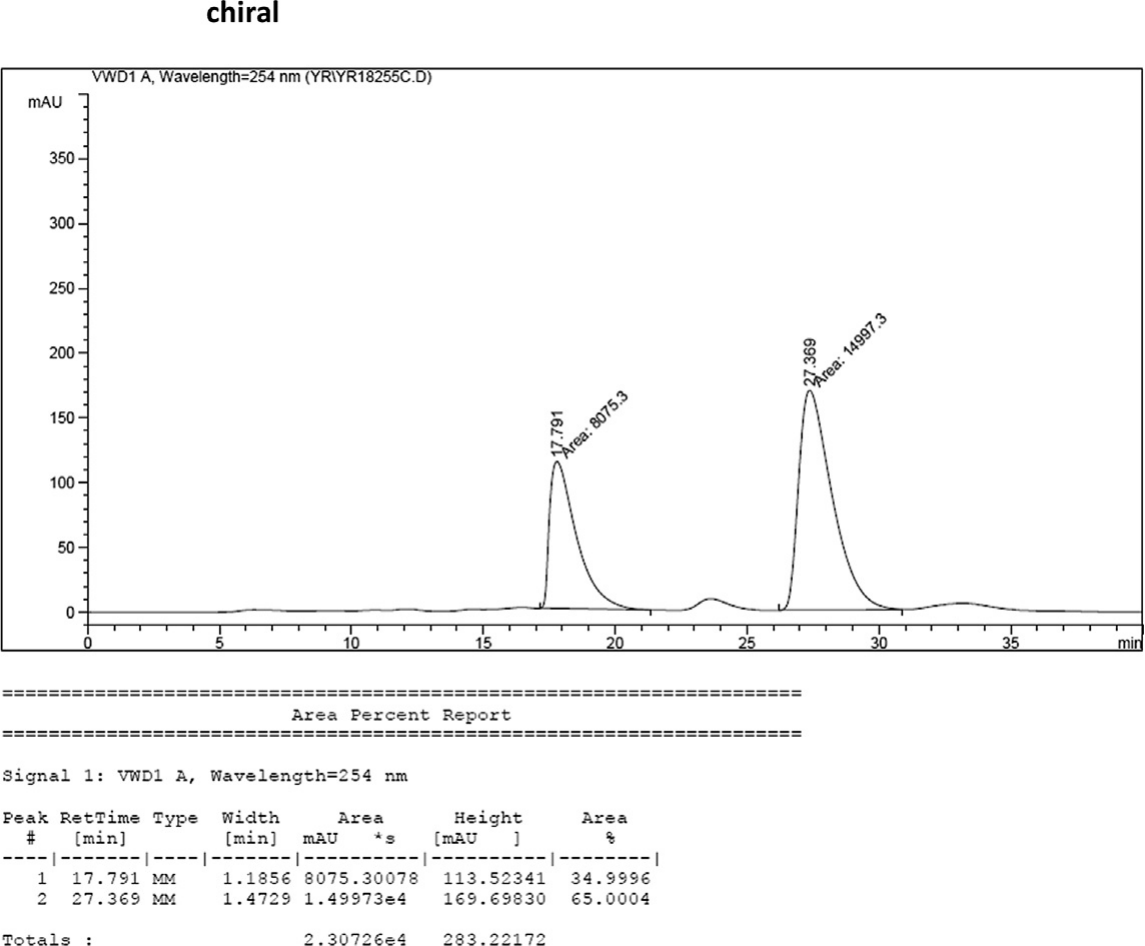

**Figure:**
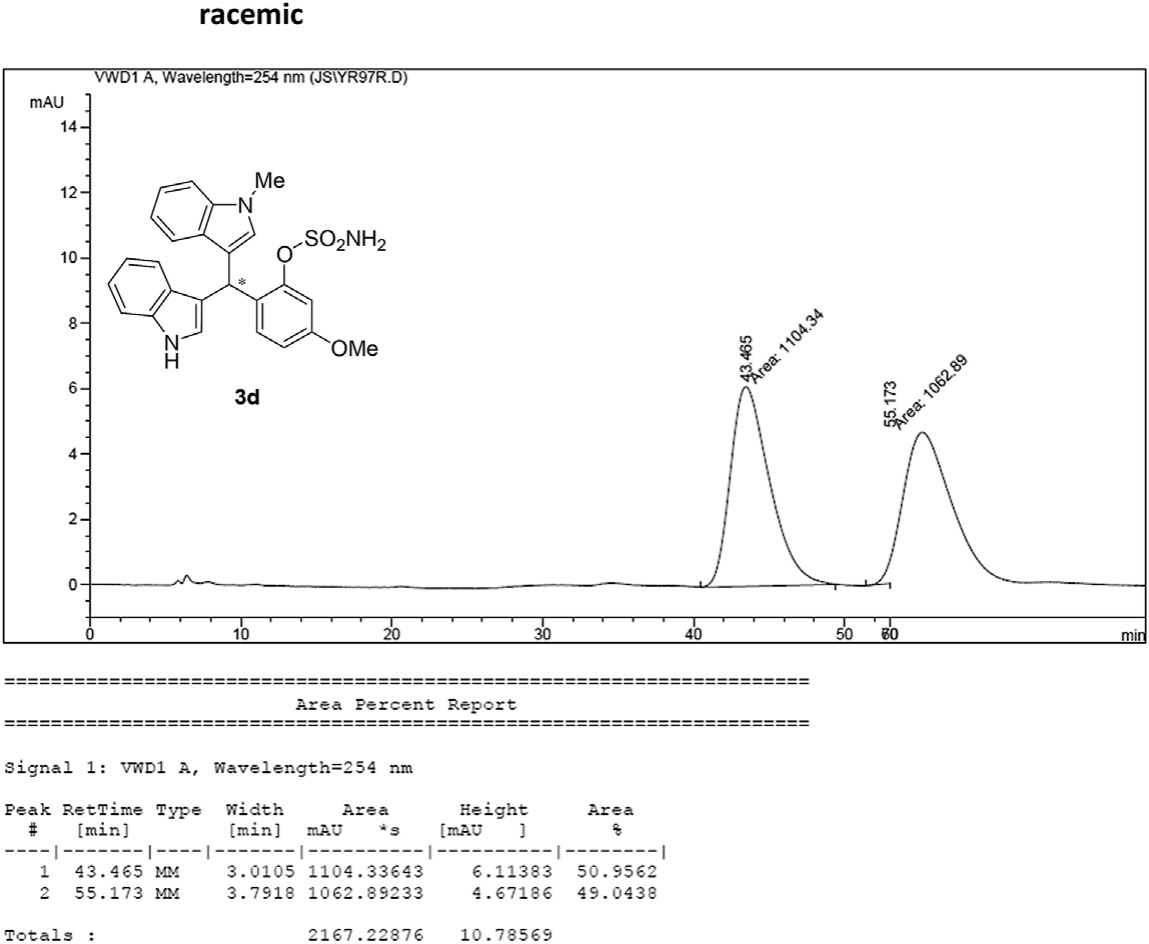

**Figure:**
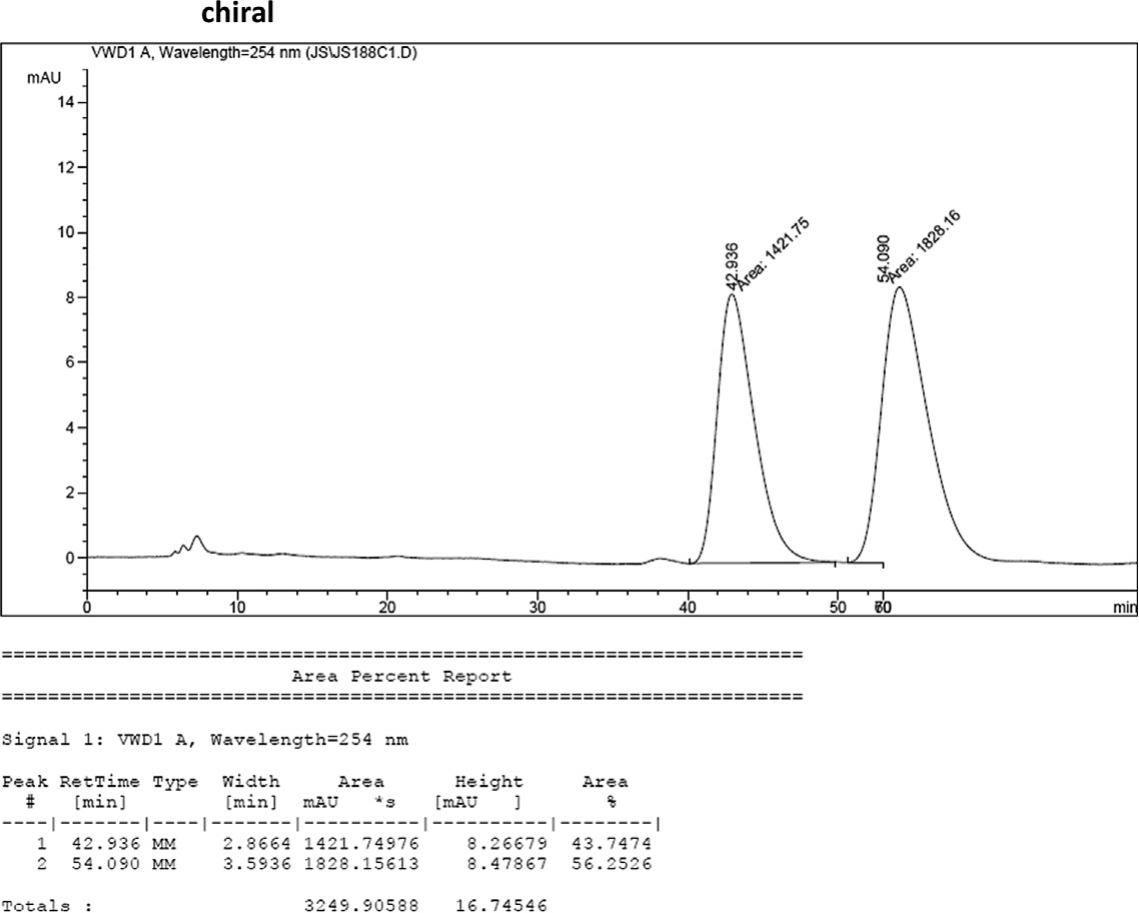

**Figure:**
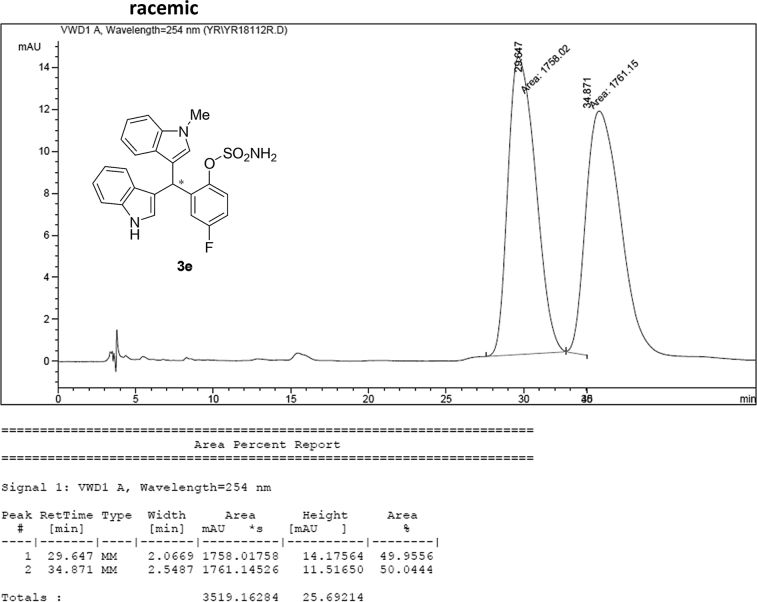

**Figure:**
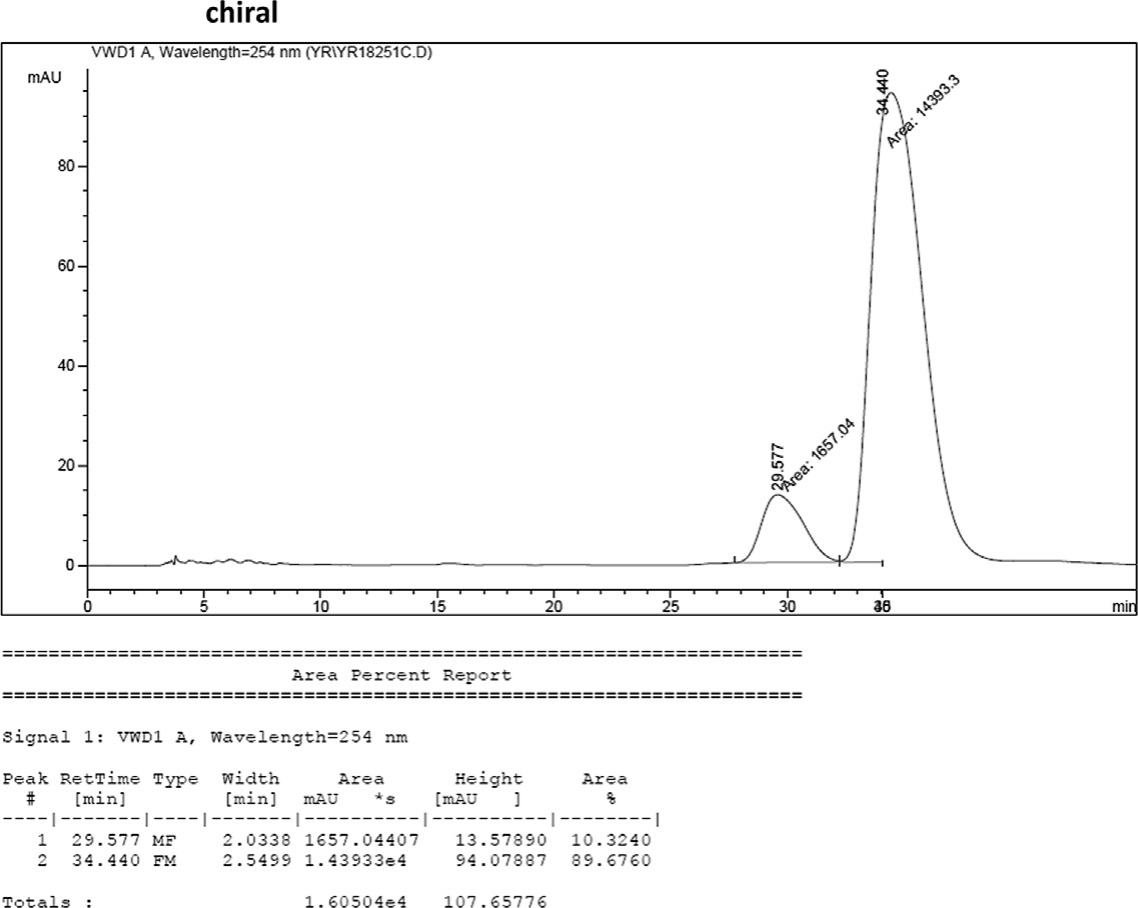

**Figure:**
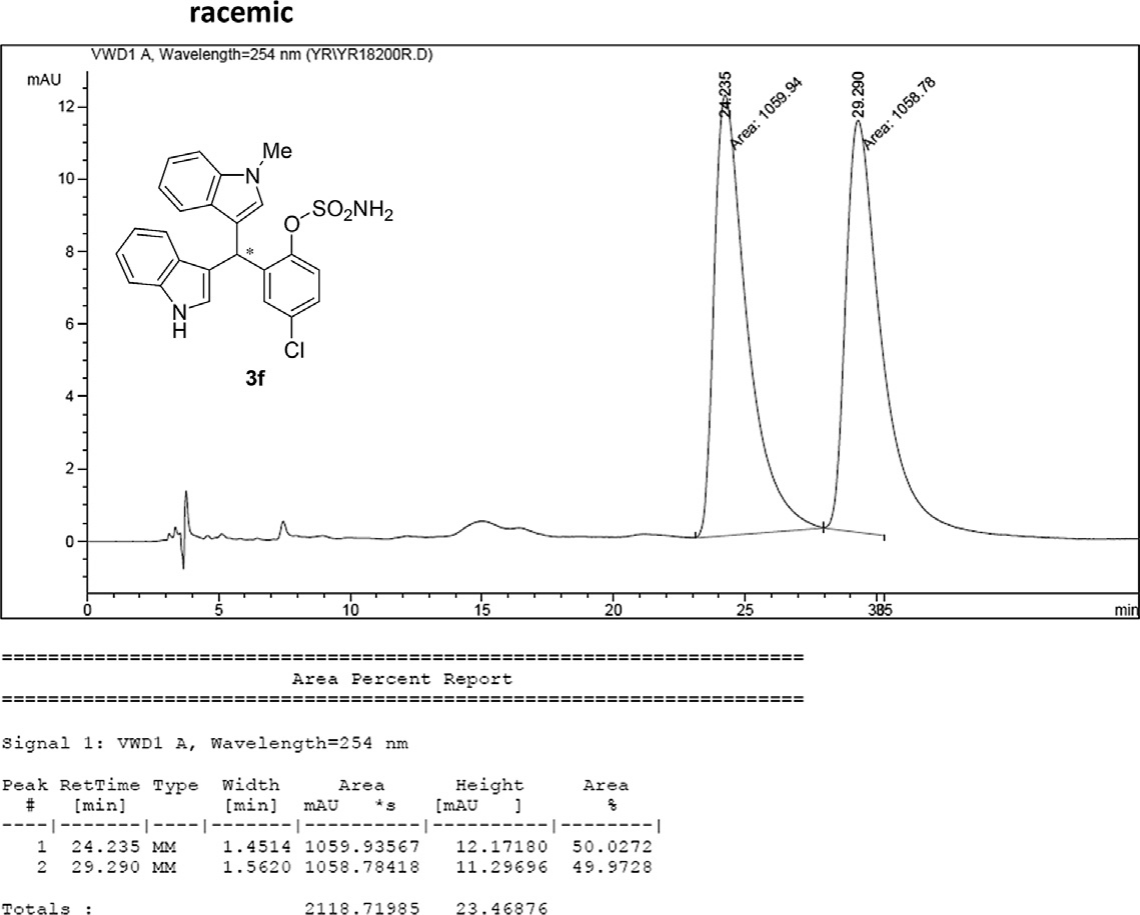

**Figure:**
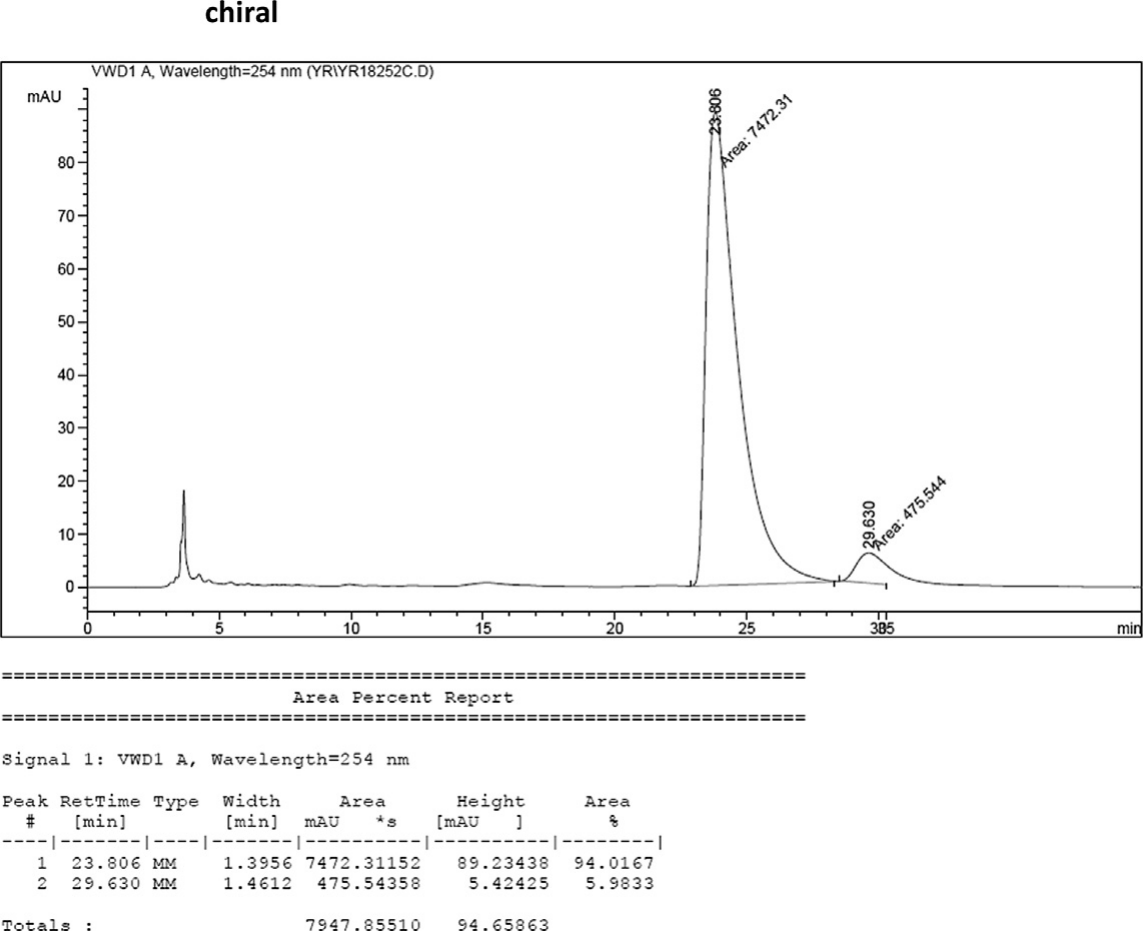

**Figure:**
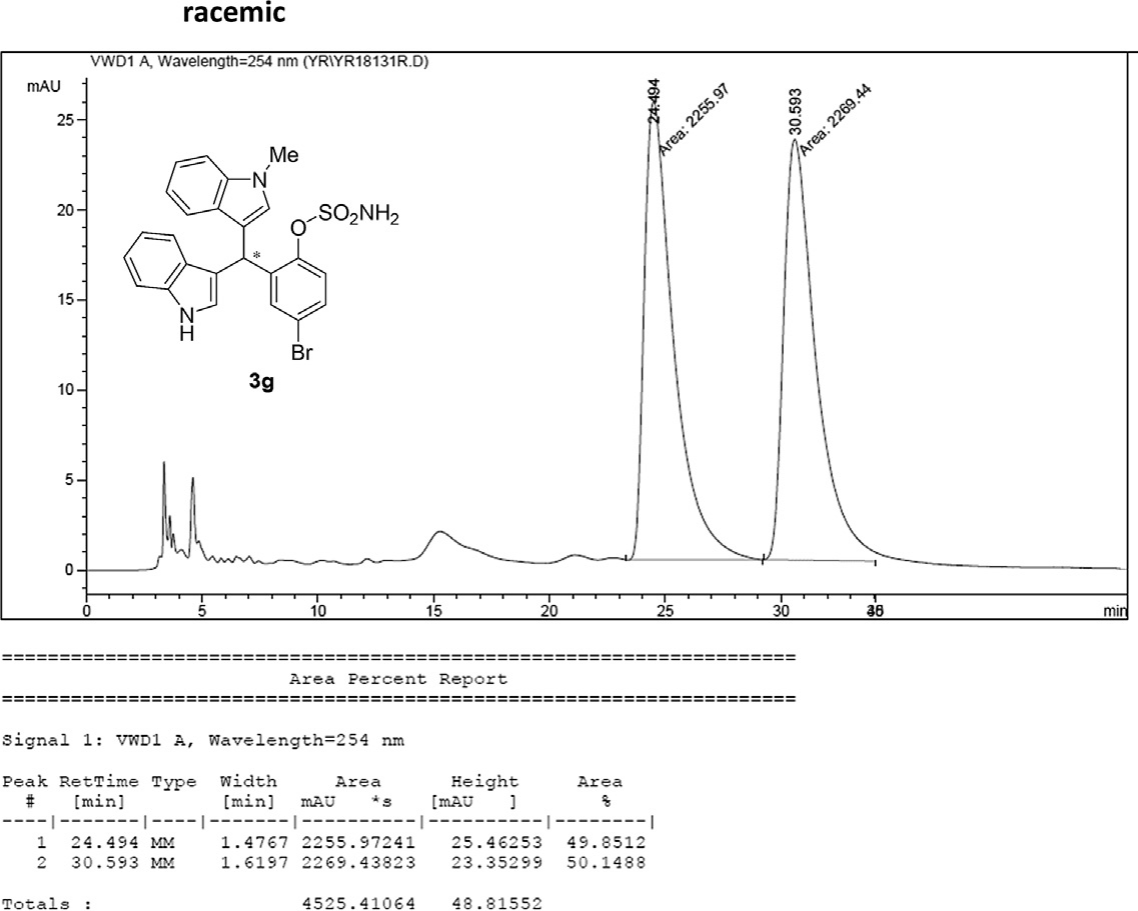

**Figure:**
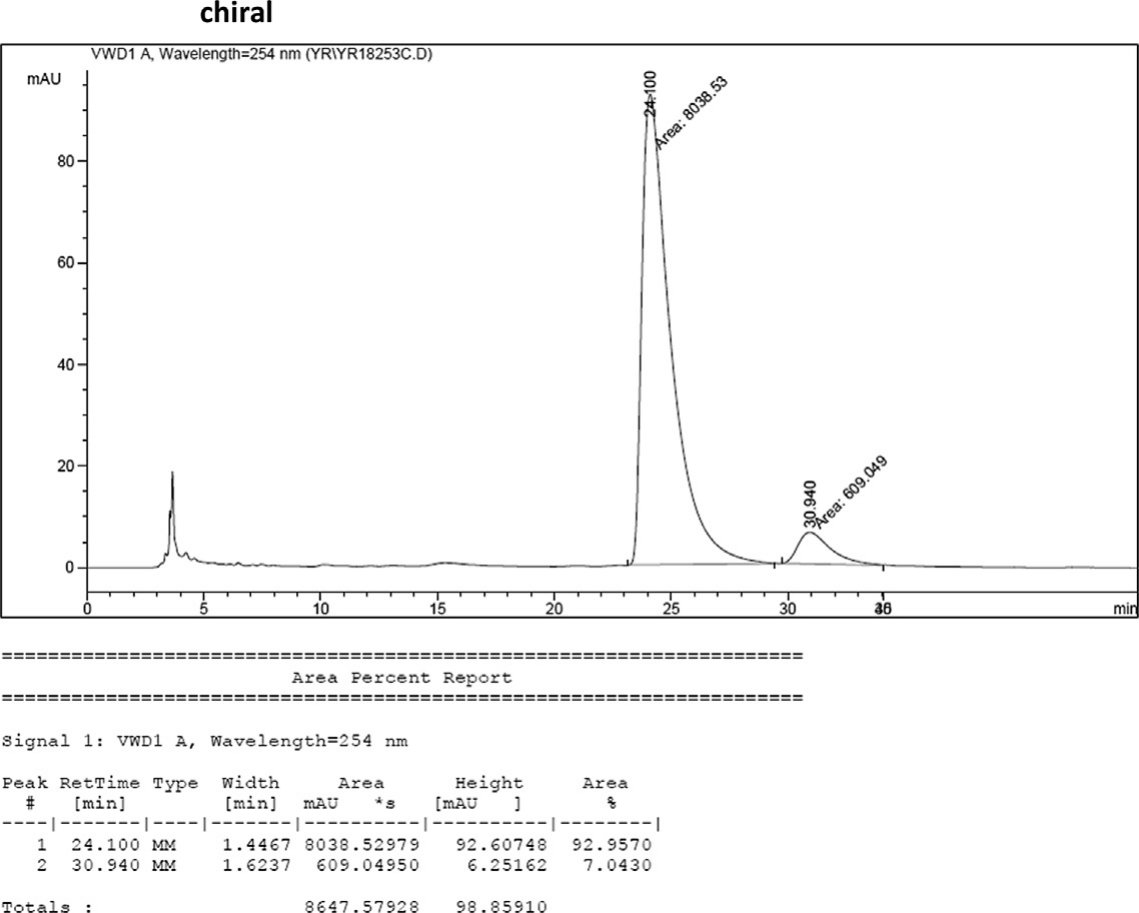

**Figure:**
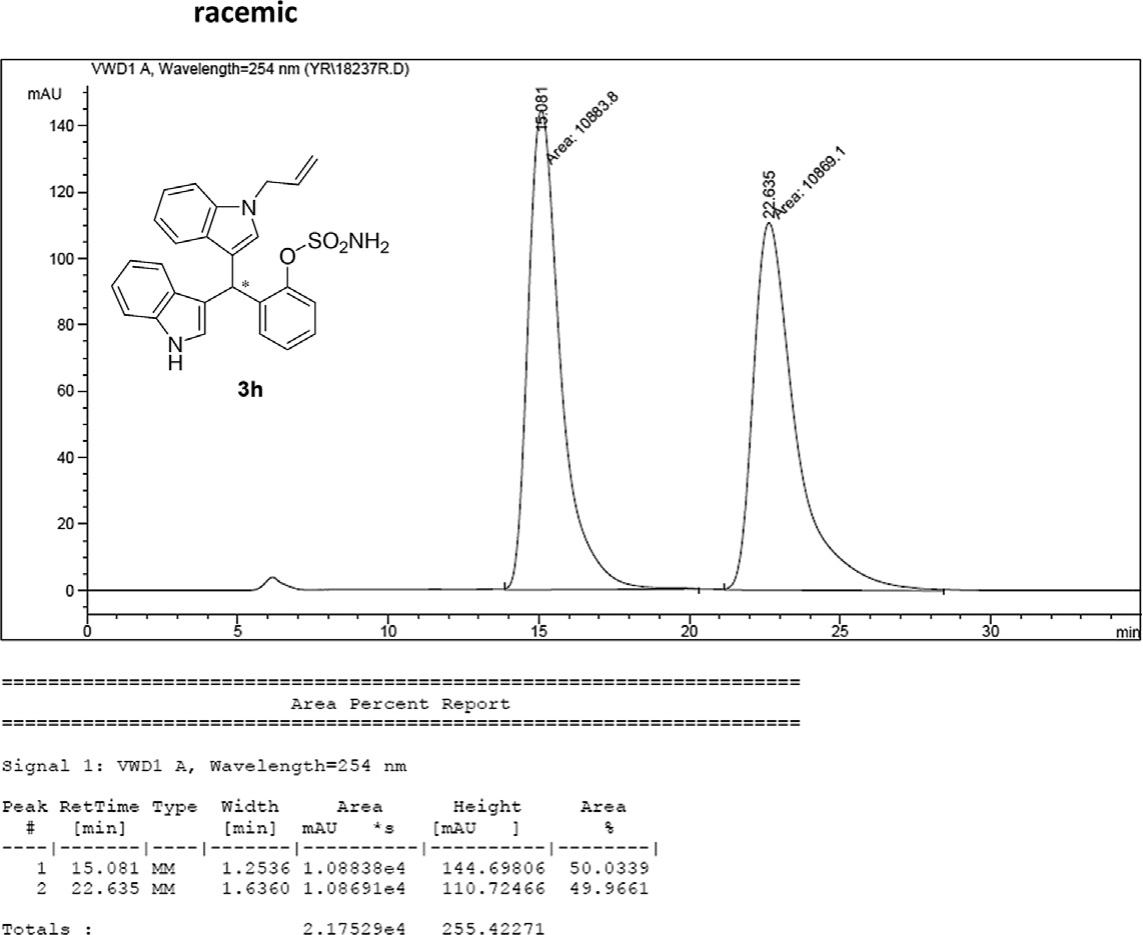

**Figure:**
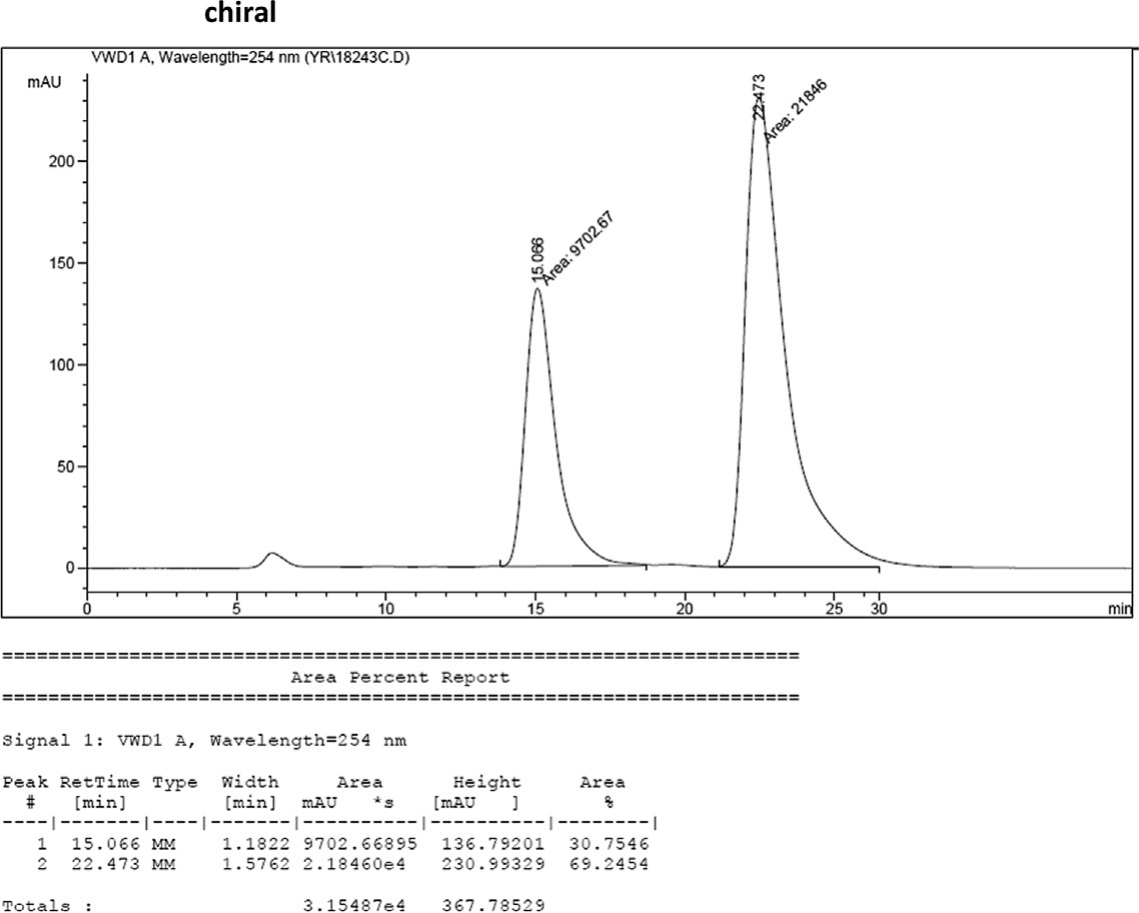

**Figure:**
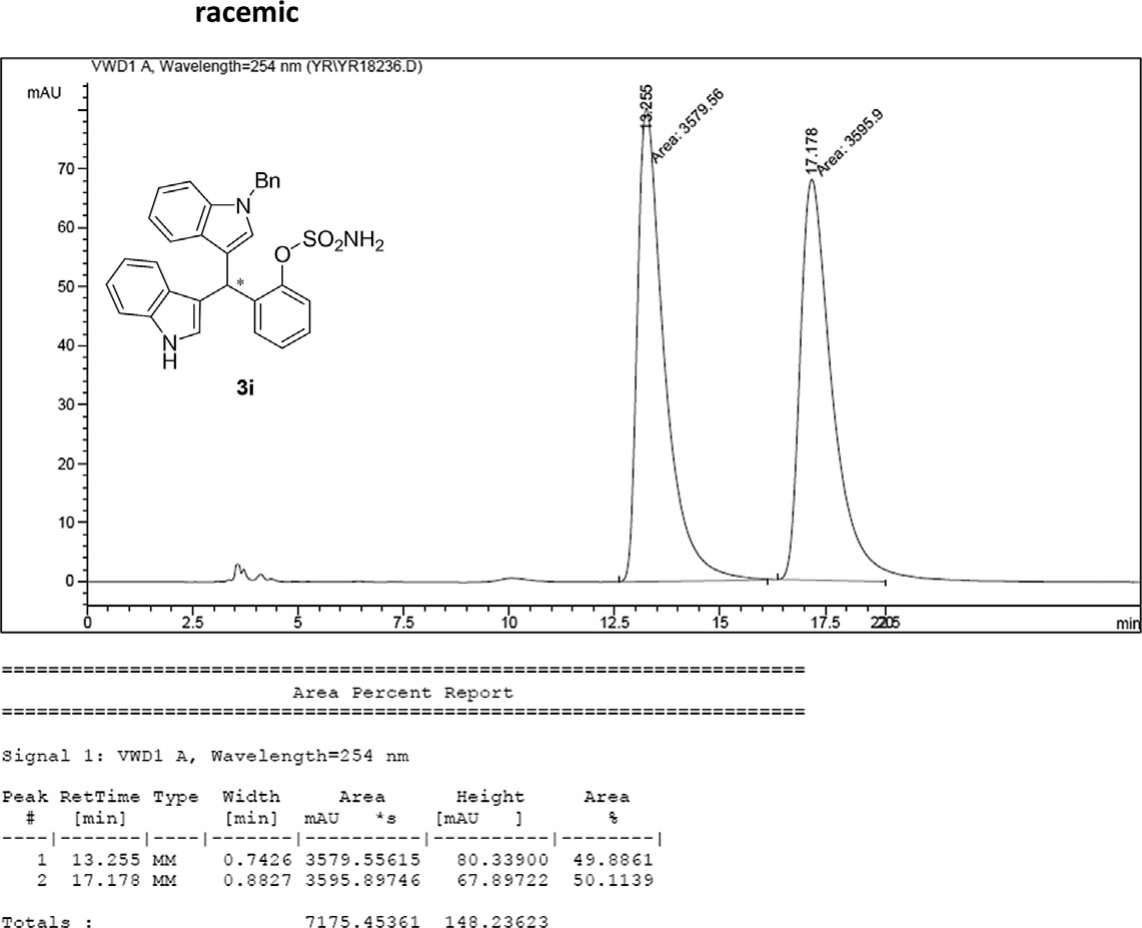

**Figure:**
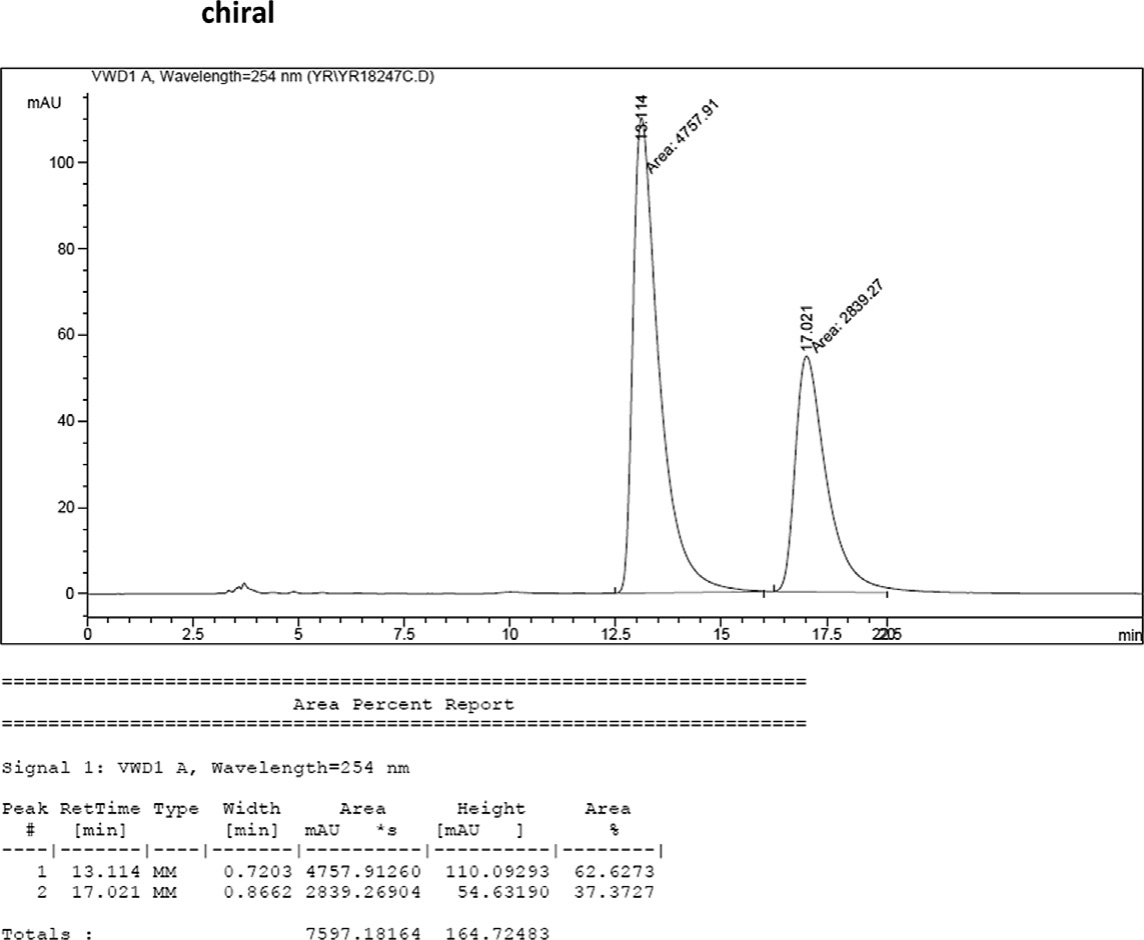

**Figure:**
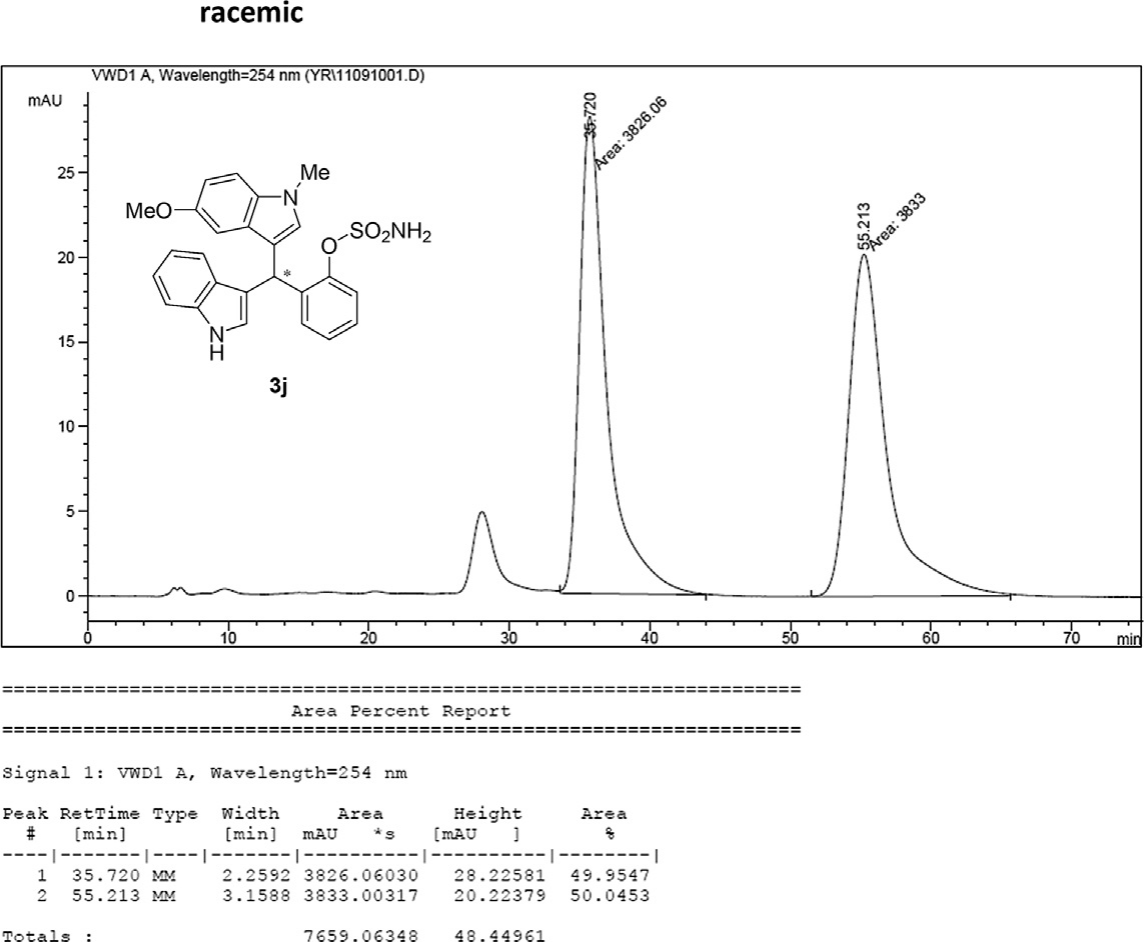

**Figure:**
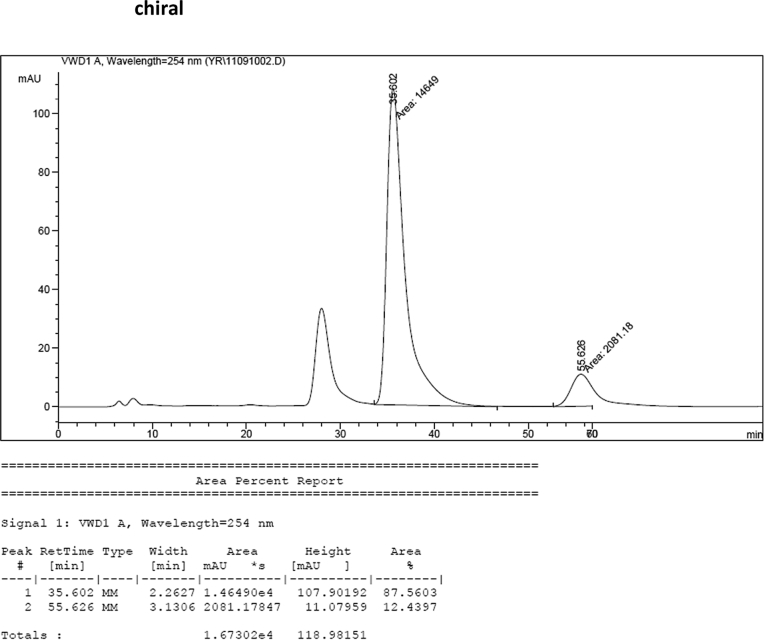

**Figure:**
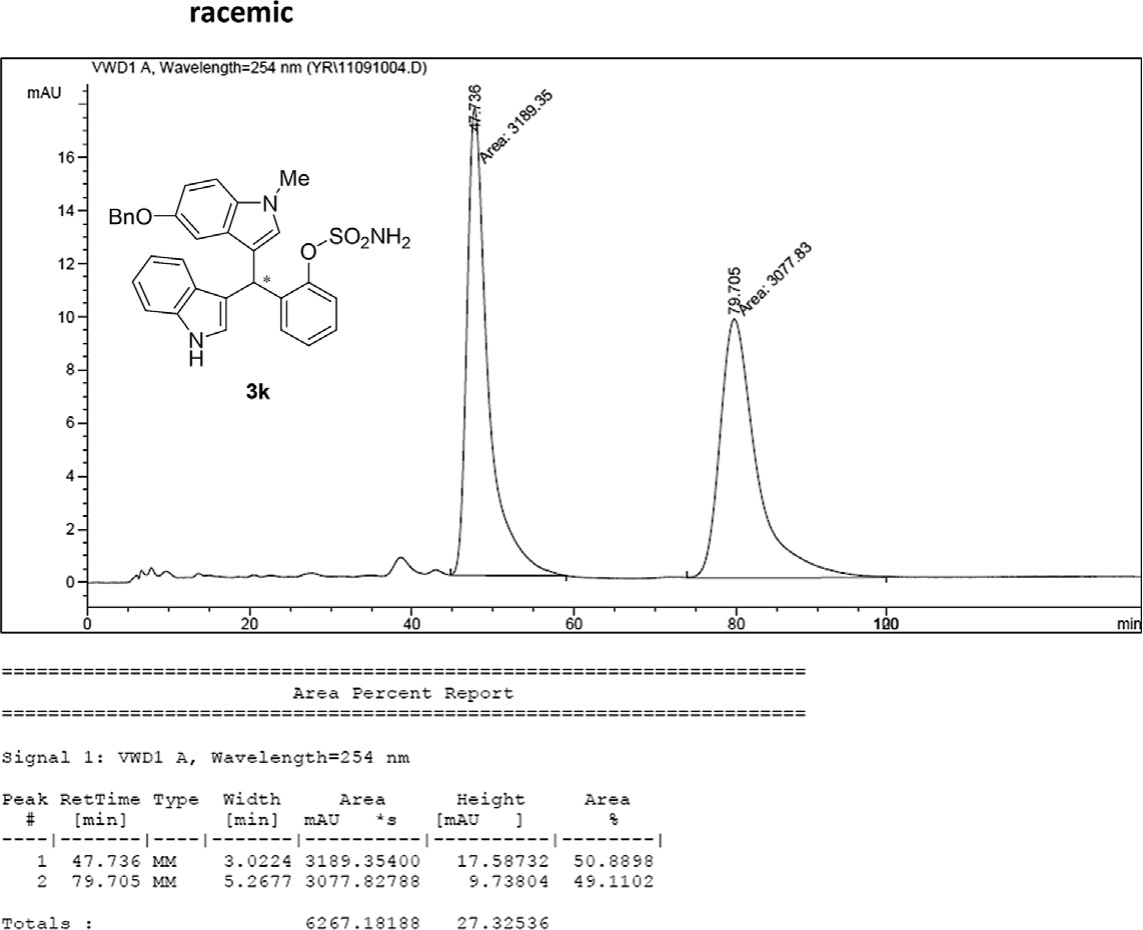

**Figure:**
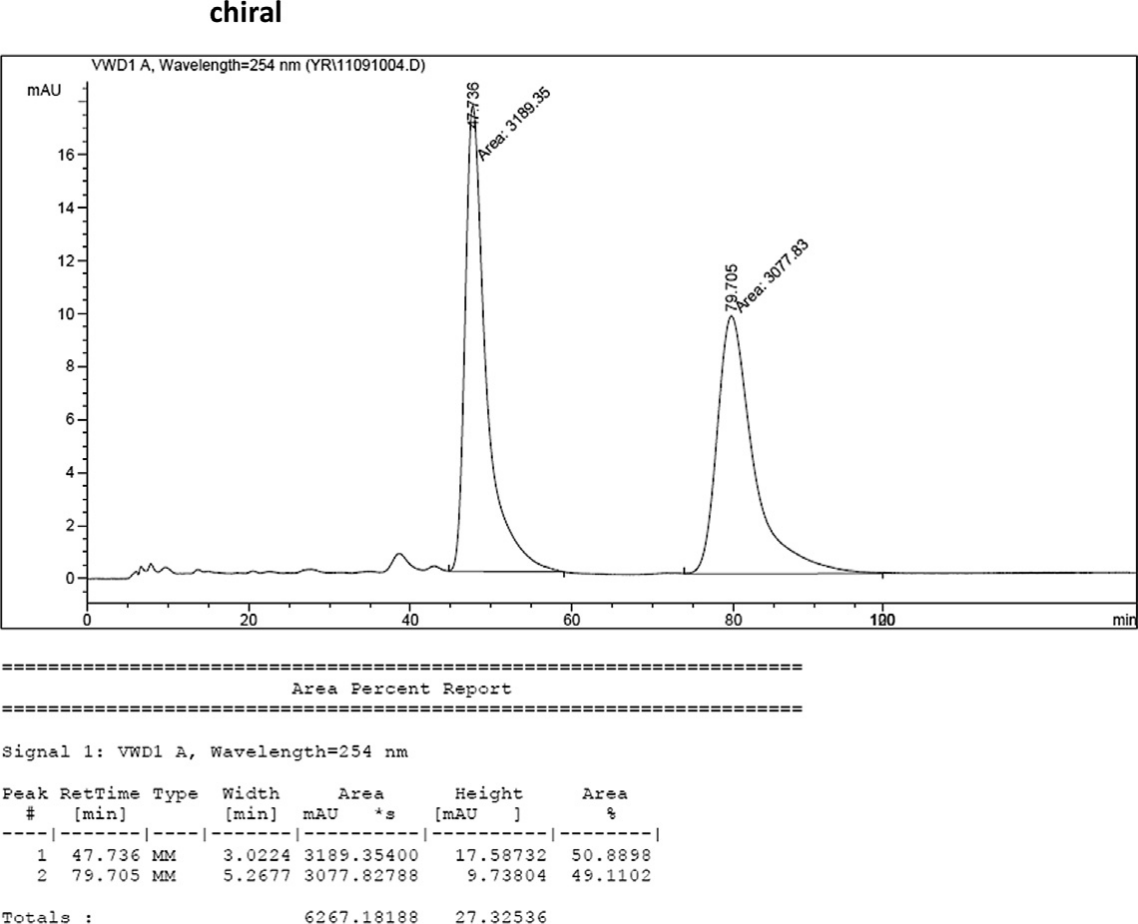

**Figure:**
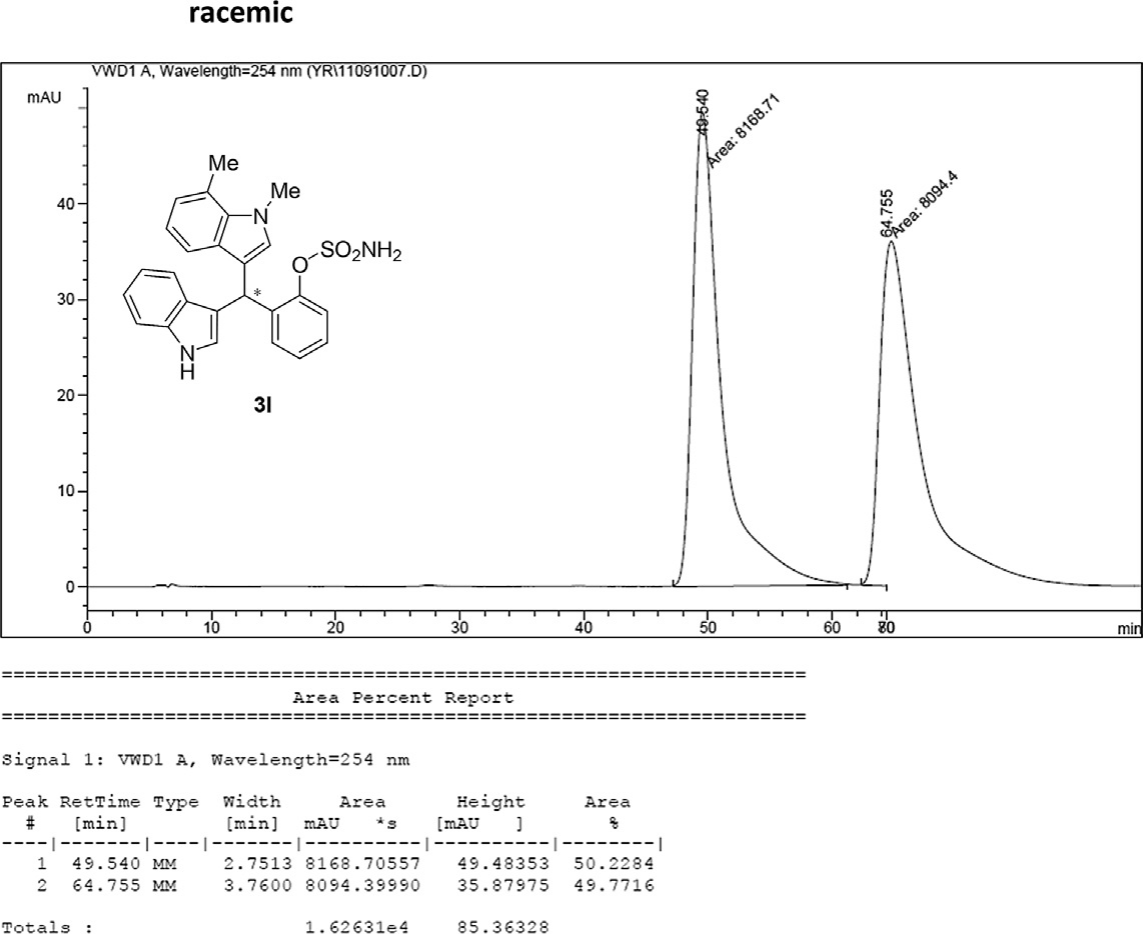

**Figure:**
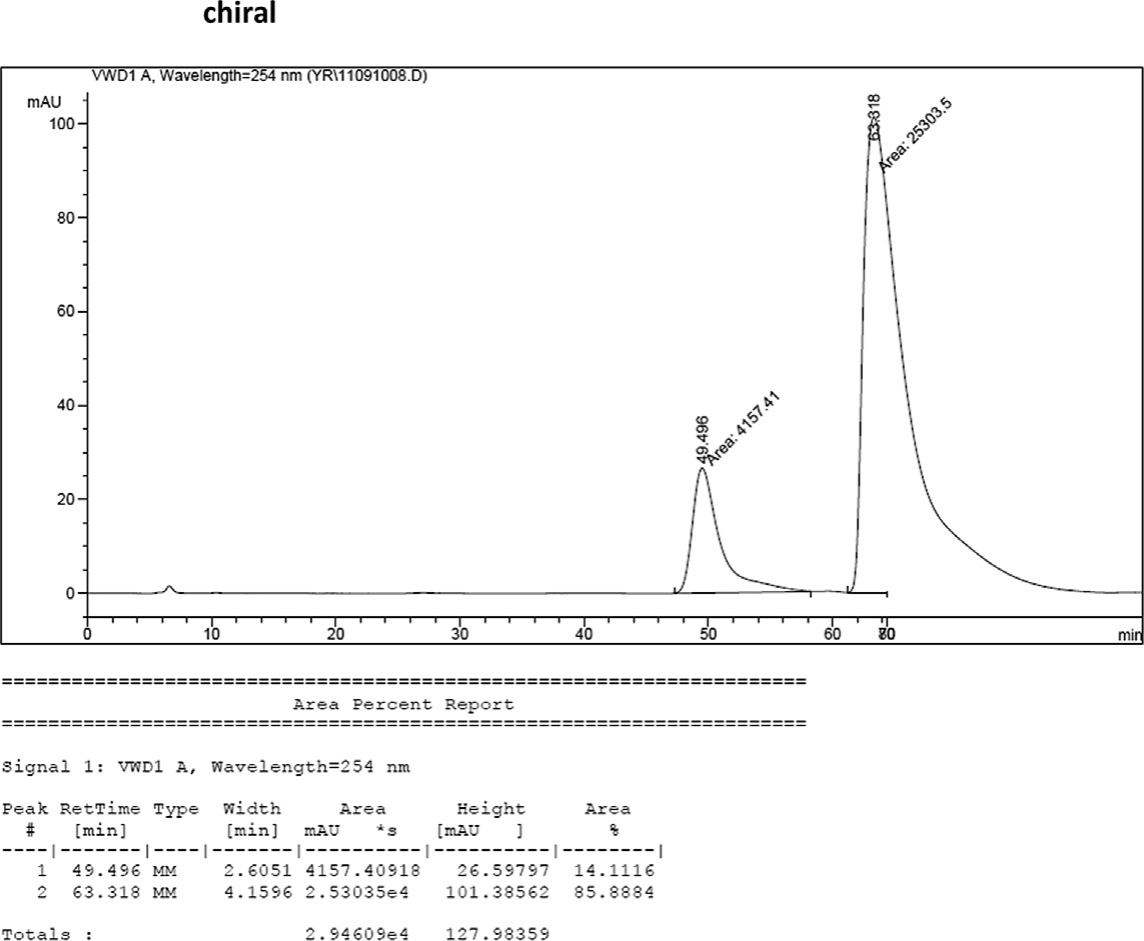

**Figure:**
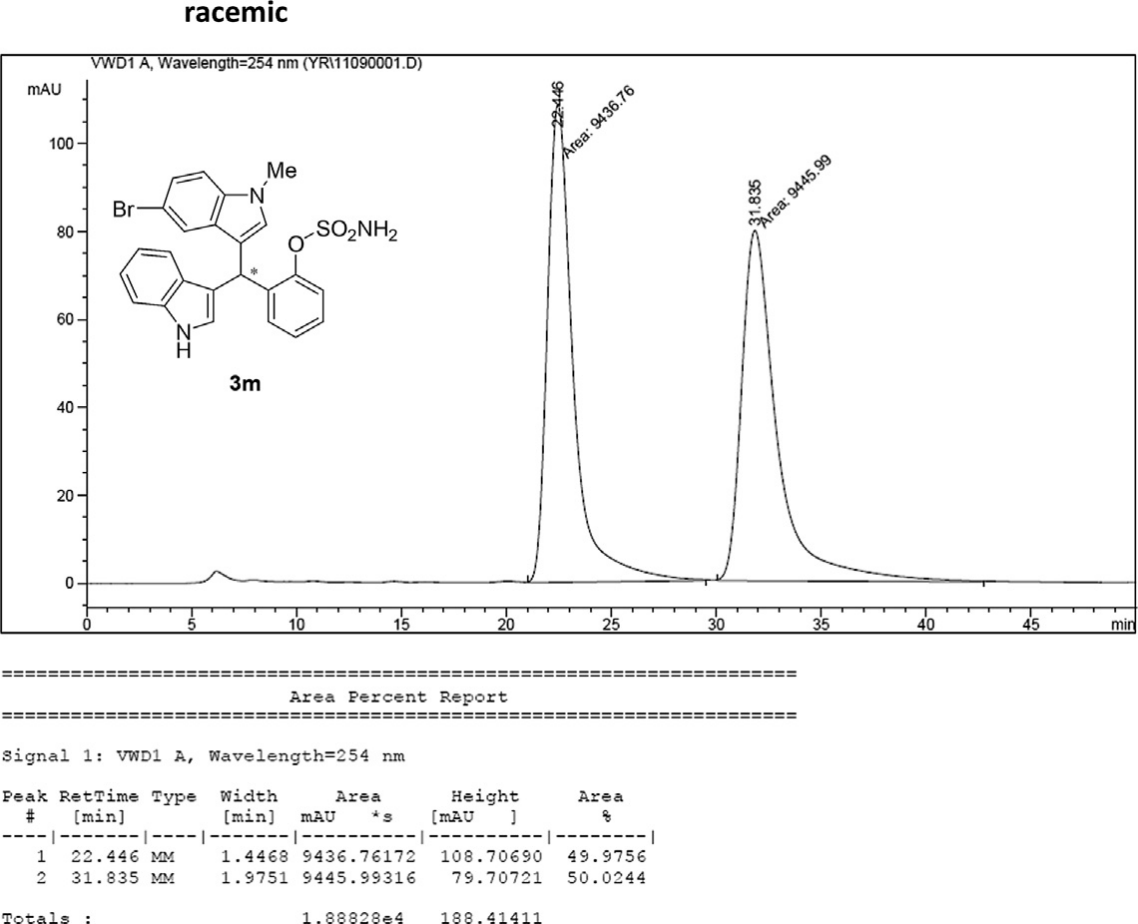

**Figure:**
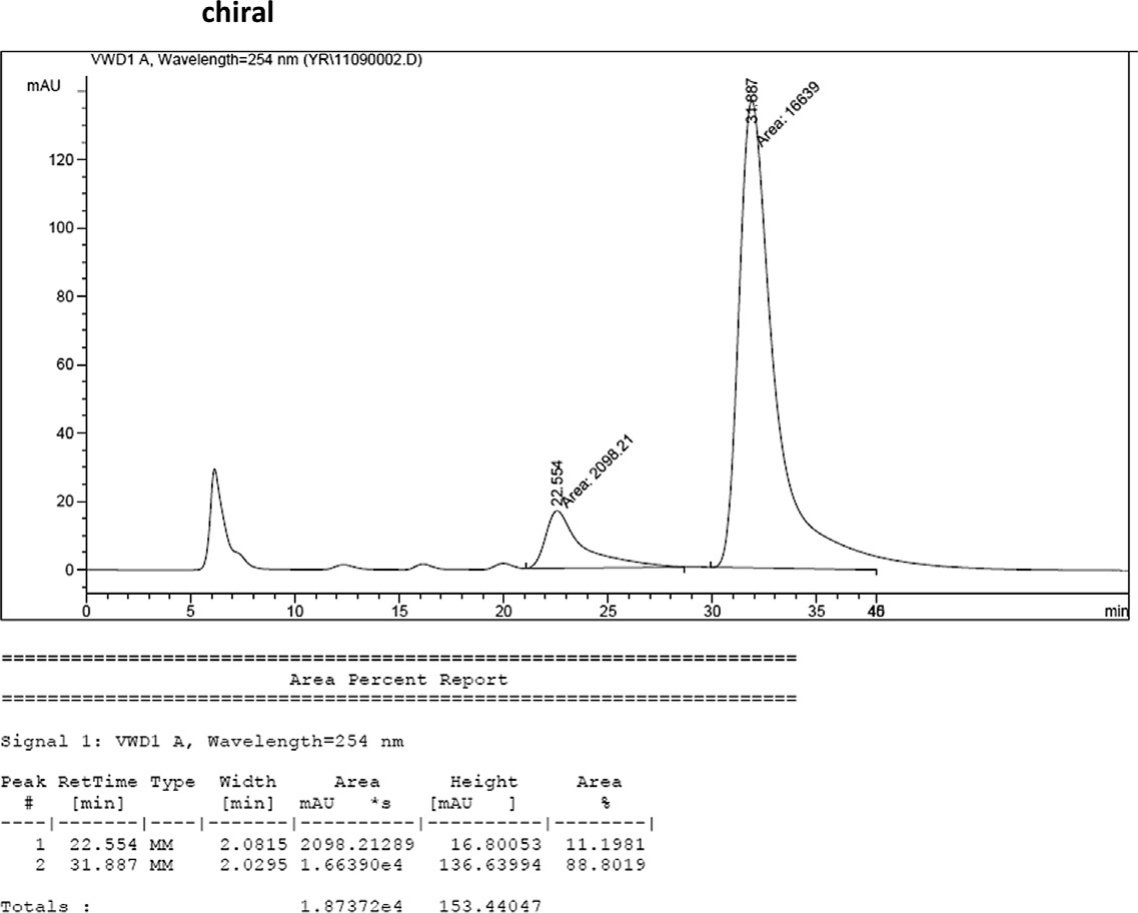

**Figure:**
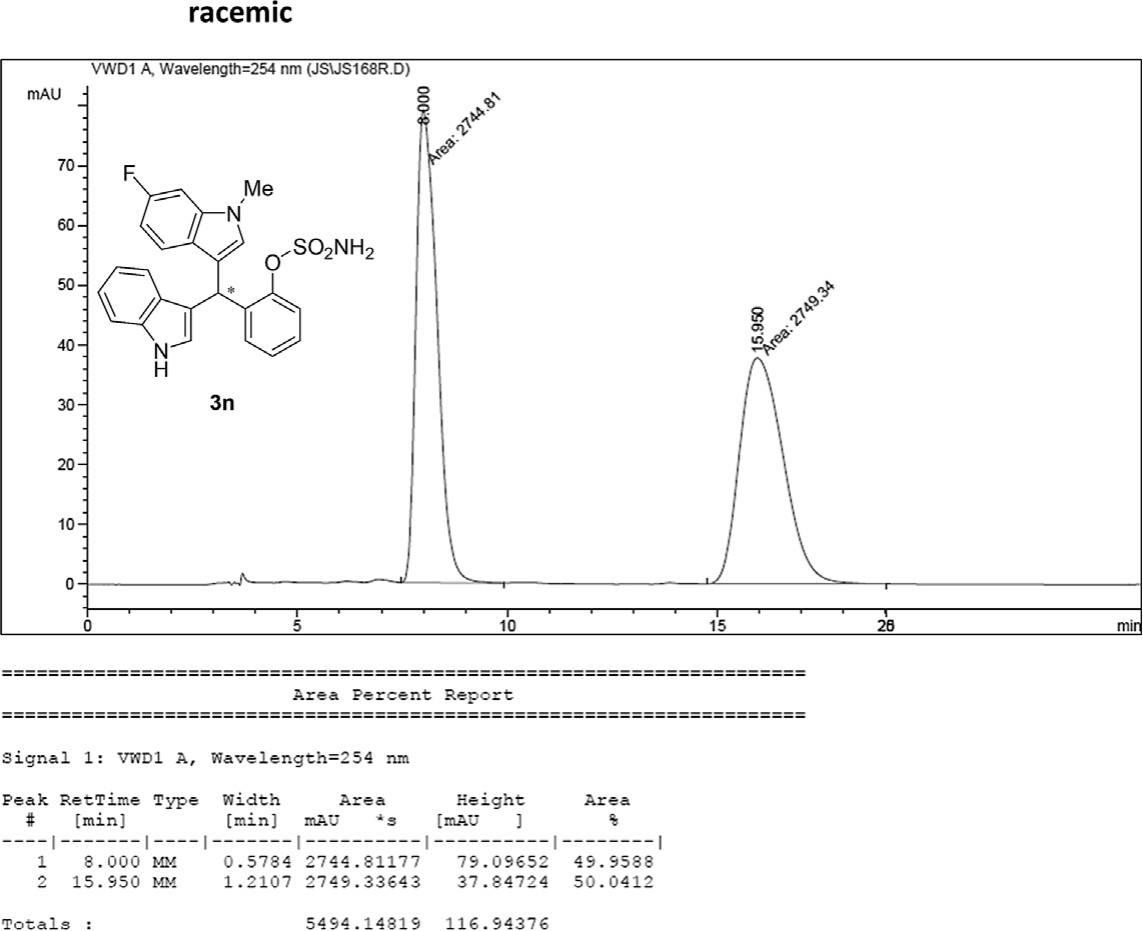

**Figure:**
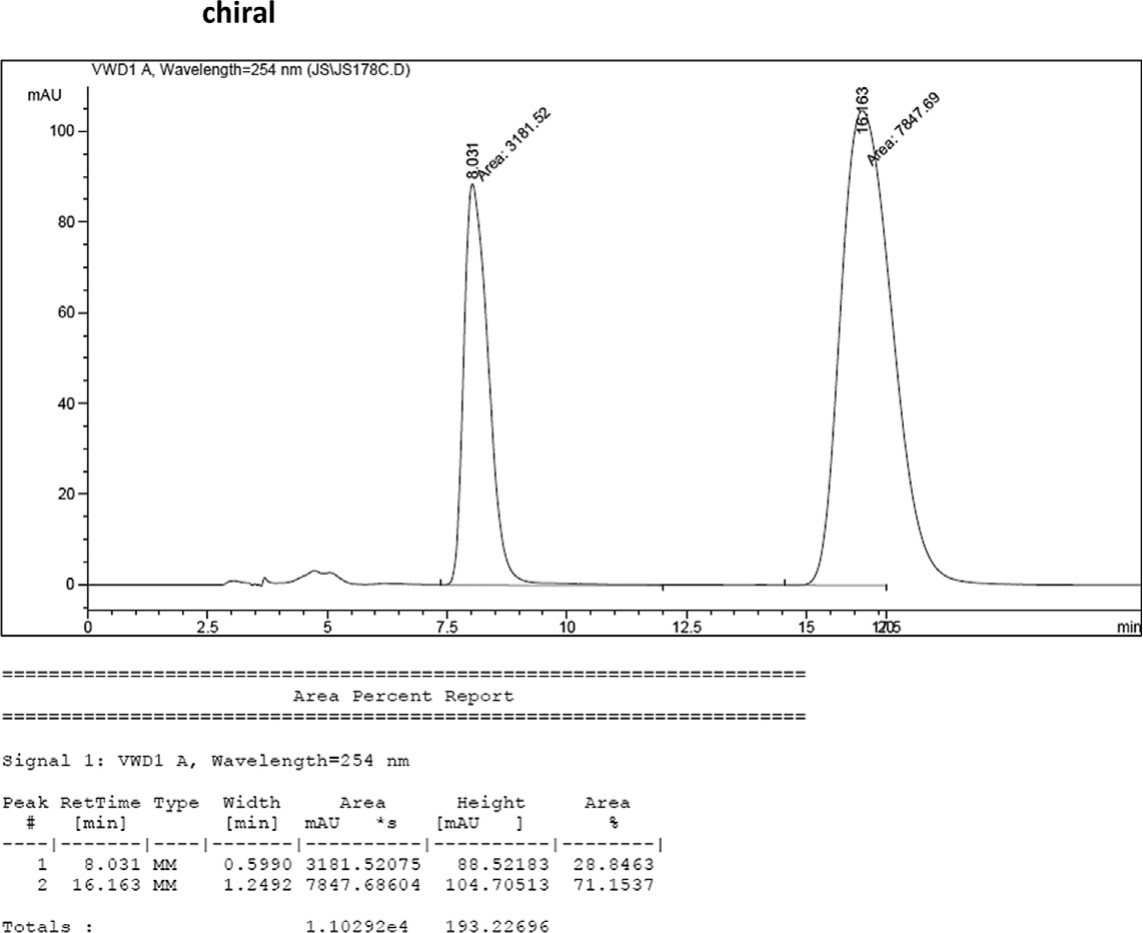

**Figure:**
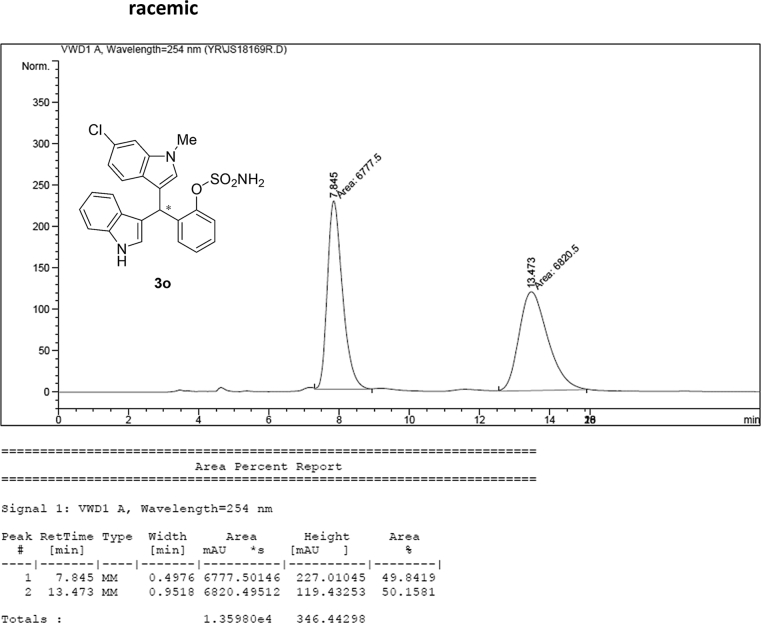

**Figure:**
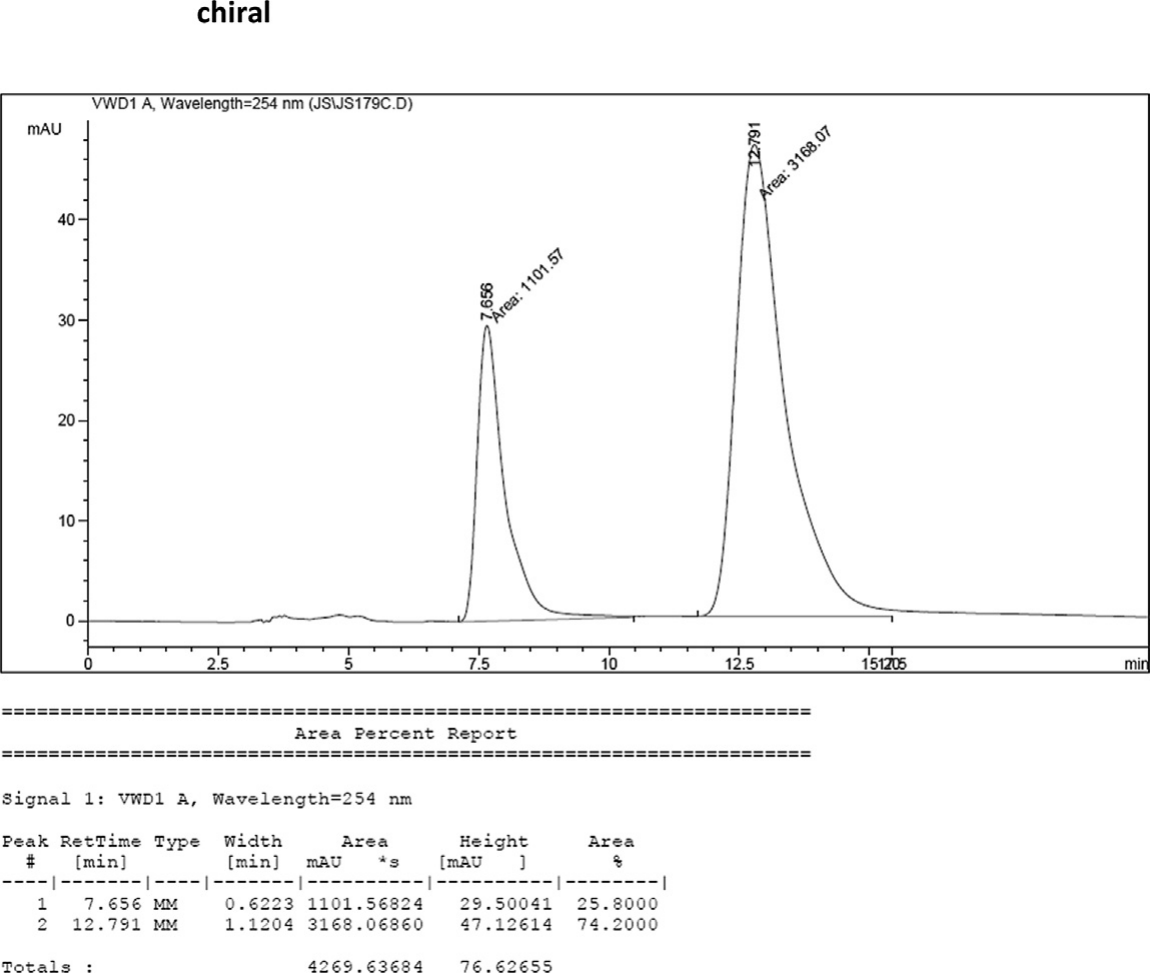

**Figure:**
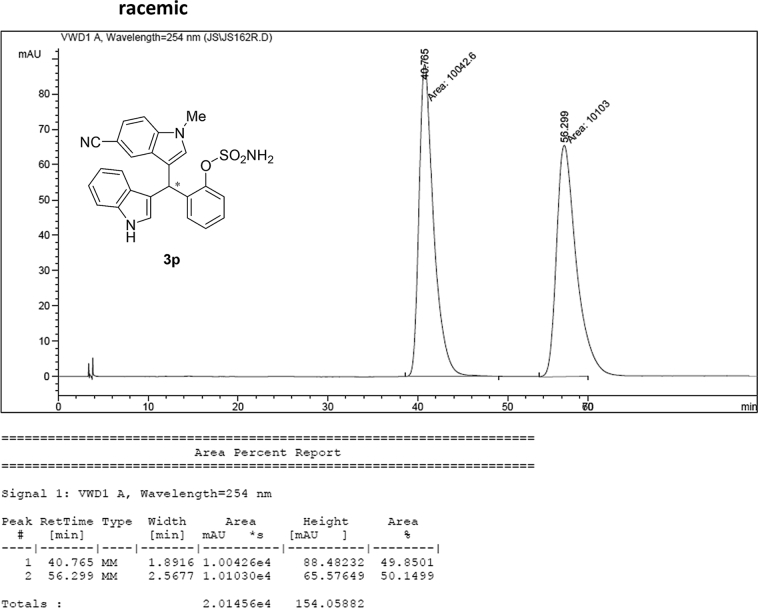

**Figure:**
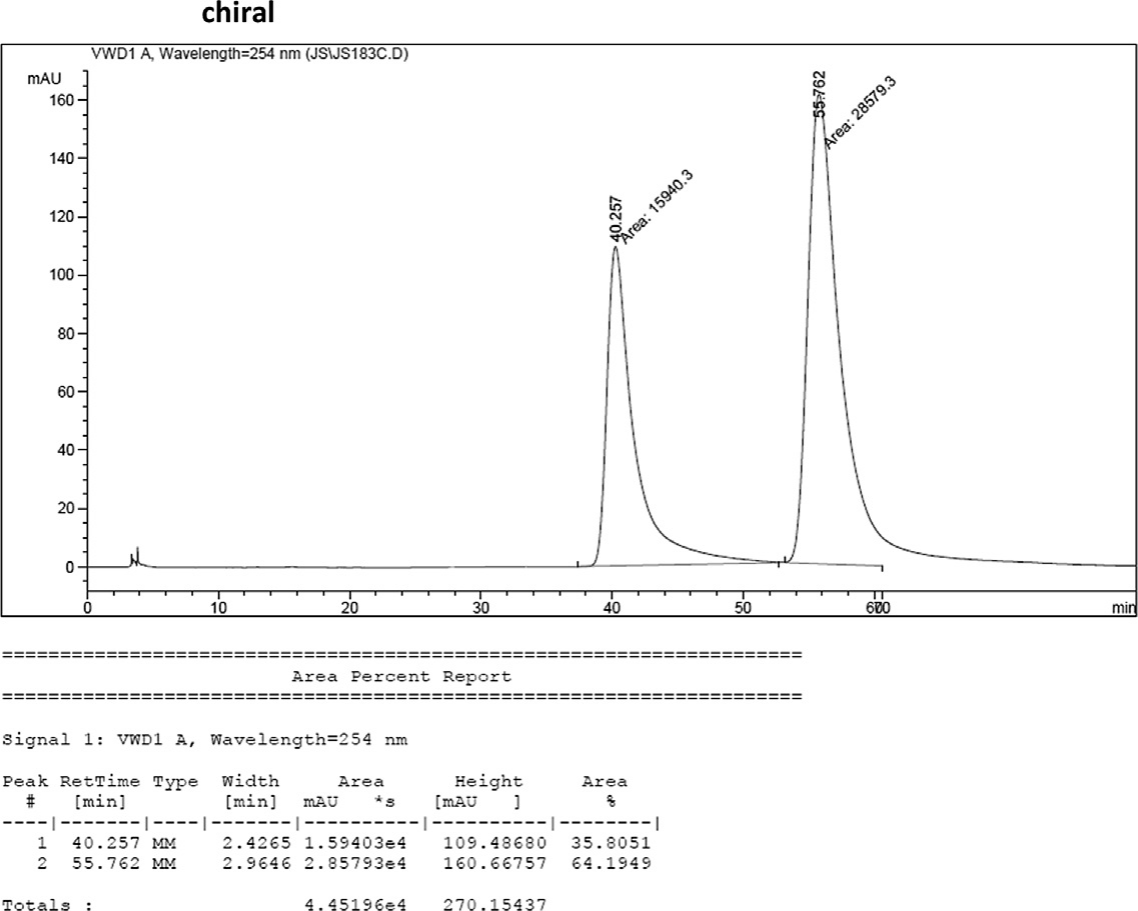

**Figure:**
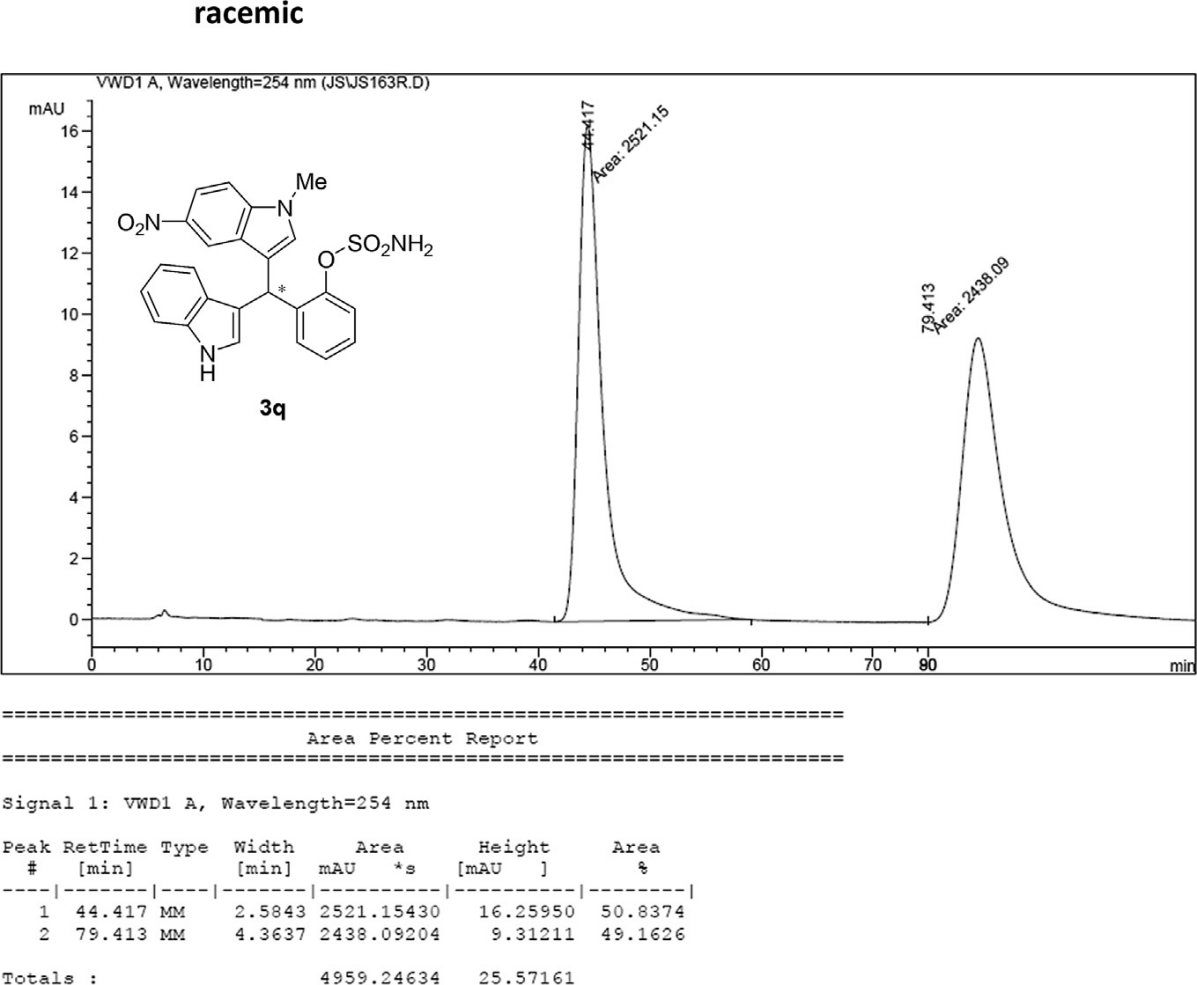

**Figure:**
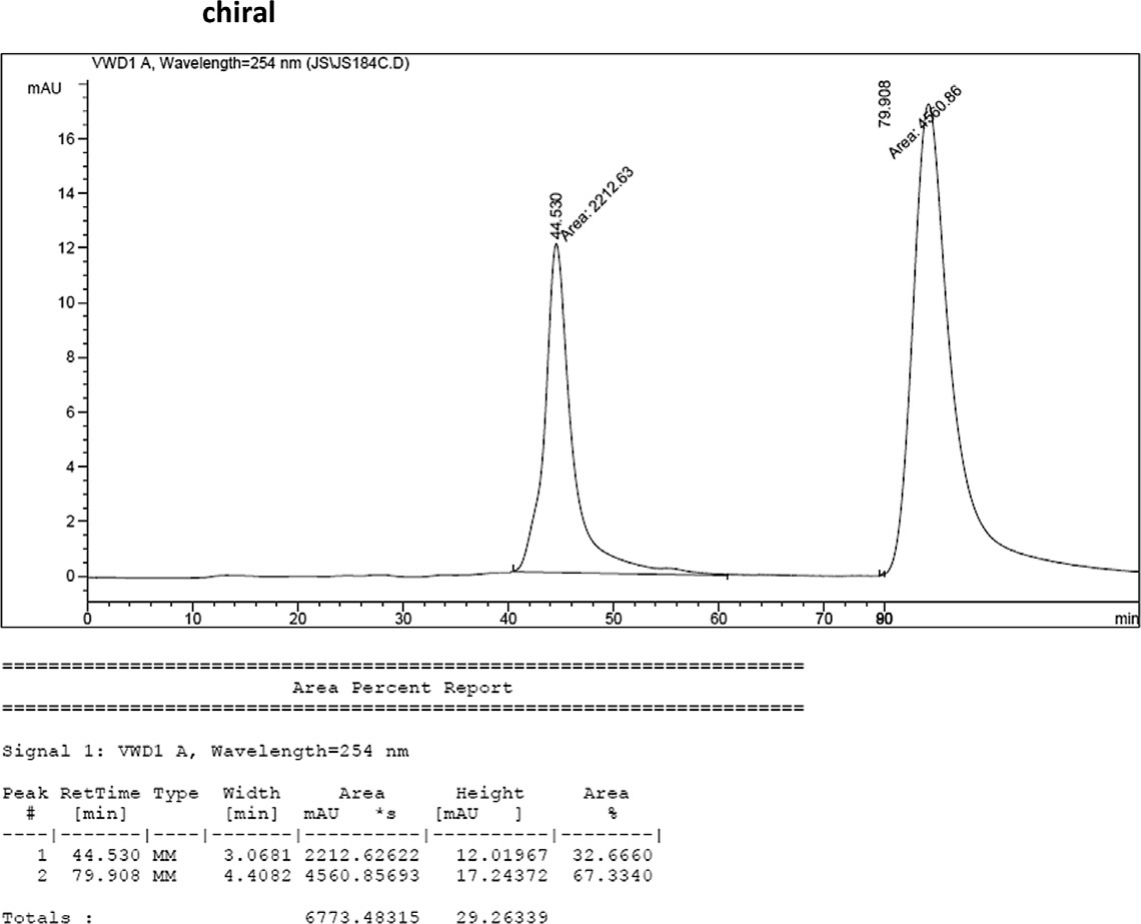

**Figure:**
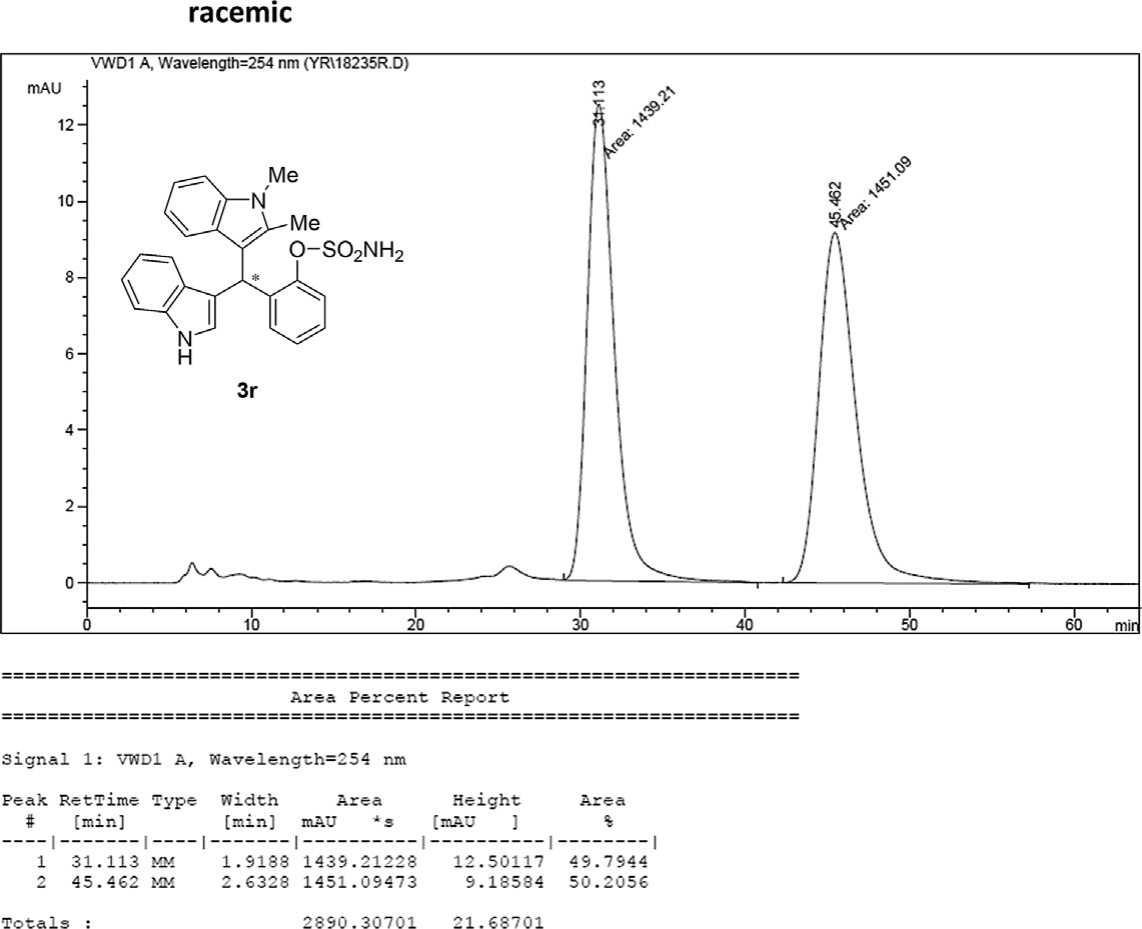

**Figure:**